# Luminescent metal complexes featuring photophysically innocent boron cluster ligands†
†Electronic supplementary information (ESI) available: See ESI for full experimental details. CCDC 1446940–1446945. For ESI and crystallographic data in CIF or other electronic format see DOI: 10.1039/c6sc01146b


**DOI:** 10.1039/c6sc01146b

**Published:** 2016-04-27

**Authors:** Kent O. Kirlikovali, Jonathan C. Axtell, Alejandra Gonzalez, Alice C. Phung, Saeed I. Khan, Alexander M. Spokoyny

**Affiliations:** a Department of Chemistry and Biochemistry , University of California , 607 Charles E. Young Drive East , Los Angeles , California 90025-1569 , USA . Email: spokoyny@chem.ucla.edu ; https://www.organomimetic.com

## Abstract

We report the synthesis and characterization of a series of d^8^ metal complexes featuring robust and photophysically innocent strong-field chelating 1,1′-bis(*o*-carborane) (**bc**) ligand frameworks. A combination of UV-Vis spectroscopy, single crystal X-ray structural analysis, and DFT calculations of these species suggest that the dianionic **bc** ligand does not contribute to any visible metal-to-ligand charge transfer (MLCT) transitions, yet it provides a strong ligand field in these complexes. Furthermore, a **bc**-based Pt(ii) complex containing a 4,4′-di-*tert*-butyl-2,2′-bipyridine ligand (dtb-bpy) has been prepared and was found to display blue phosphorescent emission dominated by MLCT from the Pt(ii) center to the dtb-bpy ligand. Importantly, the bulky three-dimensional nature of the **bc** ligand precludes intermolecular Pt(ii)···Pt(ii) interactions in the solid state where the resulting compounds retain their emission properties. This study opens a potentially new avenue for designing organic light-emitting diode (OLED) materials with tunable properties featuring photophysically innocent boron-rich cluster ligands.

## Abstract 2

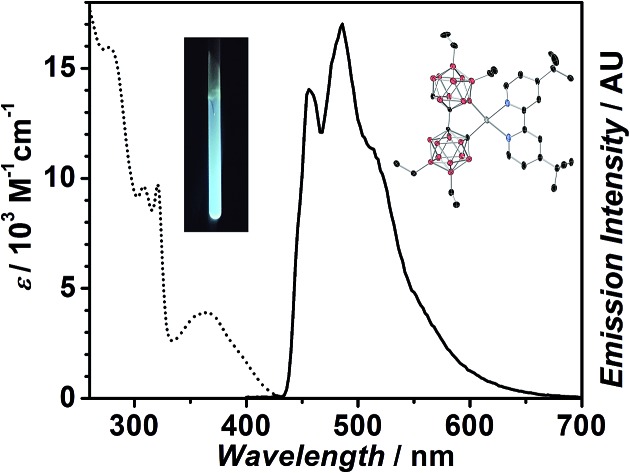
We report the synthesis and characterization of a series of d^8^ metal complexes featuring robust and photophysically innocent strong-field chelating 1,1′-bis(*o*-carborane) (**bc**) ligand frameworks.

## Introduction

Many square planar platinum complexes containing chelating biaryl ligands have been established as efficient phosphorescent emitters, making them desirable dopants in organic light-emitting diodes (OLEDs).^1^,^2^ The emissive properties of these Pt(ii) complexes can be tuned by varying the electronic nature of the ligands surrounding the metal center; however, complete control over desired photophysical properties is still limited. For example, these square planar complexes are susceptible to aggregation as a consequence of the lack of steric bulk above and below the square plane, leading to intermolecular Pt(ii)···Pt(ii) interactions that bring about luminescence quenching and red-shifting of the solid-state emission frequency.^2^,^3^ Furthermore, in prototypical mixed ligand complexes, (L)(L′)Pt(ii), the HOMO is generally both metal- and ligand-based (Pt(ii) and L) while the LUMO is located on the remaining ligand (L′).^4^ This ultimately complicates the predictability of ligand effects on emission properties. A class of compounds that could kinetically stabilize the Pt(ii) square planar framework without participating in electronic transitions would prove very useful for creating improved phosphorescent emitters for the next generation of OLED devices.

Icosahedral dicarba-*closo*-dodecaboranes (C_2_B_10_H_12_, carborane) are robust, charge-neutral boron clusters that are often viewed as 3D aromatic analogues of arenes. Unfunctionalized carborane species have an extremely large HOMO–LUMO gap (∼8 eV, see ESI†),^5^ making them potentially useful building blocks for probing their photophysical innocence in the context of metal-based phosphorescent emitters. This is especially appealing given the available functionalization routes through either carbon or boron vertices in these clusters, enabling the synthesis of tailored ligand frameworks for transition metal complexes.^6^ For example, Lee and co-workers have recently demonstrated that κ^2^-C,N-bound 1-(2-pyridyl)-*o*-carboranyl7*a* and κ^2^-C,P-bound 1-(^i^Pr_2_PCH_2_)-*o*-carboranyl7*b* can be strong ancillary ligands that contribute to the electronic stabilization of bis(heteroleptic) Ir(iii) species (see ESI† for molecular structures), leading to an arylpyridine-dominant phosphorescent emission. From DFT calculations and analysis of the emission spectra, these authors determined the C-bound *o*-carboranyl unit remains uninvolved in electronic transitions and that phosphorescent emission results from MLCT of the Ir(iii)-based HOMO to the arylpyridine-based LUMO. Furthermore, several groups have functionalized biaryl ligands with C-connected carboranyl moieties (*ortho*, *meta*, *para*, and *nido*) to tune luminescent properties^8^ (see ESI† for molecular structures). To the best of our knowledge, attempts to design a tunable, exclusively carborane-based ligand scaffold for phosphorescent emitter molecules have not been explored thus far.^9^ Such a ligand framework would be an ideal system for a rational design of metal-based luminescent complexes (*vide supra*).

**Fig. 1:**
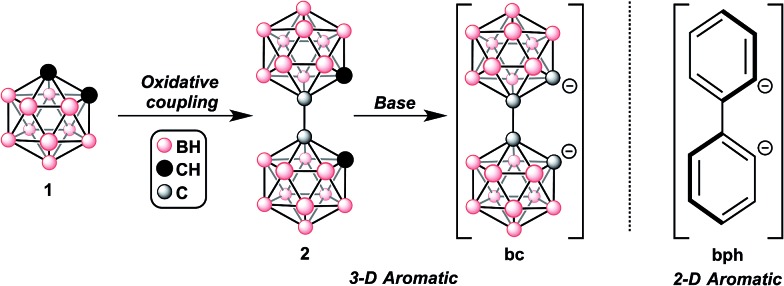
Oxidative coupling of *o*-carborane (**1**) yields 1,1′-bis(*o*-carborane) (**2**). Deprotonation of **2** yields the dianionic **bc** ligand, which can be conceptually thought of as a 3D aromatic analogue of a dianionic biphenyl ligand (**bph**).

In 1964, Hawthorne reported the first synthesis of 1,1′-bis(*o*-carborane) (**2**),10*a* effectively a 3D analogue of biphenyl (Fig. 1), and in 1973, Zakharkin showed that the oxidative coupling of two *o*-carboranes (**1**) through carbon vertices yields **2**.10*d* Later, Hawthorne demonstrated that the deprotonation of **2** results in a dianionic species **bc**, which was shown to bind several transition metals in bidentate or monodentate fashions.10b–f Ligand **bc** possesses similar electronic and physical properties as the parent *o*-carborane (see ESI†), and behaves as a robust transition metal ligand. More recently, several groups have improved the synthesis of **2** (ref. 10*j*) and further expanded the series of heteroleptic late-transition metal complexes containing **bc**.^11^ However, fundamental electronic characterization and potential applications for these compounds as electronic materials have yet to be disclosed.

Two monoanionic bidentate ligands are commonly used in Pt(ii) architectures employed for OLEDs, but there are few examples of Pt(ii)-based emitters containing a dianionic bidentate ligand and a neutral bidentate ligand.^12^ One such example is Pt(bph)(bpy)12a–c (where bph = biphenyl, bpy = 2,2′-bipyridine, see ESI† for a molecular structure). We hypothesized that employing **bc** in place of bph could introduce sufficient steric bulk above and below the square plane to eliminate intermolecular Pt(ii)···Pt(ii) interactions, which are often responsible for non-radiative decay pathways that lower luminescent efficiency.^2^,^3^ Additionally, the lack of an exposed π-aromatic system in **bc** should help in reducing these undesirable intermolecular interactions and unwanted substitution and degradation pathways. Furthermore, the high-lying LUMO of **bc** should be inaccessible for orbital mixing and MLCT processes. Therefore, the **bc** ligand was hypothesized to provide kinetic stability while maintaining photophysical innocence in the context of designing OLEDs. Finally, emission originating from a single ligand will yield greater color purity, as mixing of emissions from multiple delocalized excited states will not be possible. Together, these properties should allow for the rational tuning of other ancillary ligands without electronic interference from **bc**.

## Results and discussion

**Fig. 2:**
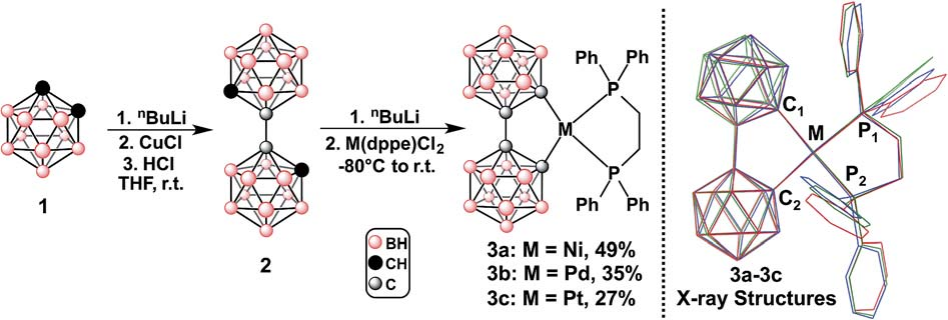
Left: Synthesis of M(bc)(dppe), where M = Ni (**3a**), Pd (**3b**), Pt (**3c**). The synthesis of **2** was adapted from ref. 10*j*. Right: Stacked X-ray crystal structures of compounds **3a–3c** (CCDC ; 1446940–1446942), illustrating structural similarity down the group. Red = **3a**, green = **3b**, blue = **3c** (see ESI† for thermal ellipsoid plots of **3a–3c**).

To test our hypothesis, we first proceeded to evaluate the photophysical behavior of **bc** as a ligand in a series of d^8^ model transition metal complexes of the type M(bc)(dppe) (M = Ni(ii), Pd(ii), Pt(ii); dppe = 1,2-bis(diphenylphosphino)ethane; Fig. 2). The dilithio salt of **bc** (Li_2_[**bc**]) was generated in THF and transferred into a slurry of M(dppe)Cl_2_ in THF at –80 °C. The reaction was allowed to slowly warm to room temperature overnight, yielding a dark brown solution. Pure compounds were isolated in the yields reported in Fig. 2 as crystalline yellow-orange solids after purification using column chromatography on silica or alumina (see ESI†). All reactions can be easily monitored *via*^31^P NMR spectroscopy. For example, **3a** and **3b** exhibit a significant (>10 ppm) upfield shift in their observed ^31^P NMR singlet resonance compared to the starting metal-based precursors (see ESI†). These results are also consistent with the recent work by Welch and co-workers who independently synthesized **3a**.11*a* Observed ^1^*J*_Pt–P_ coupling values for **3c** change significantly compared to the starting material, exhibiting a decrease of 1133 Hz (^1^*J*_Pt–P_ value changes from 3624 Hz to 2491 Hz). The substantial reduction in the magnitude of the ^1^*J*_Pt–P_ coupling for **3c** can be attributed to the strong ligand field of **bc***versus* that of the chloride ligands.^13^

To demonstrate that the **bc** framework is structurally similar to the biphenyl (bph) framework, we compared bond distances, angles, and molecular geometries of **3a–3c** to those of a series of cyclometalated M(bph)(L^L) in reported X-ray crystal structures, and determined that **bc** does not influence the intramolecular geometry much differently than the bph ligand. Furthermore, the M–P bond lengths in **3a–3c** are also consistent with the strong-field ligand nature of **bc** in these complexes (see ESI† for specific discussion).

UV-Vis spectroscopic measurements were performed on **3a–3c** and revealed strong transitions in the UV region below 360 nm corresponding to π–π* transitions on the ligand. UV-Vis spectra of **3a–3c** also feature weaker intensity transitions in the visible region ranging from 400–500 nm that result from M(ii)-(dppe) MLCT. DFT calculations were performed on the geometry optimized X-ray crystal structure inputs of **3a–3c** in order to confirm the nature of the observed electronic transitions (see ESI†). Our computational studies reveal an almost entirely metal-based HOMO and dppe ligand-based LUMO with negligible contribution from the **bc** ligand in all frontier orbitals. As we hypothesized previously, **bc** chelated to the d^8^ transition metals in our model complexes remains uninvolved in all MLCT-based visible transitions, suggesting its photophysical innocence in the UV-Vis region.

**Fig. 3:**
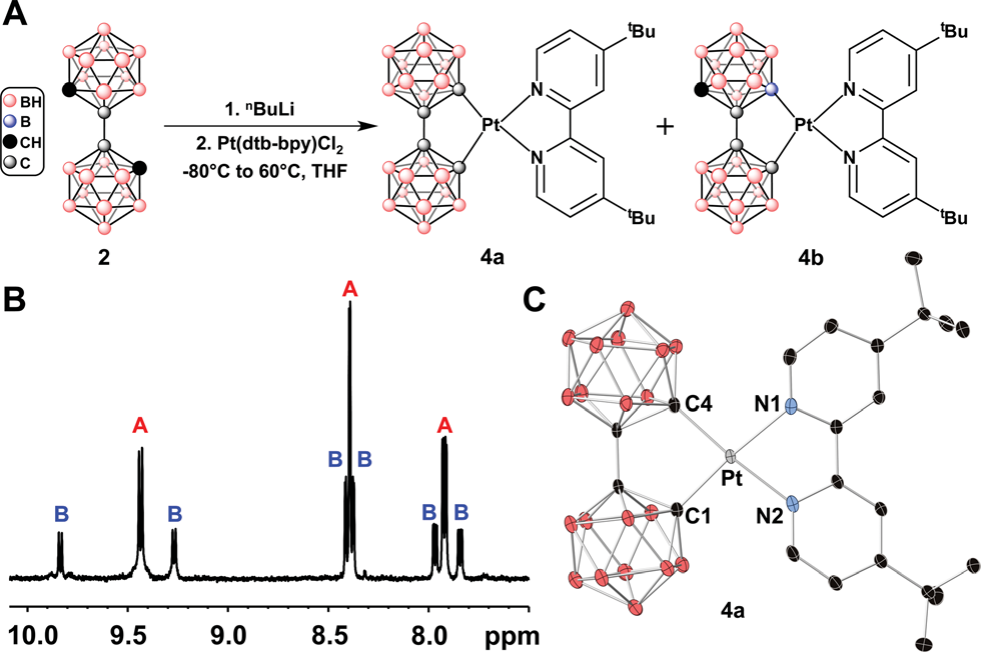
(A) Synthesis of the Pt(bc)(dtb-bpy) complex leads to a mixture containing two product isomers featuring a κ^2^-C,C-bound **bc** (**4a**) and κ^2^-B,C-bound **bc** (**4b**). (B) ^1^H NMR spectrum of the aryl region for the isolated mixture of **4a** (label A) and **4b** (label B). (C) Single crystal X-ray structure of **4a** drawn with 50% thermal ellipsoid probability (CCDC ; 1446943). H atoms are omitted for clarity.

Next, we sought to synthesize a Pt(ii)-bpy (bpy = 2,2′-bipyridine) complex chelated by **bc**, as Pt(ii) complexes containing this class of ligands are known to exhibit phosphorescent emission.12a–c Starting with the addition of Li_2_[**bc**] to a slurry of Pt(bpy)Cl_2_, a large amount of emissive, insoluble product was obtained. The extremely low solubility of this product in common organic solvents hampered its characterization. In order to potentially circumvent this issue, we then chose the 4,4′-di-*tert*-butyl-2,2′-bipyridine (dtb-bpy) ligand as an alternative, anticipating more favorable solubility properties. Using the same synthetic route yielded, again, a largely insoluble, emissive solid (Fig. 3A). After dissolving the crude product in hot 1,2-difluorobenzene and passing the solution through a Celite plug, a non-emissive solid was left on the Celite; a yellow solid that emitted blue-green under UV excitation (365 nm) remained after all volatiles were removed *in vacuo*. Surprisingly, ^1^H NMR spectroscopic data suggests that the isolated product consists of a mixture of two species (**4a**/**4b**) with a **bc** ligand chelated to the Pt(ii) center in both κ^2^-C,C-bound (**4a**) and κ^2^-B,C-bound modes (**4b**) (Fig. 3). The κ^2^-C,C-bound species **4a**, derived from the symmetric binding of the **bc** ligand, is consistent with the presence of three resonances of equal integration in the aromatic region (dtb-bpy ligand) of the ^1^H NMR spectrum (Fig. 3B, label A). The six remaining resonances in the aromatic region (Fig. 3B, label B) of the ^1^H NMR spectrum are consistent with an asymmetric κ^2^-B,C-bound **bc** species **4b** (*vide infra*). From the relative integration of these two sets of resonances, we estimate that the produced mixture contained a ratio of 1.4 : 1.0 of **4a** to **4b**. Repeated attempts to optimize this reaction produced the same mixture in varying ratios of **4a** and **4b** (see ESI†). Attempts to drive the formation of one isomer from the mixture of isomers while heating under forcing conditions produced no observable change in both ^1^H and ^11^B NMR spectra. Notably, during the preparation of this manuscript, Welch and co-workers reported the synthesis of a series of Ru(ii) complexes chelated by the κ^2^-B,C-bound **bc** ligand.11*c* The authors explained this B–Ru bond formation results from a competitive B–H activation process, which we think is reminiscent of the observed formation of **4b** in this work.

A mixture of **4a** and **4b** was dissolved in hot 2-MeTHF and allowed to cool to room temperature, yielding single crystals of **4a** (Fig. 3C). The molecule adopts a minimally distorted square planar geometry with the C–Pt–N_trans_ angles at 174.2(7)° and 173.0(8)°. The bulky **bc** ligand forces the molecule to pack “head-to-tail” with Pt(ii)···Pt(ii) distances ranging between 5.87(0) Å and 5.52(0) Å (see ESI†), which far exceed the reported 3.15–3.76 Å expected for intermolecular Pt(ii)···Pt(ii) interactions^2^,^3^ (see ESI† for further discussion about the single crystal X-ray structure of **4a**).

**Fig. 4:**
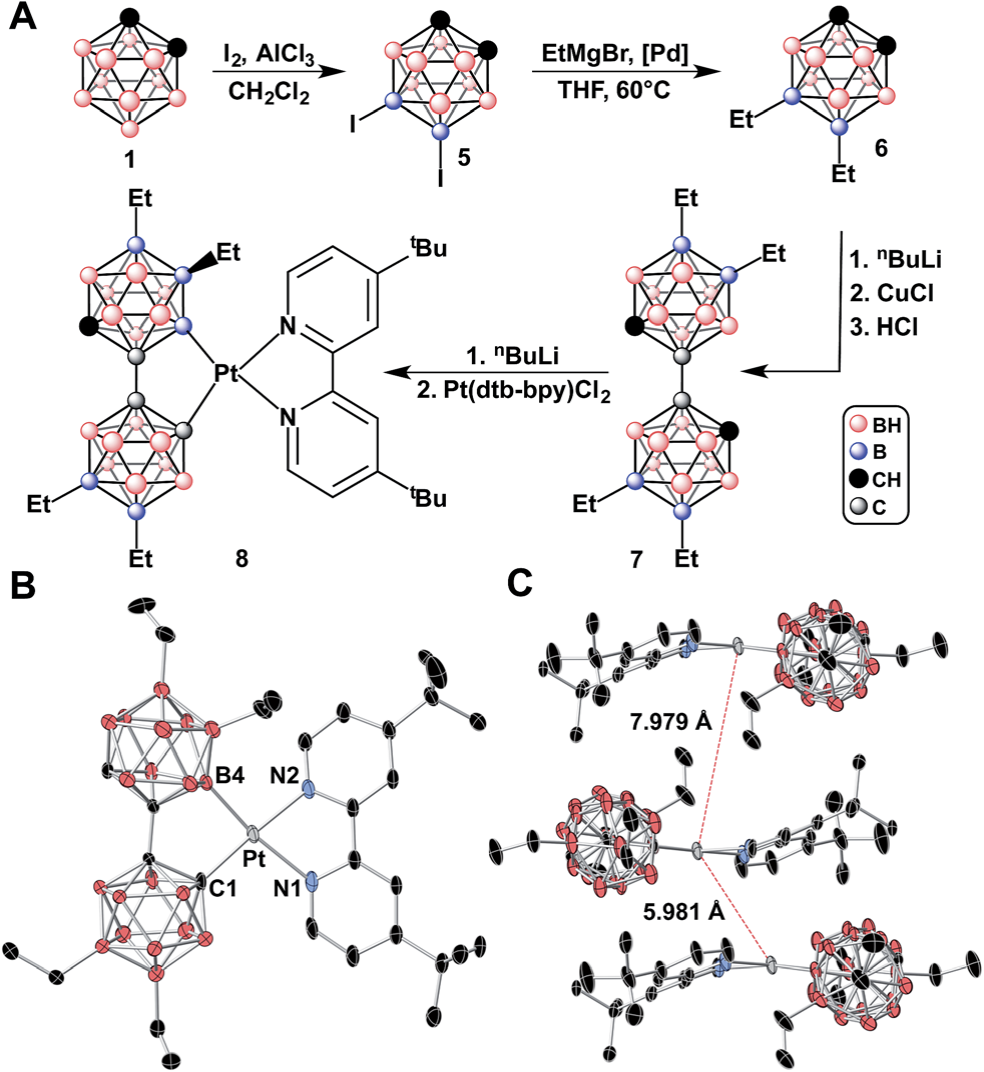
(A) Synthetic route to 9,9′,12,12′-tetraethyl-1,1′-bis(*o*-carborane) (**7**), syntheses of **5** and **6** from ref. 6*k*. (B) X-ray crystal structure of **8** (CCDC ; 1446944) with thermal ellipsoids drawn at 50% probability, H atoms omitted for clarity. (C) Stacking of **8** with Pt(ii)···Pt(ii) distances of 5.981 Å and 7.979 Å.

Given that the **bc** framework is amenable to substitution, we hypothesized that functionalizing this scaffold with alkyl groups would increase the solubility of the resulting Pt(ii) complexes, ultimately allowing us to better characterize these emissive species. We therefore installed ethyl groups at the B(9) and B(12) positions of the parent 9,12-B-diiodo-*o*-carborane (**5**) using Kumada cross-coupling conditions producing bis(alkylated) species **6** (Fig. 4A and ESI†). Compound **6** was then subjected to Cu-mediated oxidative coupling conditions, ultimately producing the tetralkylated-**bc** (**7**) in 50% isolated yield (Fig. 4A and ESI†).10*j* Compound **7** was dilithiated and added to Pt(dtb-bpy)Cl_2_ in a similar manner as with **2** (Fig. 4A). Surprisingly, after the reaction mixture was stirred for a day at 60 °C, predominantly a κ^2^-B,C-bound isomer **8** was observed by ^1^H and 2D ^13^C–^1^H HSQC NMR spectroscopy (>80%). Purification of the resulting mixture further afforded pure κ^2^-B,C-bound species as a pale orange solid which exhibits blue-green emission in the solid state and, as hypothesized, is extremely soluble in the majority of common organic solvents. To our knowledge, this is the first reported example of a functionalized **bc** bound to a metal.10*i*

Crystals of **8** suitable for X-ray analysis were grown by slow evaporation of diethyl ether over the course of one week. The diffraction study confirmed the presence of the asymmetric isomer with one Pt–C(1) bond and one Pt–B(4) bond (Fig. 4B). At 2.07(3) Å, the Pt–B(4) bond is slightly longer than the 2.03(6) Å Pt–C(1) bond. Furthermore, the greater *trans* influence of the carborane-based boryl moiety^14^ can be seen in the elongation of the Pt–N bond lengths: the Pt–N(1) bond is 2.17(5) Å, whereas the Pt–N(2) bond is only 2.05(3) Å. As a result of the asymmetric binding of the **bc**-based ligand in **8**, one carborane cage rotates and forces the ethyl group about 30° out of the plane created by C(1)–Pt–B(4), whereas the other 3 ethyl groups sit in the square plane (Fig. 4C). This protruding ethyl group likely forces the dtb-bpy out of the square plane, causing the molecule to adopt a slightly distorted square planar structure; however, bond angles of 176.5(5)° for C(1)–Pt–N(2) and 168.8(2)° for B(4)–Pt–N(1) are well within the range of corresponding angles in previously reported 4-coordinate Pt(bph)(N^N) compounds (see ESI†).

Importantly, the intermolecular Pt(ii)···Pt(ii) distances were augmented even more in the solid-state than in **4a** through the introduction of ethyl groups, yielding Pt(ii)···Pt(ii) distances of 5.891 Å (when ethyl groups face away from each other) and 7.979 Å (when ethyl groups point towards each other), effectively preventing any potential intermolecular Pt(ii)···Pt(ii) interactions (Fig. 4C). Furthermore, the solid-state packing adopts a “head-to-tail” arrangement such that dtb-bpy lies above and below the **bc**-based ligand in the crystal lattice, eliminating the potential for any π–π stacking interactions, which have also been reported to result in deleterious non-radiative emission quenching.2c,3d

**Fig. 5:**
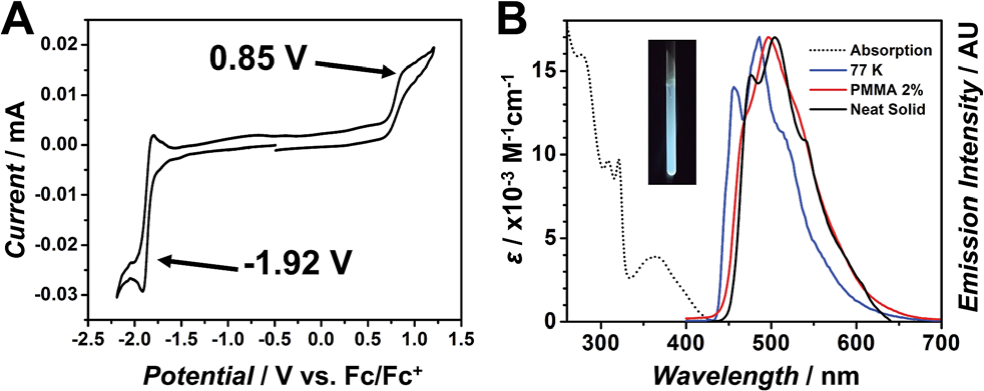
(A) Cyclic voltammogram of **8***versus* Fc/Fc^+^ in MeCN with a glassy carbon working electrode, scan rate = 0.1 V s^–1^. (B) UV-Vis absorption spectrum in CH_2_Cl_2_ (dotted line) and emission spectra of **8** in 2-MeTHF at 77 K (blue), 2 wt% PMMA film (red), and neat solid (black), *λ*_exc_ = 380 nm; inset: picture of **8** in 2-MeTHF at 77 K (*λ*_exc_ = 365 nm).

Cyclic voltammetry (CV) of **8** reveals a reversible, one-electron reduction (*E*Red1/2 = –1.92 V) and an irreversible one-electron oxidation (*E*Ox1/2 = 0.85 V), as shown in Table 1 and Fig. 5A. This electrochemical behavior is consistent with other square planar Pt(ii) species undergoing a reversible ligand-centered reduction and irreversible metal-centered oxidation.^2^–^4^,12a,c,15a Further, DFT calculations support these data (*vide infra*).

**Table 1:** Electrochemical data for **8** and related compounds from literature^*a*^

Compound	*E* Red 1/2 (V)	*E* Ox 1/2 (V)	Solvent	Reference
**8**	–1.92^*c*^	0.85^*d*^	MeCN	This work
Pt(bph)(bpy)^*b*^	–1.87^*c*^	–0.33^*d*^	MeCN	12*a*
Pt(bph)(en)^*b*^	–2.13^*c*^	0.25^*d*^	CH_2_Cl_2_	12*c*

^*a*^Values reported relative to the ferrocene/ferrocenium couple (Fc/Fc^+^).

^*b*^Values were corrected according to ref. 15*b*.

^*c*^Reversible.

^*d*^Irreversible.

Though electrochemical characterization for heteroleptic Pt(ii) complexes bound by a dianionic bidentate ligand and a neutral bidentate ligand are scarce, Table 1 presents redox potentials for two such examples, Pt(bph)(bpy) and Pt(bph)(en) (where en = 1,2-ethylenediamine).15*b* The reduction potential for **8** is similar to the other two compounds (Table 1, column 2); however, the oxidation potential of **8** is significantly greater (Table 1, column 3). This is consistent with the strong field ligand character of the **bc**-based framework, which should make it more difficult to remove an electron from the Pt(ii) HOMO level in **8**.

Similar to **4a**/**4b**, we observed that **8** emits an intense blue-green color upon irradiation with a table-top UV lamp at 365 nm at room temperature. Given the improved solubility properties of **8**, we were able to carry out a detailed series of photophysical measurements in order to ascertain the efficiency and nature of this luminescent behaviour. The UV-Vis and phosphorescent emission spectra for **8** are presented in Fig. 5B with corresponding data in Table 2. The absorption spectrum reveals strong transitions in the UV region (≤330 nm) that arise from π–π* transitions on the dtb-bpy ligand. The broad, lower intensity band from 340–420 nm can be assigned to both singlet and triplet metal-to-ligand charge transfers (^1^MLCT and ^3^MLCT). Compound **8** is non-emissive in solution at room temperature, suggesting emission might be thermally quenched through interaction with solvent molecules. However, at 77 K in 2-MeTHF, bright blue phosphorescence is observed (*λ*_max_ = 485 nm, *τ* = 11.4 μs). The well-defined vibrational features suggest ligand-centered emission resulting from an MLCT.1*a*

**Table 2:** Photophysical data of **8** from solution-based^*a*^ and solid-state^*b*^ measurements

Medium	Em. *λ*_max_ (nm)	*φ* ^*c*^	*τ* ^*d*^ (μs)	*k*_r_ ^*f*^ (10^4^ s^–1^)	*k*_nr_ ^*f*^ (10^4^ s^–1^)
77 K	456, 486, 514	—	11.4	—	—
PMMA film	497	0.07	4.24^*e*^	1.67	22.1
Neat solid	476, 505, 540	0.03	0.94^*e*^	3.20	103.1

^*a*^Solutions at room temperature were non-emissive, and 77 K spectra were measured in 2-MeTHF.

^*b*^PMMA film was prepared as 2 wt%, neat solid was **8** in powder form.

^*c*^Quantum yields were measured using an integrating sphere under N_2_.

^*d*^77 K lifetime was measured in 2-MeTHF, PMMA film and neat solid lifetimes were measured in the absence of air.

^*e*^Values obtained from the weighted average of a multi-exponential decay.

^*f*^Calculated according to the equations *k*_r_ = *φ*/*τ* and *k*_nr_ = (1 – *φ*)/*τ*, where *k*_r_ is the radiative rate constant, *k*_nr_ is the non-radiative rate constant, *φ* is the quantum yield, and *τ* is the luminescence lifetime.

Similarly, the neat solid **8** also exhibits an emission profile with a resolved vibronic fine structure, further suggesting the ligand-centered emission. Compared to the emission profile from the neat solid, emission from the solution at 77 K is hypsochromically shifted by roughly 20 nm. This shift is expected as vibrational relaxations to a lower energy excited state will not be favorable at lower temperatures, resulting in a higher energy, blue-shifted emission observed for **8** at 77 K in 2-MeTHF. When **8** is doped in a PMMA matrix (2 wt%), the emission profile is broadened and the peak is blue-shifted by about 8 nm *versus* the emission of the neat solid. The excited-state lifetime (*τ*) for **8** increases as the environment becomes more rigid. This increase is significant, going from 0.94 μs as a neat solid, to 4.24 μs doped in PMMA, further to 11.4 μs at 77 K. This evidence suggests that decreasing vibrational motion through a more rigid and ordered surrounding environment can preserve the excited state, possibly by minimizing the energy loss *via* non-radiative relaxation pathways.1*a*

From the measured excited state lifetimes and quantum yields for **8** doped in the PMMA matrix and neat solid, the radiative rate constant (*k*_r_) and non-radiative rate constant (*k*_nr_) could be calculated (Table 2). Though *k*_r_ for the doped PMMA film is half that of the neat solid, *k*_nr_ for the doped PMMA film decreased by about a factor of 5, which supports the trend seen for measured *τ* values (*vide supra*). Additionally, the doped PMMA film exhibits a quantum yield (*φ*) more than twice that of the neat solid, as well as a lifetime that is about 4.5 times greater. Based on these data, it is likely that the PMMA film decreases access to a non-radiative decay pathway through its behavior as a rigid matrix.

**Fig. 6:**
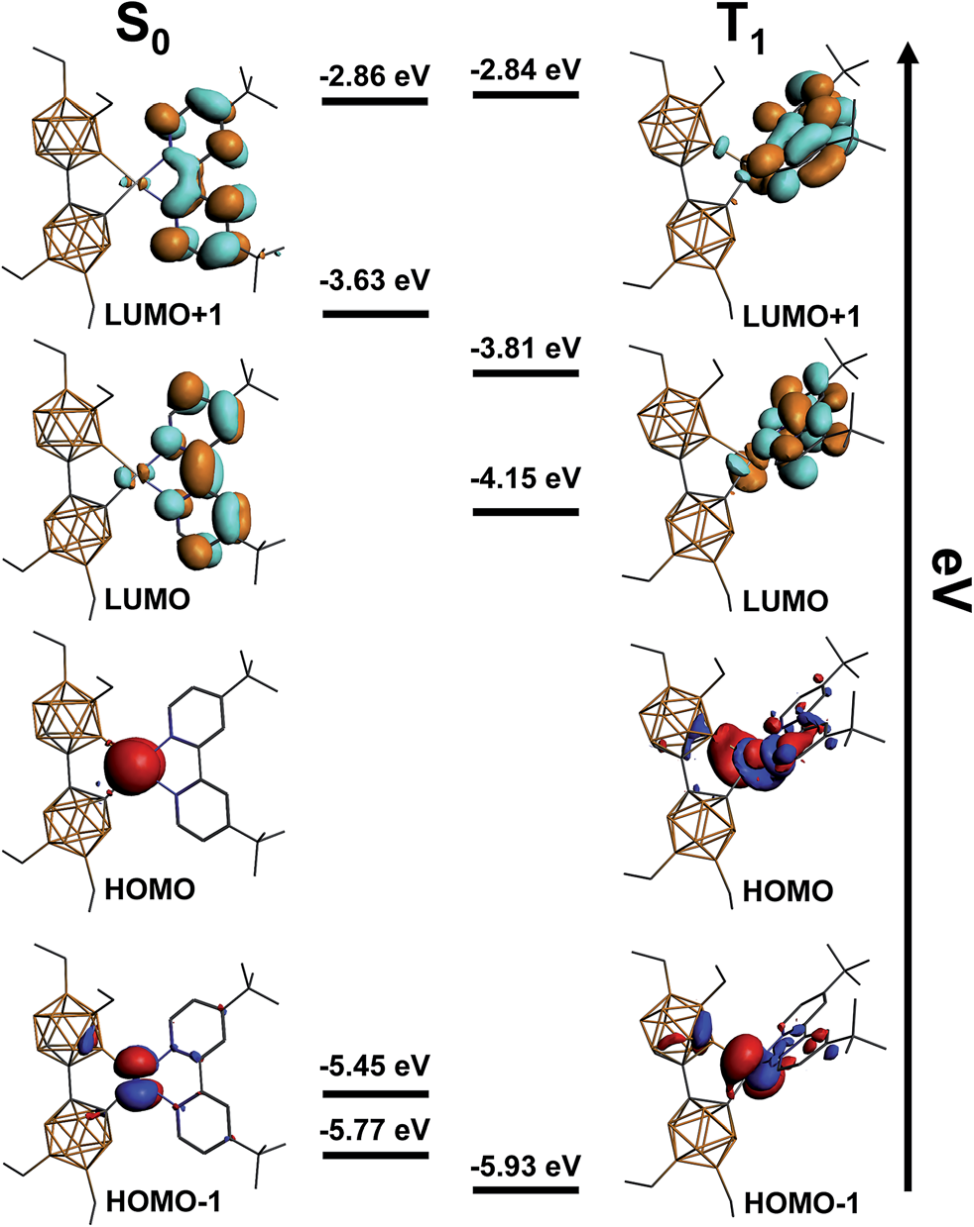
Frontier orbitals of **8** based on optimized geometries of S_0_ and T_1_ states (BP86-D3, TZP).

To further our understanding of the photophysical properties of **8**, we performed a DFT computational study at the BP86-D3 level using the TZP basis set (Fig. 6). The optimized geometry of the singlet state displays a slightly distorted square planar structure, which is in agreement with the obtained single crystal X-ray structure. The frontier orbital diagram indicates a HOMO and HOMO–1 almost completely localized on the Pt(ii) with negligible contribution from the **bc** fragment. Both the LUMO and LUMO+1 are isolated on dtb-bpy, which corroborates the observed ligand-centered phosphorescence of **8** without observed contribution from the **bc** fragment. The optimized geometry of the triplet state, however, reveals an almost tetrahedral structure that is extremely distorted from the favorable square planar geometry seen in the ground state (Fig. 6). In the excited state, the complex twists *via* a non-radiative decay pathway, resulting in a large value for *k*_nr_. This hypothesis supports the observed decrease in *k*_nr_ from the pure solid to the PMMA matrix: as the rigidity of the environment increases, the geometry of the molecule will be more difficult to distort. These calculations suggest that future molecular designs should incorporate a large degree of steric bulk to potentially minimize this excited state distortion, thereby improving phosphorescence efficiency in these compounds.

In general, phosphorescent blue OLEDs suffer from short lifetimes (∼600 hours) relative to their red and green counterparts (10^6^ hours).16*a* Several groups have previously investigated the degradation of blue OLEDs and found that the decomposition of the phosphorescent dopant molecules occurs during regular use, greatly inhibiting the overall lifetime and efficiency of the device.16b–e Thermogravimetric analysis of **8** suggests the **bc** ligand framework remains intact upon heating to 500 °C (see ESI†). This observation suggests that chelating boron cluster scaffolds may be potentially appropriate ligands that can ameliorate previously described stability issues in OLED devices.

## Conclusions

In conclusion, for the first time, we have presented a detailed study on the behavior of 1,1′-bis(*o*-carborane) as a dianionic ligand (**bc**) in group 10 metal complexes. From the synthesis and characterization of model complexes, we have discovered that unlike the structurally reminiscent biphenyl ligand, **bc** displays a unique photophysical innocence and remains uninvolved in relevant photophysical transitions when bound to the group 10 transition metals. Furthermore, since **bc** introduces sufficient steric bulk above and below the square plane of the metal center, it effectively shuts down undesired intermolecular interactions in the solid-state. For Pt(ii) complexes featuring phosphorescent emission properties, this unique ligand design aspect allowed us to remove any possible Pt(ii)···Pt(ii) interactions, which commonly lead to luminescence quenching. The organomimetic6e,^17^ properties of carboranes in general, and **bc** in particular, enabled us to rationally tune the processability of a blue phosphorescent emitting Pt(ii) species. We are currently investigating the substitution of carborane-based ligands with bulkier functional groups that will minimize the excited state distortion. This work opens a new avenue in designing luminescent materials with improved properties incorporating robust and photophysically innocent multidentate ligand platforms.

## Supplementary Material

Supplementary information

Crystal structure data
