# Supplementary material for: Luminescent metal complexes featuring photophysically innocent boron cluster ligands
Source: Chem Sci. 2016 Apr 27;7(8):5132–8. doi: 10.1039/c6sc01146b (PMC5300762; doi:10.1039/c6sc01146b)
Supplement: Supplementary file 1 [file SC-007-C6SC01146B-s001.pdf]

Electronic Supplementary Material (ESI) for Chemical Science.
This journal is © The Royal Society of Chemistry 2016

Luminescent Metal Complexes Featuring Photophysically Innocent Boron Cluster Ligands — SI

Kent O. Kirlikovali, Jonathan C. Axtell, Alejandra Gonzalez, Alice C. Phung, Saeed I. Khan, and Alexander M. Spokoyny*

Department of Chemistry and Biochemistry, University of California, Los Angeles

607 Charles E. Young Drive East, Los Angeles, California 90095-1569

*E-mail: spokoyny@chem.ucla.edu

Supporting Information (SI)

Table of Contents

General Considerations	3
Materials	3
Instruments	3
Determination of molar extinction coefficients	4
Preparation of PMMA films	4
X-ray data collection and processing parameters	4
Cyclic Voltammetry	4
DFT Calculations	5
Experimental	5
UV-Vis Spectra of *o*-carborane (**1**) and 1,1′-bis-(*o*-carborane) (**2**)	16
Molecular structures of compounds mentioned in manuscript	16
Molecular structures of Ir(III)-based complexes containing carboranyl functional groups	17
Single crystal X-ray structures and data of **3a**-**3c**	17
Analysis of X-ray data for **3a**-**3c**	18
Scheme for oxidation of **3c**	21
Scheme for formation of side products during the synthesis of **3a**-**3c**	22
UV-Vis spectra of **3a**-**3c**	23
DFT analysis for **3a**-**3c**	24
Single crystal X-ray structure and solid-state stacking of **4a**	24
Analysis of X-ray data for **4a**	25
Single crystal X-ray structure for **7**	26
Single crystal X-ray structure and data for **8**	27
Thermogravimetric analysis (TGA) of **8**	28
Coordinates for geometry optimized structures of **3a**-**3c**, **8**	28
References	42-43

General considerations. All experiments were performed air and moisture free under an atmosphere of nitrogen using standard Schlenk and cannula techniques. THF used for reactions and MeCN used for cyclic voltammetry was purified and dried using a Grubbs column.[1] All post-Schlenk work-up and characterization was performed under ambient conditions. The "ambient conditions" for this manuscript refer to room temperature (20 - 25 °C) and uncontrolled laboratory air. Thin-layer chromatography (TLC) samples for carborane-containing compounds were stained with 1 wt. % $PdCl_2$ in 6M HCl and were developed with heat. Elemental analyses were carried out by Atlantic Microlab, Inc. in Norcross, GA.

Materials. Deuterated solvents were purchased from Cambridge Isotope Laboratories and used as received. MilliQ water described in this manuscript refers to purified potable water with a resistivity at 25 °C of ≤18.2 MΩ·cm. $Pd(cod)Cl_2$[2] and $Pt(cod)Cl_2$[3] were made according to literature procedures. *O*-carborane (1,2-$C_2B_{10}H_{12}$) was purchased from Boron Specialties (USA). $K_2[PtCl_6]$, $PdCl_2$, Ni(dppe)Cl_2, 1,2-bis(diphenylphosphino)ethane (dppe), 2,2′-bipyridine (bpy), 4,4′-di-*tert*-butyl-2,2′-bipyridine (dtb-bpy), 1,5-cyclooctadiene (cod), iodine, ethyl magnesium bromide, $Pd(PPh_3)_2Cl_2$, and tetrabutylammonium hexafluorophosphate (≥99.0%, electrochemical grade) were purchased from Sigma-Aldrich. Glass-backed Silica Gel 60 GLA TLC plates were purchased from Fisher Scientific. Aluminum oxide (activated, basic, Brockmann Grade I, 58 Å, 60 mesh powder) was purchased from Alfa Aesar. All reagents were used as received unless otherwise indicated.

Instruments. ^1H NMR spectra were obtained on a Bruker AV500 or a Bruker AV400 spectrometer; $^{13}C\{^1H\}$ NMR spectra were obtained on a Bruker AV500 spectrometer; ^{11}B and ^{31}P NMR spectra were obtained on a Bruker DRX500 spectrometer. Bruker Topspin software was used to process the NMR data. ^1H and $^{13}C\{^1H\}$ NMR spectra were referenced to residual solvent resonances in deuterated solvents (CD_2Cl_2: ^1H, 5.320 ppm; ^{13}C, 53.840 ppm; $CDCl_3$: ^1H, 7.260 ppm; ^{13}C, 73.840 ppm; THF-d^8: ^1H, 5.020 ppm; ^{13}C, 61.500 ppm; Note: due to high humidity H_2O resonances are often present). ^{11}B and ^{31}P and NMR spectra were referenced to $BF_3 \cdot Et_2O$ (0 ppm) and H_3PO_4 (0 ppm) standards, respectively. Mass spectrometry data was acquired using a Thermo Instruments Exactive Plus with IonSense ID-CUBE DART source instrument (compound **4a** and **4b**), and a Thermo Scientific™ Q-Exactive™ Plus instrument with a quadrupole mass filter and Orbitrap mass analyzer (compound **8**). UV−Vis spectra were

recorded on a Hewlett-Packard 4853 diode array spectrometer. Phosphorescence lifetime measurements for **8** were performed by a time-correlated single-photon counting method using an IBH fluorocube lifetime instrument equipped with a 405 nm LED excitation source. Quantum yield measurements were carried out using a Hamamatsu C9920 system equipped with a xenon lamp, calibrated integrating sphere, and model C10027 photonic multichannel analyzer. Steady-state emission measurements of **8** as a solid, in the thin film, and in solution at 77 K were performed using a Photon Technology International QuantaMaster spectrofluorimeter.

Preparation of PMMA thin film: A solution of PMMA (0.085 g, 120 kDa) in toluene (1.5 mL) was thermally sonicated for 3 hours at 40°C, or until PMMA was completely dissolved. Next, **8** (0.0017 mg, 2 wt. %) was added to the solution and sonicated for 5 minutes, yielding an orange solution. The solution of **8**/PMMA was spin coated on a quartz substrate (3-5 drops, 800-1,000 RPM, 30 seconds), and this was repeated until the film was thick enough emit light under λ = 365 nm excitation in a dark room under ambient conditions.

Determination of molar extinction coefficients: Extinction coefficients were determined through a series of 5 dilutions with a maximum absorption between 0.1 and 0.7.

X-ray data collection and processing parameters. For **3a-3c**, **4a**, and **8** a single crystal was mounted on a nylon loop using perfluoropolyether oil and cooled rapidly to 100 K with a stream of cold dinitrogen. Diffraction data were measured using a Bruker APEX-II CCD diffractometer using Mo-K_α radiation. The cell refinement and data reduction were carried out using Bruker SAINT and the structure was solved with SHELXS-97. All subsequent crystallographic calculations were performed using SHELXL-2013.

Cyclic voltammetry. Cyclic voltammetry was performed on **8** and using a CH Instruments Model 600D potentiostat with a glassy carbon disc working electrode, platinum wire counter electrode, and Ag/AgCl wire reference in a saturated solution of KCl in MeCN. All experiments were conducted in 0.1M $[N^{n}Bu_4]PF_6$/MeCN with 0.5 mM analyte concentrations. MeCN solutions were degassed by sparging with argon for 10 minutes, and the cyclic voltammetry was performed under constant flow of argon gas. A scan rate of 0.1 mV/s was used with ferrocene as

an internal standard.

DFT Calculations. All optimized geometries were calculated from crystallographic data and optimized with DFT calculations using standard triple-ξ polarization (TZP) basis sets available in the Amsterdam Density Functional 2014.04 Rev. 44409 (ADF)[4] software suite, with Becke[5] and Perdew[6] (BP) Slater-type orbitals (STOs) on a 6 core Apple computer. The local density approximation (LDA) was made with BP and exchange and correlation corrections available by default in the ADF 2014.04 suite. Single point calculations were performed using BP86 level of theory with the Grimme D3 dispersion correction[7] and a TZP basis set. Electron cores were frozen to 2p for Ni, Pd, and Pt; 1s for B, C, and N; and 2s for P atoms. Electron spins were restricted for S_0 calculations, and electron spins were unrestricted for T_1 calculations. Relativistic correlations were made using Zero-Order Relativistic Approximation (ZORA) for Pt (**3c** and **8**).

Experimental

1,1′-Bis(*o*-carborane) [2].

O-carborane (5.00 g, 34.67 mmol) was added to a 500mL Schlenk flask, which was then evacuated and backfilled with N_2 three times. Dry toluene (200 mL) was transferred *via* cannula, and the contents were stirred to give a slightly cloudy white solution. Next, ^{n}BuLi (28.4 mL, 71.0 mmol, 2.5 M in hexane) was added slowly *via* syringe. The solution immediately turned milky white and the mixture was allowed to stir at room temperature for 21 hours. CuCl powder (9.44 g, 95.3 mmol) was then added, causing the reaction mixture to immediately turn gray and then dark red over the course of 30 minutes. This suspension was stirred for 3 days, after which toluene was removed by rotary evaporation. CH_2Cl_2 (50 mL) and aqueous HCl (120 mL, 12 M) were added and stirred vigorously for 3 hours, or until the color turned green (indicating the formation of CuCl). The organic layer was separated, and the aqueous layer was washed with CH_2Cl_2 (3 x 100mL). The combined organic fractions were dried over Na_2SO_4, filtered, and dried *in vacuo*, yielding a light brown solid (4.60 g). This crude product was purified by stepwise vacuum sublimation: sublimation under dynamic vacuum (0.20 Torr) at 75°C for 1 hour yielded crystals of unreacted *o*-carborane. After removal, the remaining solid was heated to 180˚C under dynamic vacuum (0.20 Torr) for 1-2 hours. Pure product in the form of white crystals was

isolated from the cold finger and dried under vacuum overnight (3.61 g, 73%). **^{1}H NMR** (CD_2Cl_2, 500 MHz): δ 3.90 (s, 2H, CH_{cage}), 3.40-1.40 (br. m, 18H, BH); **^{11}B NMR** (CD_2Cl_2, 160 MHz): 2.38 (4B), -8.98 (2B), -10.00 (6B), -11.17 (4B), 12.24 (2B), -13.29 (2B); **^{13}C NMR** (CD_2Cl_2, 125 MHz): 72.31, 63.25. Synthesis adapted from Reference 8.

9,12-diiodo-o-carborane [5].

To a 200 mL Schlenk flask was added *o*-carborane (2.88 g, 20.0 mmol) and one equivalent I_2 (5.08 g, 20.0 mmol). The flask was evacuated and backfilled with N_2 at least three times to remove residual moisture. Dry CH_2Cl_2 (100 mL) was transferred to the reaction vessel *via* cannula and the contents of the flask were stirred to give a purple solution. Next, $AlCl_3$ (0.533 g, 4.0 mmol) was added under positive N_2 flow, and the reaction was refluxed until the color fades to pale yellow (~2-3 hours). A second equivalent of I_2 (5.08 g, 20.0 mmol) and $AlCl_3$ (0.267g, 2.0 mmol) were added, and the reaction was heated to 45°C and allowed to reflux overnight. The next day, the dark brown reaction mixture was diluted with H_2O (50 mL, or until effervescence subsided), and unreacted I_2 was quenched with Na_2SO_3 (0.708 g, 5.62 mmol). After stirring for 10 minutes, the purple organic layer was separated and the aqueous layer was washed with CH_2Cl_2 (40 mL x 3) and dried over $MgSO_4$. If the solution is still colored, activated charcoal can be added. This solution was passed through a silica plug and eluted with CH_2Cl_2 to give a light gray solution. Solvent was then removed by rotary evaporation, yielding a lavender solid. The crude product was then sublimed under vacuum (150-160°C, 5-6 hours, 0.20 Torr) to afford a white solid (6.52 g, 82%) on the cold finger, which was isolated and dried under vacuum overnight before further use. Alternatively, the crude product can be purified by recrystallization by layering CH_2Cl_2 with an equal volume of hexane and storing at -20°C. After 3-4 hours, the white crystals can be filtered off and washed with cold hexane. **^{1}H NMR** ($CDCl_3$, 500 MHz) δ 3.99 (s, 2H, CH), 3.50-1.80 (m, 9H, BH); **^{11}B NMR** ($CDCl_3$, 160 MHz): -5.50 (d, 2B), -12.67 (d, 4B), -14.01 (s, 3B), -14.89 (s, 1B); **^{13}C** NMR ($CDCl_3$, ppm): 51.71. Synthesis adapted from Reference 9.

9,12-diethyl-o-carborane [6].

To a 50 mL Schlenk flask was added 9,12-diiodo-*o*-carborane (1.98 g, 5.00 mmol) and $PdCl_2(PPh_3)_2$ (0.07091 g, 0.10 mmol). The flask was left under vacuum for 30 minutes, then

backfilled with N_2. Dry THF (20 mL) was added *via* cannula, and the flask was stirred in an ice bath for 10 minutes. EtMgBr (7.9 mL, 3.0 M, 23.7 mmol) was then slowly added. The resulting mixture was gently refluxed for 10 days and monitored by GC-MS. The reaction was cooled to room temperature and quenched with H_2O, then charged with diethyl ether (150 mL). The organic layer was separated, and the aqueous layer was extracted with diethyl ether (3 x 80mL). Aqueous HCl (5%, 30 mL) was added to the organic layer, and the organic layer was separated and solvent removed *via* rotary evaporation. The crude product was purified *via* silica column chromatography (ethyl acetate:hexanes 1:9). Solvent was removed under vacuum to afford the desired product as a low-melting red solid (0.250g, 31%). **^{1}H NMR** (CD_2Cl_2, 500 MHz) δ 3.47 (2H, s, CH_{cage}), 3.00-1.20 (8H, br. m, BH), 0.89 (t, 6H, CH_3), 0.71 (q, 4H, CH_2); **^{11}B NMR** (CD_2Cl_2, 160 MHz): 9.64 (s, 2B), -8.43 (d, 2B), -14.24 (d, 4B), -16.21 (d, 2B); **^{13}C** NMR ($CDCl_3$, 125 MHz): 47.71, 13.52, 9.35 (br). Synthesis adapted from Reference 9.

9,9′,12,12′-Tetra-ethyl-1,1′-bis(*o*-carborane) [7].

To a clean 25 mL Schlenk flask, 9,12-diethyl-*o*-carborane (0.500 g, 2.50 mmol) was added, and the flask was cooled to -78°C. The flask was evacuated and backfilled with N_2 five times, followed by the addition of dry toluene (10 mL) *via* cannula, yielding a yellow solution. Next, ^{n}BuLi (2.1 mL, 5.25 mmol, 2.5 M in hexanes) was added *via* syringe. The mixture, which immediately turned clear and gradually acquired a yellow color, was allowed to warm and was stirred at room temperature for 24 hours. CuCl was added (0.680 g, 6.87 mmol), and the resulting dark green suspension was allowed to stir for 3 days. Toluene was then removed under reduced pressure, and CH_2Cl_2 (5 mL) and aqueous HCl (15 mL, 10 M) were added and stirred vigorously to quench the resulting dark green reaction mixture. The mixture was allowed to stir until the aqueous layer turned light green (~3h). The organic layer was separated, and the aqueous layer was washed with CH_2Cl_2 (3 x 15mL). The combined organic fractions were dried over Na_2SO_4, filtered, and concentrated to dryness yielding a yellow-orange solid. The crude product was eluted through a pad of silica with hexanes and concentrated to dryness, yielding a pale yellow solid (0.385 g). Minimal pentane (2-3 mL) was added to the solid and stored in the freezer (-20°C) for recrystallization. The next day, pentane was decanted yielding white crystals. The supernatant was stored in the freezer and decanted after four hours, and this process was repeated once more yielding pure white crystals (Total: 0.231 g, 46%). **^{1}H NMR** (CD_2Cl_2, 500

MHz) δ 3.72 (s, 2H, cage CH), 3.4-1.2 (br. m, 16H, BH), 0.86 (m, 12H, CH_3), 0.69 (m, 8H, CH_2); **^{11}B NMR** (CD_2Cl_2, 160 MHz): 10.04 (s, 4B), -8.58 (d, 4B), -12.09 (m, 12B); **^{13}C NMR** (CD_2Cl_2, 125 MHz): 65.48, 56.19, 13.32, 8.36. Single crystals for X-ray diffraction analysis were grown from a concentrated solution of pentane stored in a freezer at -15°C overnight.

Pd(dppe)Cl$_2$

Pd(cod)Cl_2 (0.286 g, 1.00 mmol) and CH_2Cl_2 (6 mL) were combined and stirred in a large *via*l to give a yellow slurry, and 1,2-bis(diphenylphosphino)ethane (dppe, 0.398 g, 1.00 mmol) was then added. The color quickly changed to a lighter shade of yellow. After 15 minutes, hexane (14 mL) was added, and the *via*l was capped and stored in the freezer (-20°C) for 4 hours. The solid was filtered and washed with pentane (5 mL x 3) yielding the pale yellow product (0.517 g, 90%). **^{1}H NMR** (CD_2Cl_2, 400 MHz): 7.87 (8H, m), 7.60 (4H, m), 7.53 (8H, m), 2.47 (4H, d, CH_2); **^{31}P NMR** (CD_2Cl_2, 121 MHz): 64.7. Synthesis adapted from Reference 10.

Pt(dppe)Cl$_2$

Pt(cod)Cl_2 (0.374 g, 1.00 mmol) and CH_2Cl_2 (6 mL) were combined and stirred in a large *via*l to give a white slurry, and 1,2-bis(diphenylphosphino)ethane (dppe, 0.398 g, 1.00 mmol) was then added. After 15 minutes, hexane (14 mL) was added, and the *via*l was capped and stored in the freezer (-20°C) for 4 hours. The solid was filtered and washed with pentane (5 mL x 3) yielding the white product (0.576 g, 87%). **^{1}H NMR** (CD_2Cl_2, 400 MHz): 7.86 (8H, m), 7.56 (4H, m), 7.52 (8H, m), 2.36 (4H, d, CH_2); **^{31}P NMR** (CD_2Cl_2, 121 MHz): 41.8 ($^1J_{Pt-P}$ = 3622 Hz). Synthesis adapted from the synthesis for **Pd(dppe)Cl$_2$** in Reference 10.

Pt(dtb-bpy)Cl$_2$

Pt(cod)Cl_2 (0.350 g, 0.935 mmol), 4,4′-ditertbutyl-2,2′-bipyridine (dtb-bpy, 0.251 g, 0.935 mmol), and 8 mL methanol were stirred in a sealed tube at 75°C for 22 hours. As the suspension warmed up, the color changed to yellow-orange and eventually turned bright yellow. The tube was cooled to room temperature in a water bath and the yellow solid was filtered. After washing with diethyl ether (3 mL x 5) to remove 1,5-cyclooctadiene, the yellow product was dried under vacuum overnight (0.458 g, 92%). **^{1}H NMR** (CD_2Cl_2, 500 MHz): 9.52 (2H, d), 7.91

(2H, d), 7.57 (2H, dd), 1.44 (18H, s, CH_3); **^{13}C NMR** (CD_2Cl_2, 126 MHz): 164.9, 157.2, 149.3, 124.8, 119.9, 36.2, 30.3. Synthesis adapted from Reference 11.

Ni(bc)(dppe) [3a].

1,1′-Bis-(*o*-carborane) (0.100 g, 0.349 mmol) was added to a 10 mL Schlenk flask, which was then evacuated and backfilled with N_2 three times. Dry THF (4 mL) was transferred to the flask *via* cannula to give a clear solution. While stirring at room temperature, ^{n}BuLi (0.45 mL, 1.6 M, 0.72 mmol) was added drop-wise *via* syringe, giving a dark yellow solution. The reaction was then heated at 60°C for 3 hours, and the color slowly turned to a lighter yellow. After 3 hours, the flask was allowed to cool to room temperature yielding a golden-yellow solution. To a separate 25 mL Schlenk flask was added Ni(dppe)Cl_2 (0.184 g, 0.349 mmol), and the flask was evacuated and backfilled with N_2 three times. Dry THF (20 mL) was then transferred *via* cannula to give a red-orange slurry. This flask was cooled to -78°C and stirred for 5 minutes. The dilithio-bis-carborane mixture was added dropwise (~1 mL/min) to the cold Ni(dppe)Cl_2 slurry, and the reaction was allowed to warm up to room temperature and react overnight, giving a deep orange solution the following day. The solvent was removed *via* rotary evaporation after 18 hours, and the orange solid was dissolved in 5-10 mL CH_2Cl_2 and passed through a pad of Celite. After removal of solvent, crude orange product (0.160 g) remained and was purified *via* column chromatography: a wide 60 mL filter frit was loaded halfway with silica in hexanes, and the crude product was dissolved in the minimum amount of 1:1 CH_2Cl_2:hexanes (R_f = 0.50) and loaded on the silica. Any unreacted bis-carborane (R_f = 0.90) can be removed as the initial clear/light yellow elution, and the desired product can be cleanly collected as the orange band that follows. The orange solution was concentrated to dryness to give a yellow solid (0.125 g, 49%) that was dried under vacuum overnight. **^{1}H NMR** (CD_2Cl_2, 500 MHz) δ 7.77 (br. t, 8H, Ar), 7.64 (t, 4H, Ar), 7.54 (t, 8H, Ar), 4.0-0.6 (br. m, 20H, BH), 2.0 (d, 4H, CH_2, $^2J_{PH}$ = 15.60 Hz); **^{11}B NMR** (CD_2Cl_2, 160 MHz) -1.96 (d, 2B), -3.83 (d, 2B), -8.35 (d, 12B), -10.62 (m, 4B); **^{13}C NMR** (CD_2Cl_2, 125 MHz) 134.62 (t, $^2J_{PC}$ = 5.2 Hz), 132.51, 129.46 (t, $^3J_{PC}$ = 5.2 Hz), 127.78 (m), 87.31 (dd, $^2J_{PC(trans)}$ = 91.8 Hz, $^2J_{PC(cis)}$ = 38.4 Hz), 84.70, 28.28 (t, $^1J_{PC}$ = 21.5 Hz); **^{31}P{^{1}H} NMR** (CD_2Cl_2, 202 MHz): 49.09. NMR chemical shifts comparable to those in Reference 12. Single crystals for X-ray diffraction analysis were grown from slow vapor

diffusion of diethyl ether into dichloromethane. Elemental analysis (including 0.5 CH_2Cl_2) found (calculated for $C_{30.5}H_{45}B_{20}ClNiP_2$): C, 46.40 (46.73); H, 5.89 (5.79).

Pd(bc)(dppe) [3b].

1,1′-Bis-(*o*-carborane) (0.100 g, 0.349 mmol) was added to a 10 mL Schlenk flask, which was then evacuated and backfilled with N_2 three times. Dry THF (4 mL) was transferred to the flask *via* cannula to give a clear solution. While stirring at room temperature, ^{n}BuLi (0.45 mL, 1.6 M, 0.72 mmol) was added dropwise *via* syringe, giving a dark yellow solution. The reaction mixture was then heated at 60°C for 3 hours, and the color slowly turned lighter yellow. After 3 hours, the flask was allowed to cool to room temperature yielding a golden-yellow solution. Pd(dppe)Cl_2 (0.202 g, 0.349 mmol) was added to a separate 25mL Schlenk flask, which was then evacuated and backfilled with N_2 three times. Dry THF (20 mL) was then transferred *via* cannula to give a white slurry. This flask was cooled to -78°C and stirred for 10 minutes. The dilithio-bis-carborane solution was added dropwise (~1 mL/min) to the cold Pd(dppe)Cl_2 slurry, and the reaction was allowed to warm up to room temperature and stir overnight, giving a deep orange mixture the following day. THF was removed *via* rotary evaporation after 20 hours, and the remaining orange solid was dissolved in 5-10 mL CH_2Cl_2 and passed through a pad of Celite. After removal of solvent *in vacuo*, crude orange product (0.225 g) remained and was purified *via* column chromatography. A wide 60 mL filter frit was loaded halfway with silica in hexanes, and the crude product was dissolved in minimal 1:1 CH_2Cl_2:hexanes (R_f = 0.50) and loaded on the silica. Any unreacted bis-carborane (R_f = 0.90) can be removed as the initial clear/light yellow elution, and the desired product can be cleanly collected as the orange band that follows. The orange solution was concentrated to dryness to give an orange solid (0.70 g, 25%) that was dried under vacuum overnight. **^{1}H NMR** (CD_2Cl_2, 500 MHz) δ 7.64 (t, 4H, Ar), 7.62 (t, 8H, Ar), 7.55 (t, 8H, Ar), 3.6-0.6 (br. m, 20H, BH), 2.12 (d, 4H, CH^2, $^{2}J_{PH}$ = 20.4 Hz); **^{11}B NMR** (CD_2Cl_2, 160 MHz) -3.20 (m, 4B), -8.74 (m, 16B); **^{13}C NMR** (CD_2Cl_2, 125 MHz) 134.57 (t, $^{2}J_{PC}$ = 5.6 Hz), 132.74, 129.64 (t, $^{3}J_{PC}$ = 5.4 Hz), 127.13, 91.90 (dd, $^{2}J_{PC(trans)}$ = 138.7 Hz, $^{2}J_{PC(cis)}$ = 14.7 Hz), 84.80, 28.82 (t, $^{1}J_{PC}$ = 22.3 Hz); **^{31}P{^{1}H} NMR** (CD_2Cl_2, 202 MHz) 51.63. Single crystals for X-ray diffraction analysis were grown by cooling a concentrated solution of hot benzene to room temperature. Elemental analysis found (calculated for $C_{30}H_{44}B_{20}PdP_2$): C, 45.80 (45.65); H, 5.89 (5.62).

Pt(bc)(dppe) [3c].

1,1′-Bis-(*o*-carborane) (0.100 g, 0.349 mmol) was added to a 10 mL Schlenk flask, which was then evacuated and backfilled with N_2 three times. Dry THF (4 mL) was transferred to the flask *via* cannula to give a clear solution. While stirring at room temperature, ^{n}BuLi (0.45 mL, 1.6 M, 0.72 mmol) was added drop-wise *via* syringe, giving a dark yellow solution. The reaction was then heated at 60°C for 3 hours, and the color slowly turned lighter yellow. After 3 hours, the flask was allowed to cool to room temperature yielding a golden-yellow solution. Pt(dppe)Cl_2 (0.234 g, 0.349 mmol) was added to a separate 25mL Schlenk flask, which was then evacuated and backfilled with N_2 three times. Dry THF (15 mL) was then transferred *via* cannula to give a white slurry. This flask was cooled to -78°C and stirred for 10 minutes. The dilithio-bis-carborane solution was added dropwise (~1 mL/min) to the cold Pt(dppe)Cl_2 slurry, and the reaction was allowed to warm up to room temperature and stir overnight, giving a deep orange solution the following day. The orange solution was passed through a pad of Celite and washed with THF. The filtrate was then dried *via* rotary evaporation to yield a brown-orange solid. This solid was dissolved in 5-10 mL CH_2Cl_2 and passed through a pad of Celite. After removal of solvent, crude orange product (0.225 g) remained and was purified *via* column chromatography through a pad of basic alumina. A wide 60 mL filter frit was loaded halfway with basic alumina in CH_2Cl_2, and the crude product was dissolved in minimal CH_2Cl_2 (R_f = 0.90) and loaded on the basic alumina. The orange filtrate (mix of product and unreacted bis-carborane) was collected, and the volume was reduced to about 1-2 mL *via* rotary evaporation. Next, 5-10 mL hexanes was added, causing a yellow solid to crash out. This solid was filtered and washed with hexanes (3 x 5 mL) and dried under vacuum for 24 hours to afford **3c** as a yellow powder (0.082 g, 27%). **^{1}H NMR** (CD_2Cl_2, 500 MHz) δ 7.70 (m, 8H, Ar), 7.63 (t, 4H, Ar), 7.55 (m, 8H, Ar), 3.5-0.7 (br. m, 20H, BH), 2.07 (d, 4H, CH_2, $^{2}J_{PH}$ = 17.7 Hz); **^{11}B NMR** (CD_2Cl_2, 160 MHz): -3.67 (m, 4B), -8.08 (m, 16B); **^{13}C NMR** (CD_2Cl_2, 125 MHz) 134.75 (t, $^{2}J_{PC}$ = 5.4 Hz), 132.69, 129.48 (t, $^{3}J_{PC}$ = 5.5 Hz), 126.77 (m), 84.57, 89.12 (dd, bis-carborane C-Pt, $^{2}J_{PC(trans)}$ = 128.4 Hz, $^{2}J_{PC(cis)}$ = 8.34 Hz), 29.86 (m); **^{31}P{^{1}H} NMR** (CD_2Cl_2, 202 MHz): 41.44 (s, $^{1}J_{PtP}$ = 2493 Hz). Single crystals for X-ray diffraction analysis were grown from slow vapor diffusion of diethyl ether into dichloromethane. Elemental analysis found (calculated for $C_{30}H_{44}B_{20}PtP_2$): C, 40.55 (41.04); H, 5.05 (4.80).

Pt(bc)(dtb-bpy) [4a] and [4b].

2 (0.080 g, 0.279 mmol) was charged to a 10mL Schlenk flask, which was then evacuated and backfilled 3 times with N_2. Dry THF (3 mL) was added *via* cannula and the solution was stirred, followed by the addition of ^{n}BuLi (0.36 mL, 1.6 M, 0.576 mmol) at room temperature. The flask was heated at 60°C for 3 hours and then cooled to room temperature. Pt(dtb-bpy)Cl_2 (dtb-bpy = 4,4'-di-*tert*-butyl-2,2'-bipyridine, 0.149 g, 0.279 mmol) was charged to a separate 50mL Schlenk flask, which was evacuated and backfilled 3 times with N_2. Next, dry THF (20 mL) was added, and the resulting yellow slurry was cooled to -78°C. The mixture of Li_2[**bc**] was added dropwise to the cooled slurry. The reaction mixture was allowed to warm up to room temperature and was heated to 60°C. The reaction mixture slowly turned from bright yellow to dark brown/black. After 21 hours, the solvent was moved *via* rotary evaporation to yield a yellow-brown solid. This solid was washed with CH_2Cl_2 (3 x 10 mL), yielding a gray solid (0.082 g) that was emissive under UV excitation (λ_{exc} = 365 nm). To this solid was added 5 mL hot 1,2-difluorobenzene, and this dark brown slurry was heated with a heat gun the solid completely dissolved. Next, the solution was passed through a pad of Celite, yielding a yellow-orange solution with black precipitate at the top of the Celite. This procedure was repeated two more times. The filtrate was dried *in vacuo*, and the pale orange solid was dried under vacuum overnight (0.055 g, 26%). ^{1}H NMR suggests a 1.0:1.4 mix of κ^2-B,C-bound (**4a**) and κ^2-C,C-bound (**4b**) isomers. Single crystals of **4a** for X-ray diffraction analysis were by cooling a concentrated solution of hot 2-MeTHF to room temperature. Attempts to reproduce this result led to products with various mixes of isomers, including both a majority κ^2-B,C-bound isomer and a majority κ^2-C,C-bound isomer, as evidenced by the ^{1}H NMR spectrum of the aryl region (See NMR spectra). HRMS (DART): m/z calculated for $C_{22}H_{44}B_{20}N_2Pt$ $[M+H]^+$, 748.5237 Da; found, 748.5246 Da.

4a: **^{1}H NMR** (THF-d^8, 500 MHz) δ 9.44 (d, 2H, Ar), 8.39 (d, 2H, Ar), 7.92 (dd, 2H, Ar), 3.1-1.0 (br. m, 20H, BH), 1.44 (s, 18H, ^{t}Bu); **^{13}C NMR** (THF-d^8, 125 MHz) 166.32, 157.51, 151.77, 124.54, 121.02, 82.92, 81.01, 36.06, 30.13.

4b: **^{1}H NMR** (THF-d^8, 500 MHz) δ 9.83 (d, 1H, Ar), 9.27 (d, 1H, Ar), 8.41 (d, 1H, Ar), 8.37 (d, 1H, Ar), 7.97 (dd, 1H, Ar), 7.84 (dd, 1H, Ar), 4.47 (s, 1H, $CH_{bis\text{-}carborane}$), 3.2-1.6 (br m, 20H, BH), 1.459 (9H, s, ^{t}Bu), 1.457 (9H, s, ^{t}Bu); **^{13}C NMR** (THF-d^8, 125 MHz) 165.44, 165.00, 157.99, 156.52,

154.24, 150.23, 125.15, 124.52, 121.12, 120.36, 85.17, 77.03, 70.52, 68.19, 36.53, 36.46, 30.27, 30.14.

Note: ^{11}B NMR chemical shifts for 4a and 4b overlap, so values for the mixture are reported. **^{11}B NMR** (THF-d^8, 160 MHz) -4.01 (4B), -8.79 (8B), -11.15 (8B).

Pt(tebc)(dtb-bpy) [8].

7 (0.0689 g, 0.171 mmol, **tebc**) was charged to a 10mL Schlenk flask, which was then evacuated and backfilled 3 times with N_2. Dry THF (2 mL) was added *via* cannula and the solution was stirred, followed by the slow addition of ^{n}BuLi (0.22 mL, 1.6 M, 0.351 mmol) at room temperature. The flask was heated at 60°C for 3 hours and then cooled to room temperature. Pt(dtb-bpy)Cl_2 (dtb-bpy = 4,4'-di-*tert*-butyl-2,2'-bipyridine, 0.0914 g, 0.171 mmol) was charged to a separate 25mL Schlenk flask, which was evacuated and backfilled 3 times with N_2. Next, dry THF (15 mL) was added, affording a yellow slurry, which was cooled to -78°C. The mixture containing [Li_{2}-(**tebc**)] was added dropwise to the cooled slurry. The reaction mixture was allowed to warm up to room temperature and was then heated at 60°C for 60 hours. The reaction slowly turned from bright yellow to yellow-brown. After 60 hours, the solvent was moved *via* rotary evaporation to yield a yellow-brown solid. This solid was dissolved in diethyl ether and passed through a pad of Celite, and the filtrate was then dried *in vacuo*. Next, the resulting solid was washed with cold pentane (5 x 2 mL), yielding a pale orange solid. The solid was dissolved in CH_2Cl_2 and passed through a silica plug. The filtrate was dried *in vacuo*, and the resulting solid was dried under vacuum overnight (0.063 g, 43%). **^{1}H NMR** (CD_2Cl_2, 500 MHz) δ 9.56 (d, 1H, Ar), 9.36 (d, 1H, Ar), 8.08 (d, 1H, Ar), 8.02 (d, 1H, Ar), 7.68 (dd, 1H, Ar), 7.47 (dd, 1H, Ar), 3.87 (s, 1H, $CH_{bis\text{-}carborane}$), 3.3-1.2 (16H, br. m, BH), 1.45 (9H, s, ^{t}Bu), 1.43 (9H, s, ^{t}Bu), 1.02-0.82 (m, 12H, CH_2CH_3), 0.56-0.79 (m, 8H, CH_2); **^{11}B NMR** (CD_2Cl_2, 160 MHz) 7.74 (m, 4B), -8.46 (s, 8B), -11.25 (s, 4B), -13.81 (s, 4B); **^{13}C NMR** (CD_2Cl_2, 125 MHz) 164.12 (s, C^2 dtb-bpy, C—Pt_{trans}), 163.58, 157.22, 155.85, 155.19, 149.79, 123.99, 123.77, 120.02, 119.17, 76.63, 73.20, 59.56, 55.39, 35.98, 35.90, 30.36, 30.23 (d, *tert*-butyl CH_3, J = 16.2 Hz), 13.95, 13.71, 13.57, 13.51, (ethyl CH_3), 9.11 (br. m, ethyl CH_2). Single crystals for X-ray diffraction analysis were grown the slow evaporation of diethyl ether over the course of one week. HRMS (Orbitrap): m/z calculated for $C_{30}H_{64}B_{20}N_2Pt$ [M^-], 863.6724 Da; found, 863.0652 Da.

[NEt_4]$_2$[Pd(bc)$_2$]

2 (0.100 g, 0.349 mmol) was added to a 25 mL Schlenk flask, which was then evacuated and backfilled with N_2 three times. Dry THF (4 mL) was transferred to the flask *via* cannula, generating a clear solution. While stirring at room temperature, ^{n}BuLi (0.45 mL, 1.6 M, 0.72 mmol) was added dropwise *via* syringe to give a dark yellow solution. The reaction was then heated to 60°C for 3 hours, and the color slowly turned lighter yellow. After 3 hours, the flask was cooled to room temperature, and under positive N_2 flow, $PdCl_2$ (0.0309 g, 0.175 mmol) was added. The temperature was increased to 60°C and the mixture was allowed to stir for 21 hours. The reaction was cooled to room temperature, passed through a pad of Celite, and the filtrate was dried *in vacuo*. The resulting brown solid was then dissolved in H_2O (10 mL). To this solution was added a solution of tetraethylammonium bromide (NEt_4Br, 0.0736 g, 0.349 mmol) dissolved in 2 mL H_2O, and brown solid immediately precipitated. The solid was filtered and washed with H_2O (3 x 10 mL) was then dissolved in CH_2Cl_2 and dried over Na_2SO_4. The mixture was filtered and the solvent removed *via* rotary evaporation. Next, the solid was dissolved in minimal CH_2Cl_2 and loaded on silica. The plug was flushed with CH_2Cl_2 (100 mL) to remove side products, and then flushed with acetone (50 mL) to collect the product. Solvent was removed *via* rotary evaporation, and the brown solid was vacuum dried (0.059 g, 25%). *Note: We believe B—H activation and/or deboronation may be occurring under the given reaction conditions for* ***[NEt_4]$_2$[Pd(bc)$_2$]****; however, the ^{11}B NMR spectrum for* ***[NEt_4]$_2$[Pd(bc)$_2$]*** *isolated under these conditions are in agreement with the ^{11}B NMR spectrum for the side product isolated during the synthesis of* ***3b*** *(see Figure S7 below).* **^{1}H NMR** (CD_2Cl_2, 500 MHz) δ 3.4-1.0 (br. m, 20H, BH), 3.21 (q, 16H, CH_2), 1.35 (tt, 24H, CH_3); **^{11}B NMR** (CD_2Cl_2, 160 MHz) -2.66 (m, 9B), -9.31 (m, 26B),-16.75 (2B), -22.07 (2B), -32.64 (0.5B), -35.11 (0.5B); **^{13}C NMR** (CD_2Cl_2, 125 MHz) 125.77, 82.77, 82.39, 72.30, 65.49, 65.22, 63.25, 34.46, 30.44, 30.05, 7.87. Elemental analysis found (calculated for $C_{24}H_{80}B_{40}N_2Pd$): C, 32.68 (30.81); H, 8.58 (8.62).

[NEt_4]$_2$[Pt(bc)$_2$]

2 (0.100 g, 0.349 mmol) was added to a 25 mL Schlenk flask, which was then evacuated and backfilled with N_2 three times. Dry THF (4 mL) was transferred to the flask *via* cannula, generating a clear solution. While stirring at room temperature, ^{n}BuLi (0.45 mL, 1.6 M, 0.72

mmol) was added dropwise *via* syringe to give a dark yellow solution. The reaction mixture was then heated at 60°C for 3 hours, and the color slowly turned lighter yellow. After 3 hours, the flask was cooled to -78°C, and under positive N_2 flow, $Pt(cod)Cl_2$ (cod = 1,5-cyclooctadiene, 0.065 g, 0.175 mmol) was added. The temperature was increased to 60°C and the resulting mixture was allowed to stir for 21 hours. The resulting dark brown mixture was allowed to cool to room temperature and was passed through a pad of Celite. The filtrate was dried *in vacuo*. The resulting solid was then dissolved in H_2O (10 mL). To this solution was added a solution of tetraethylammonium bromide (NEt_4Br, 0.0736 g, 0.349 mmol) dissolved in 2 mL H_2O, and dark green solid immediately precipitated. The solid was filtered and washed with H_2O (3 x 10 mL) and was then dissolved in CH_2Cl_2 and dried over Na_2SO_4. The dark brown solution was filtered and the solvent removed *via* rotary evaporation. Next, the solid was dissolved in minimal CH_2Cl_2 and loaded on silica. The plug was flushed with CH_2Cl_2 (100 mL) to remove side products, and then flushed with acetone (50 mL) to collect the product. Solvent was removed *via* rotary evaporation, and the resulting brown solid was vacuum dried (0.182 g, 68%). *Note: We believe B—H activation and/or deboronation may be occurring under the given reaction conditions for* ***$[NEt_4]_2[Pt(bc)_2]$****; however, the ^{11}B NMR spectrum for* ***$[NEt_4]_2[Pt(bc)_2]$*** *isolated under these conditions are in agreement with the ^{11}B NMR spectrum for the side product isolated during the synthesis of* ***3c*** *(see Figure S7 below).* **^{1}H NMR** (CD_2Cl_2, 500 MHz): 3.3-1.0 (br. m, 20H, BH), 3.21 (q, 16H, CH_2), 1.35 (tt, 24H, CH_3); **^{11}B NMR** (CD_2Cl_2, 160 MHz) -2.84 (m, 8B), -9.45 (m, 25H), -16.94 (d, 4B), -22.25 (d, 2B), -32.77 (d, 0.5B), -35.21 (d, 0.5B); **^{13}C NMR** (CD_2Cl_2, 125 MHz) 72.31, 71.22, 65.43, 63.26, 29.88, 22.72, 14.21, 7.86. Elemental analysis found (calculated for $C_{24}H_{80}B_{40}N_2Pt$): C, 31.37 (28.14); H, 7.56 (7.87).

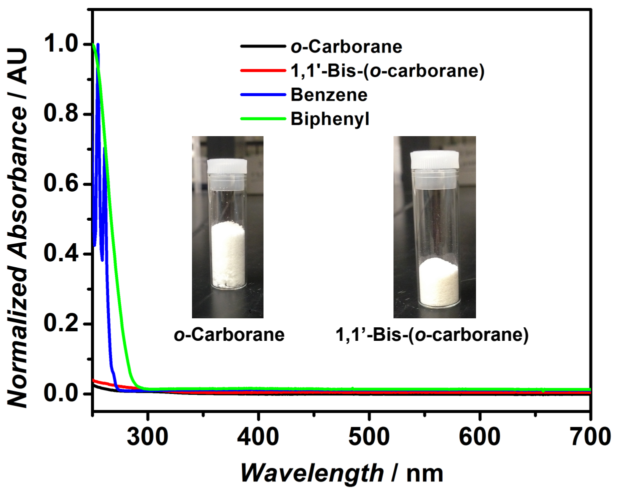

Figure S1: UV-Vis absorption spectrum of **1** and **2**. Solutions were made to 0.002 M concentration.

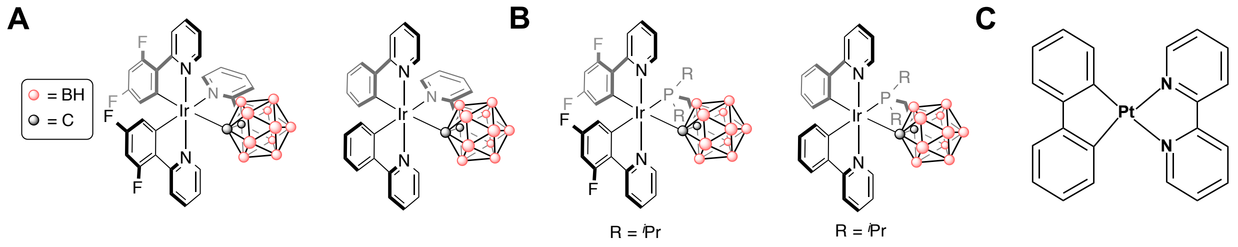

Figure S2: Structures of luminescent compounds mentioned in manuscript. A) Ir(III) compounds containing a κ^2-C,N-bound 1-(2-pyridyl)-*o*-carboranyl ligand (page 1 of manuscript, reference 13, SI); B) Ir(III) compounds containing a κ^2-C,P-bound 1-($^i Pr_2PCH_2$)-*o*-carboranyl (page 1 of manuscript, reference 14, SI); C) Pt(bph)(bpy) where bph = biphenyl and bpy = 2,2′-bipyridine (page 2 of manuscript, reference 15, SI).

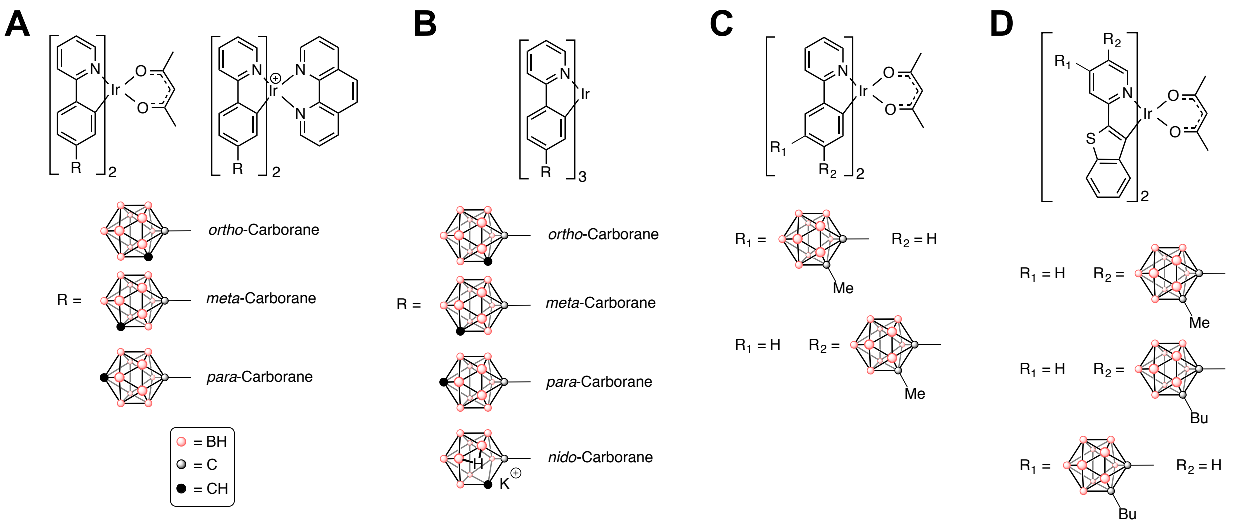

Figure S3: Structures of Ir(III) compounds that contain *ortho*-, *meta*-, *para*, and *nido*-carborane as substituents. A) Reference 16; B) Reference 17; C) Reference 18; D) Reference 19.

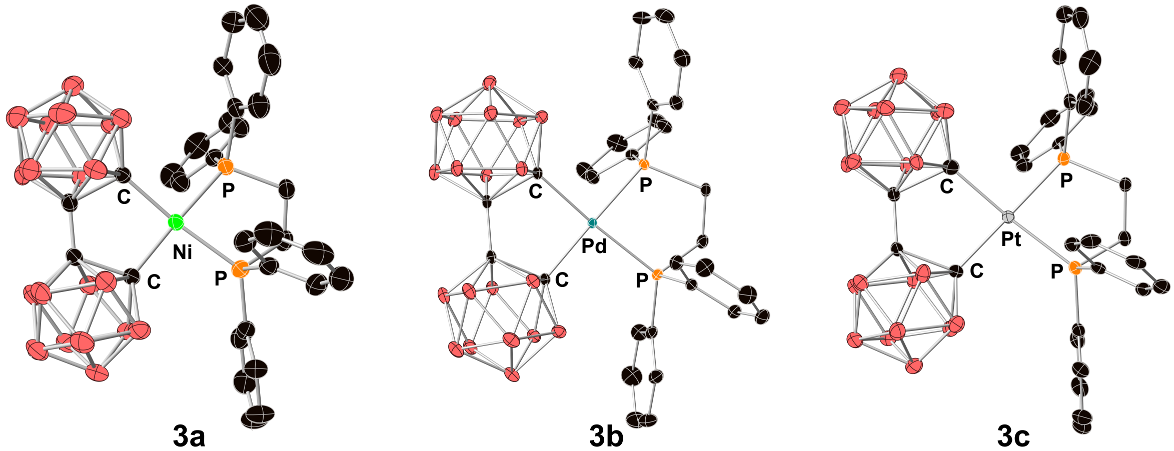

3a
Ni(bc)(dppe)

3b
Pd(bc)(dppe)

3c
Pt(bc)(dppe)

Figure S4: Thermal ellipsoid plots of **3a**-**3c** at 50% probability.

Table S1: Selected bond distances and angles for **3a**-**3c**.

Parameter	3a	3b	3c
**M-C1 (Å)**	2.007	2.097	2.090
**M-C4 (Å)**	1.995	2.103	2.093
**C1-C2 (Å)**	1.694	1.688	1.744
**C2-C3 (Å)**	1.514	1.531	1.514
**C3-C4 (Å)**	1.694	1.690	1.728
**M-P1 (Å)**	2.253	2.312	2.297
**M-P2 (Å)**	2.243	2.304	2.310
**C1-M-C4 (°)**	90.82	88.20	86.88
**M-C1-C2 (°)**	111.25	111.60	113.48
**C1-C2-C3 (°)**	113.04	114.43	112.52
**C2-C3-C4 (°)**	113.40	114.18	113.69
**C3-C4-M (°)**	111.43	111.51	113.36
**C1-M-P2 (°)**	171.46	174.30	173.20
**C4-M-P1 (°)**	172.40	174.71	173.05
**M-M (Å)**	10.976	12.176	8.685
**C1-C4 (Å)**	2.850	2.923	2.876
**C1-C2-C3-C4 dihedral**	-0.2	-2.91	0.59
**C1-C2-C3-C4-M sum of angles (°)**	539.94	539.92	539.93

Table S2: Selected bond distances and angles for **3a-3c** and the average bond distances and angles of corresponding M(bph)(P^P) complexes.

Parameter	3a	Avg. Ni(bph)(P^P)	3b	Avg. Pd(bph)(P^P)	3c	Avg. Pt(bph)(P^P)
**M-C1 (Å)**	2.007	1.960	2.097	2.050	2.090	2.050
**M-C4 (Å)**	1.995	1.940	2.103	2.050	2.093	2.030
**C1-M-C4 (°)**	90.82	83.25	88.20	80.82	86.88	80.66

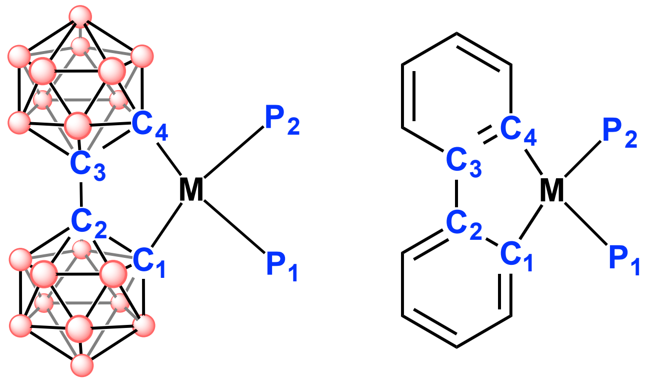

Figure S5: Numbering scheme for values presented in Table S1 and Table S2. Left: M = Ni, **3a**; M = Pd, **3b**; M = Pt, **3c**. Right: M(bph)(P^P), where M = Ni, Pd, Pt and (P^P) = chelated diphosphine ligand.

To demonstrate that the bc framework is structurally similar to the biphenyl (bph) framework, we compared bond distances, angles, and molecular geometries of M(bc)(dppe) to those of a series of cyclometallated M(bph)(P^P) in reported X-ray crystal structures.[20] X-ray quality crystals of **3a**-**3c** were grown using slow evaporation of diethyl ether, vapor diffusion of diethyl ether into dichloromethane, or cooling of a concentrated solution of hot benzene. For **3a**-**3c**, the M—C bond is about 0.05 Å longer than the average M—C bond in corresponding biphenyl complexes. Of the three complexes **3a**-**3c**, the Ni—C bond (**3a**) is the shortest M—C bond at 2.00 Å, and both Pd—C (**3b**) and Pt—C (**3c**) bond lengths are about 2.09 Å. These observations are consistent with Ni possessing the smallest atomic radius and Pd and Pt exhibit similar atomic radii due to the lanthanide contraction for Pt.[21]

The C1—M—C4 bond angles decrease as the size of the metal atom increases, which compares well with analogous Group 10 bph complexes. However, the trend is more pronounced in the **bc** series with this angle being 6-8° larger than the average C1—M—C4 angle in the biphenyl complexes. The C1—C2—C3—C4 dihedral angles of 0.20°, 2.91°, and 0.59° for **3a**, **3b**, and **3c**, respectively, suggest the chelation of **2** leads to a minimally distorted square planar structure. The C1—C4 distances between the two metal-bound carbons fall within a range of 0.07 Å of each other (2.850 Å to 2.923 Å), indicating the larger C1—M—C4 angle is likely a structural characteristic of **2**. For comparison, the average corresponding C1—C4 distance in M(bph)(P^P) complexes is about 0.2 Å shorter at 2.662 Å, further rationalizing the more acute C1—M—C4 angles seen in those bph complexes. Furthermore, the **bc** C1—M—C4 bond angles

are larger than all reported C1—M—C4 bond angles for M(bph)(P^P) complexes. Compounds **3a**-**3c** have C—M—P_{trans} angles of 171-174°, making them slightly distorted square planar complexes. These values are comparable to those of the reported cyclometallated M(bph)(P^P) compounds, indicating that **bc** does not influence the intramolecular geometry much differently than biphenyl.

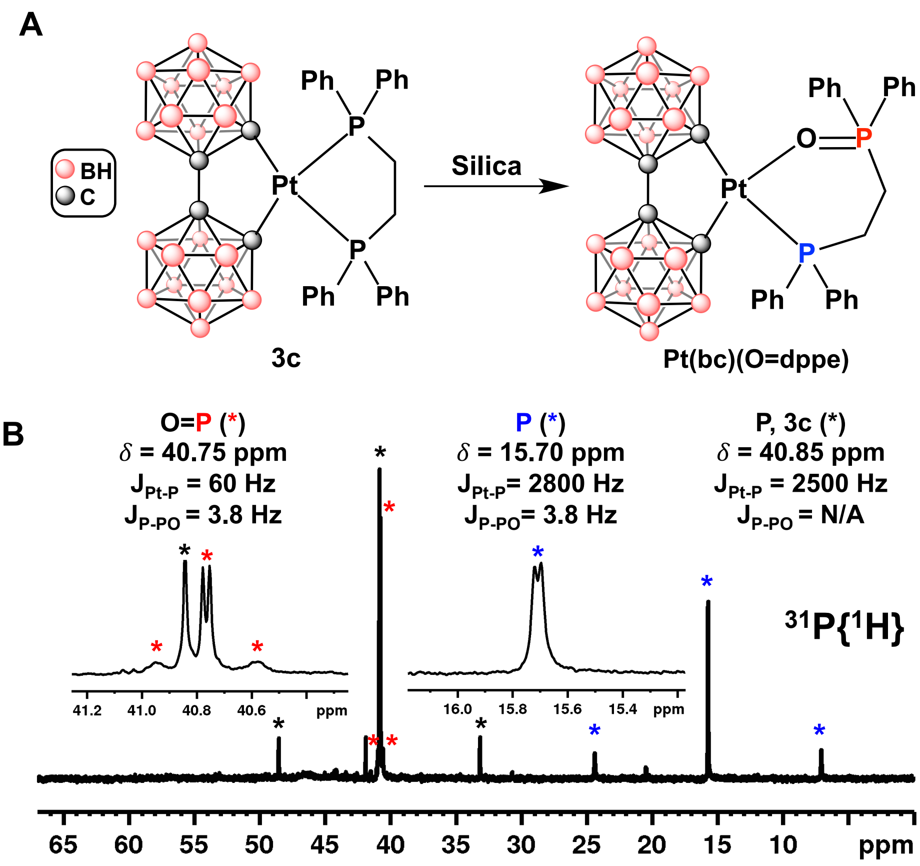

Figure S6: A) Reaction scheme for the oxidation of **3c** on silica to form **Pt(bc)(O=dppe)**. B) $^{31}P\{^{1}H\}$ NMR spectrum of the product **Pt(bc)(O=dppe)** isolated after passing **3c** through a short plug of silica eluted with CH_2Cl_2. Insets show expanded regions of the ^{31}P NMR spectrum corresponding to each of the phosphorous atoms in the dppe ligand. Asterisks correspond to the ^{31}P peak and Pt—P satellites for each phosphorous: black = both P atoms in **3c**; red = O=P in **Pt(bc)(O=dppe)**; blue = P in **Pt(bc)(O=dppe)**.

The purification of **3c** proved troublesome as one of the P—Pt bonds easily underwent oxidation on silica, forming the monooxidized dppe species as evidenced by the formation of 2 doublets with unique $^{1}J_{Pt\text{-}P}$ values in the $^{31}P\{^{1}H\}$ NMR spectrum. For the oxidized P (O=P, red in Figure 3A), δ = 40.75 ppm with $^{2}J_{Pt—P}$ = 60 Hz and $^{3}J_{P—PO}$ = 3.8 Hz. For the unoxidized P (P, blue in Figure 3A), δ = 15.70 ppm with $^{1}J_{Pt—P}$ = 2800 Hz and $^{3}J_{P—PO}$ = 3.8 Hz. These chemical shifts and coupling constants are similar to those for compounds **2a** and **2b** in Reference 22. Purifying the crude reaction mixture for **3c** *via* column chromatography with basic alumina yielded the unoxidized, isolated compound at 27% yield.

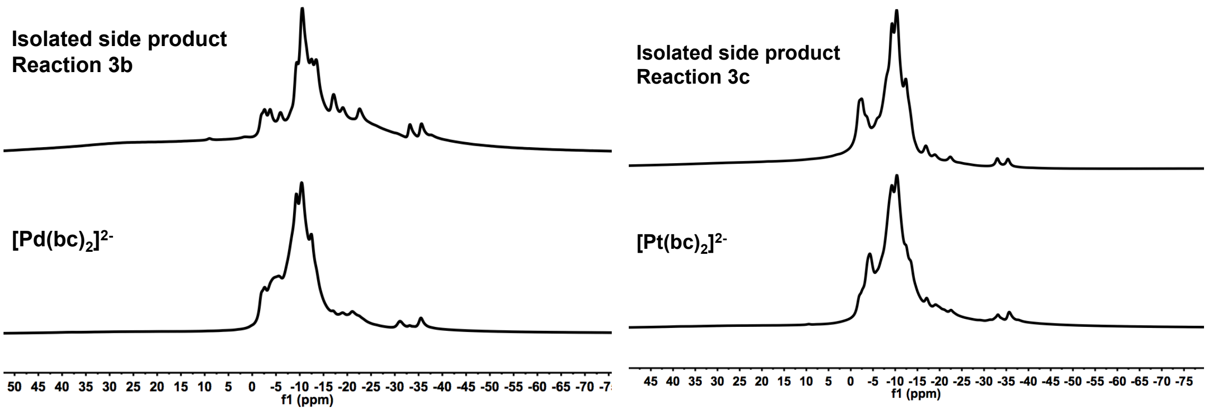

Figure S7: Left: Stacked $^{11}B\{^1H\}$ NMR spectra for the $\mathbf{[Pd(bc)_2]^{2-}}$ side product isolated during the synthesis of **3b** (top) and independently synthesized $\mathbf{[Pd(bc)_2]^{2-}}$ (see experimental). Right: Stacked $^{11}B\{^1H\}$ NMR spectra for the $\mathbf{[Pt(bc)_2]^{2-}}$ side product isolated during the synthesis of **3c** (top) and independently synthesized $\mathbf{[Pt(bc)_2]^{2-}}$ (see experimental).

For each reaction, the other major by-product is $\mathbf{[M(bc)_2]^{2-}}$ (where M = Ni, Pd, Pt), which is isolated *via* silica column chromatography. The formation of this di-substituted complex is likely due to the low concentration of M(dppe)Cl_2 relative to newly generated and more soluble M(bc)(dppe) under the reaction conditions, making M(bc)(dppe) more prone to a second nucleophilic attack from the $\mathbf{Li_2}$**-[bc]**. The ^{11}B NMR of the isolated reaction product for **3b** and **3c** matched the ^{11}B NMR of the independently synthesized $\mathbf{[Pd(bc)_2]^{2-}}$ and $\mathbf{[Pt(bc)_2]^{2-}}$ complexes (Figure S5), supporting our hypothesis.

We believe B—H activation and/or deboronation may be occurring under the given reaction conditions for $\mathbf{[Pd(bc)_2]^{2-}}$ and $\mathbf{[Pt(bc)_2]^{2-}}$, and that the reaction product contains admixtures of ***closo-nido*-[bc]** and/or its metalation products. Kazakov and coworkers have reported the partial deboronation of **2** in the presence of water,[23] and their reported ^{11}B NMR chemical shifts are similar to those observed in for $\mathbf{[Pd(bc)_2]^{2-}}$ and $\mathbf{[Pt(bc)_2]^{2-}}$ (Figure S7). However, the ^{11}B NMR spectra for $\mathbf{[Pd(bc)_2]^{2-}}$ and $\mathbf{[Pt(bc)_2]^{2-}}$ isolated under these conditions are in agreement with the ^{11}B NMR spectra for the side product isolated during the synthesis of **3b** and **3c**.

Additionally, ^{31}P NMR shows the *in-situ* formation of mono-oxidized dppe, $Ph_2P(CH_2)_2P(O)Ph_2$ (**O=dppe**) for the synthesis of **3a-3c**, suggesting the ligand is displaced by an extra equivalent of $\mathbf{Li_2}$**-[bc]** as the metal center is substituted twice (Figure S6). The $^{31}P\{^1H\}$ chemical shifts for **O=dppe** are similar to those for compound **2** in Reference 24. Interestingly, we still observe free **O=dppe** despite stringent air-free conditions.

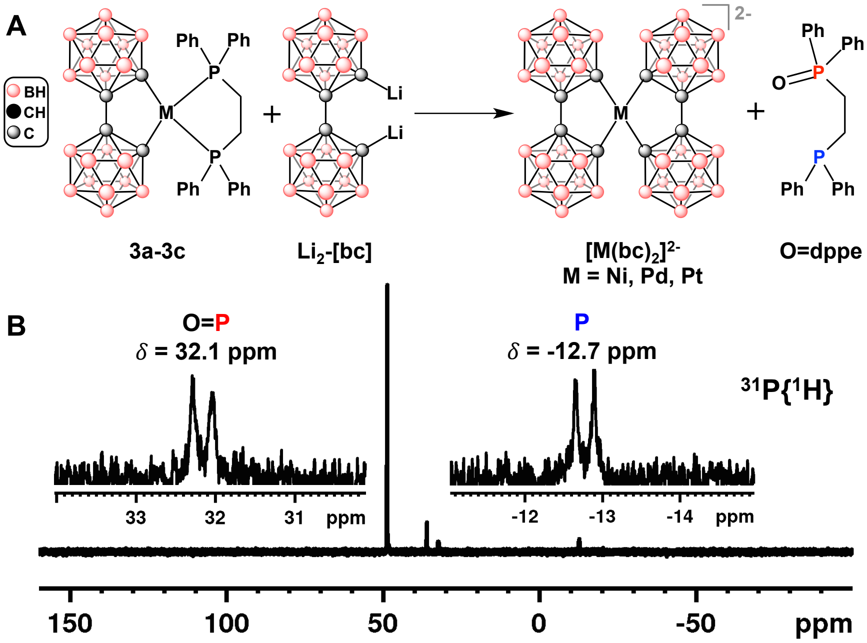

Figure S8: A) Reaction scheme for the formation of **$[M(bc)_2]^{2-}$** (M = Ni, Pd, Pt) and **O=dppe**. B) Representative *in-situ* $^{31}P\{^1H\}$ NMR spectrum during the synthesis of **3a** shows the formation of free **O=dppe** (δ = 32.1 ppm, O=P; δ = -12.7 ppm, P), which results from the reaction of **Li_2-[bc]** with **3a** (δ = 49.09 ppm) to form **$[Ni(bc)_2]^{2-}$**. Interestingly, **O=dppe** formed even under rigorous oxygen-free conditions.

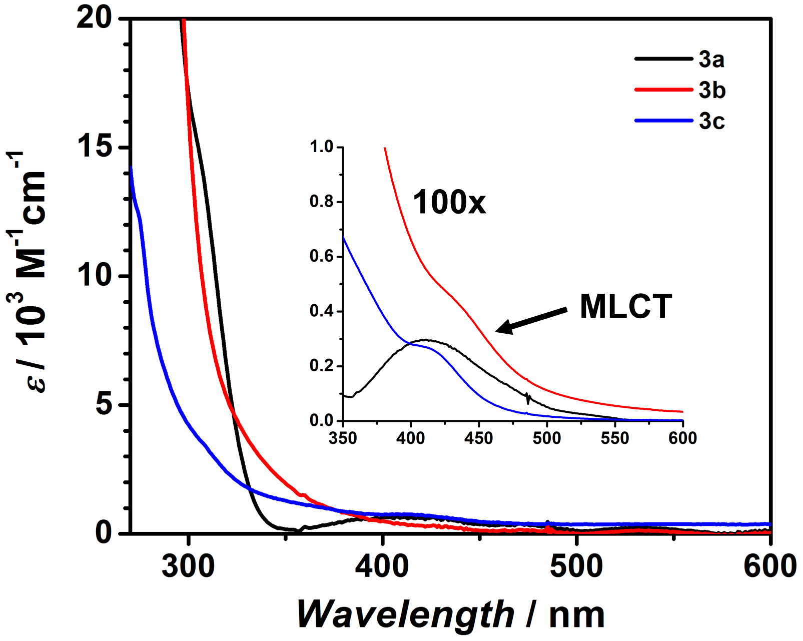

Figure S9: UV-Vis absorption spectrum for **3a** (black), **3b** (red), and **3c** (blue). Inset: Zoomed in region depicting MLCT transitions.

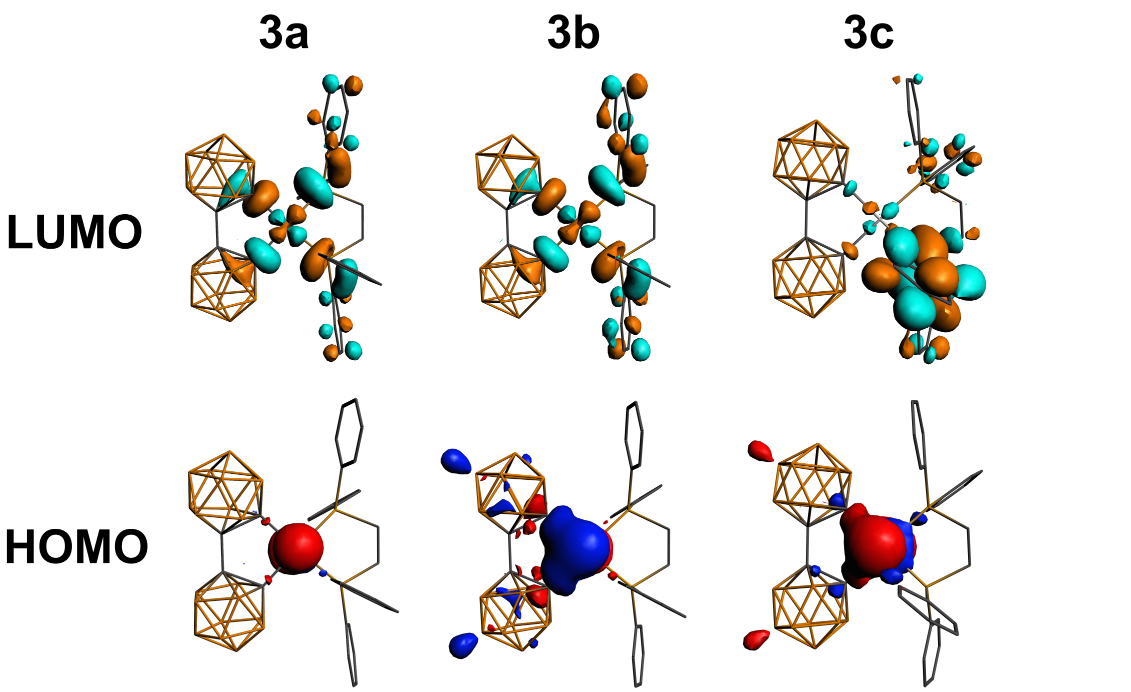

Figure S10: HOMO and LUMO images derived from DFT calculations of **3a** (left), **3b** (middle), and **3c** (right). Involvement of the **bc** ligand in HOMO-LUMO transitions is negligible, indicating the **bc** ligand is photophysically innocent.

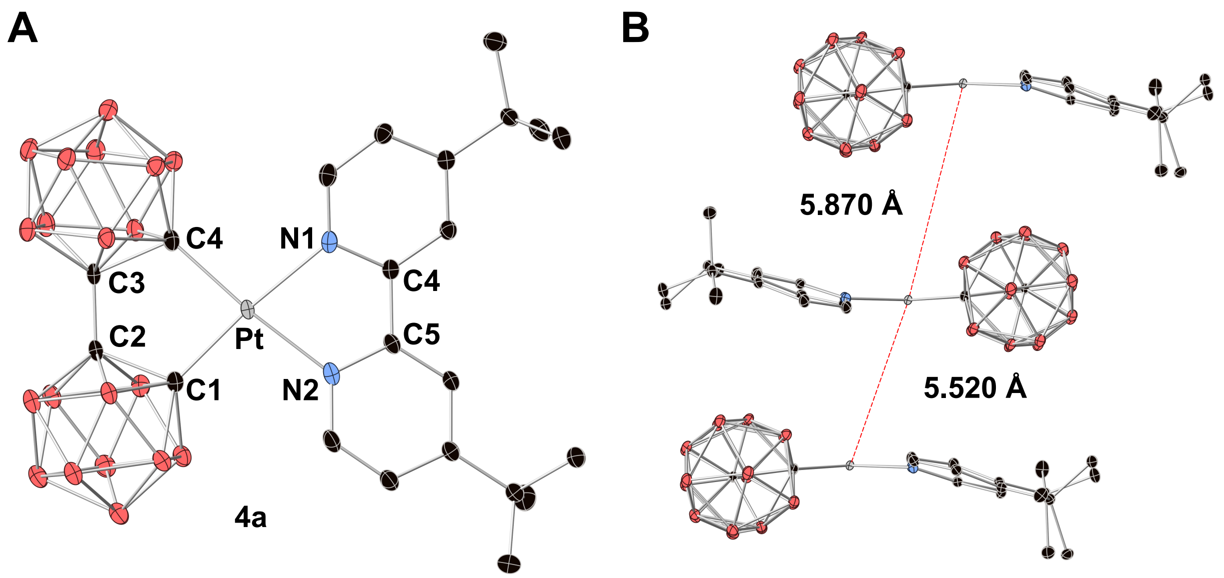

Figure S11: A) Thermal ellipsoid plot of **4a** (50% probability). H atoms were omitted for clarity. B) Stacking of **4a** Pt(II)···Pt(II) distances of 5.870 Å and 5.520 Å.

Table S3: Selected bond distances and angles for **4a**.

Parameter	4a
**Pt-C1 (Å)**	2.043
**Pt-C4 (Å)**	2.045
**C1-C2 (Å)**	1.708
**C2-C3 (Å)**	1.516
**C3-C4 (Å)**	1.71
**Pt-N1 (Å)**	2.109
**Pt-N2 (Å)**	2.105
**C1-Pt-C4 (°)**	86.54
**Pt-C1-C2 (°)**	114.67
**C1-C2-C3 (°)**	112.04
**C2-C3-C4 (°)**	112.15
**C3-C4-Pt (°)**	114.43
**C1-Pt-N1 (°)**	174.27
**C4-Pt-N2 (°)**	173.08
**Pt···Pt (Å)**	5.87
**C1-C4 (Å)**	2.802
**C1-C2-C3-C4 dihedral (°)**	1.22
**N1-C4-C5-N2 dihedral (°)**	1.27

Table S4: Selected bond distances and angles for **4a** and the selected average bond distances and angles of Pt(bph)(N^N) complexes.

Parameter	4a	Pt(bph)(N^N)
**Pt-C1 (Å)**	2.043	2.011
**Pt-C4 (Å)**	2.045	1.996
**Pt-N1 (Å)**	2.091	2.106
**Pt-N2 (Å)**	2.105	2.126
**C1-Pt-C4 (°)**	86.54	80.76
**C1-Pt-N1 (°)**	174.27	151.44
**C4-Pt-N2 (°)**	173.08	151.33

For **4a**, both Pt—C bond lengths are almost identical at 2.043 Å and 2.045 Å, and they are only 0.04 Å longer than the average Pt—C bond found in all Pt(bph)(N^N) crystal structures available in the Cambridge Structural Database. At 2.105 Å and 2.091 Å, Pt—N bonds in this

molecule are roughly the same as the average Pt—N bond in Pt(bph)(N^N) compounds, with the difference in length of about 0.02-0.03 Å. The C—Pt—C angle in the **bc** analogue is about 6° larger than the average corresponding angle in all N^N chelated biphenyl analogues (86.54° versus 80.76°), which is characteristic of **bc**-chelated metal ions reported. This C—M—C angle for **4a** is also consistent with the C—M—C angle seen in **3a-3c** (Table S2). Based on this evidence, the intramolecular geometry of the synthesized **bc** complex is roughly equivalent to that of its biphenyl analogues.

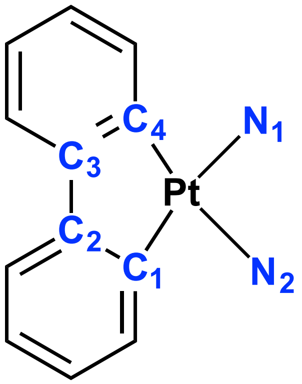

Figure S12: Numbering scheme for **Pt(bph)(N^N)** complexes listed in Table S3 and Table S4.

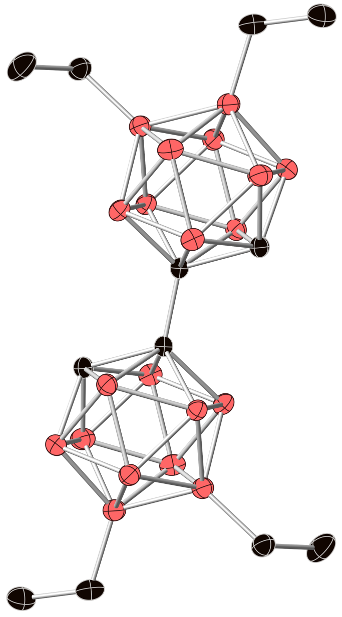

Figure S13: Thermal ellipsoid plot of **7** (50% probability). Salmon = B; black = C. H atoms were omitted for clarity.

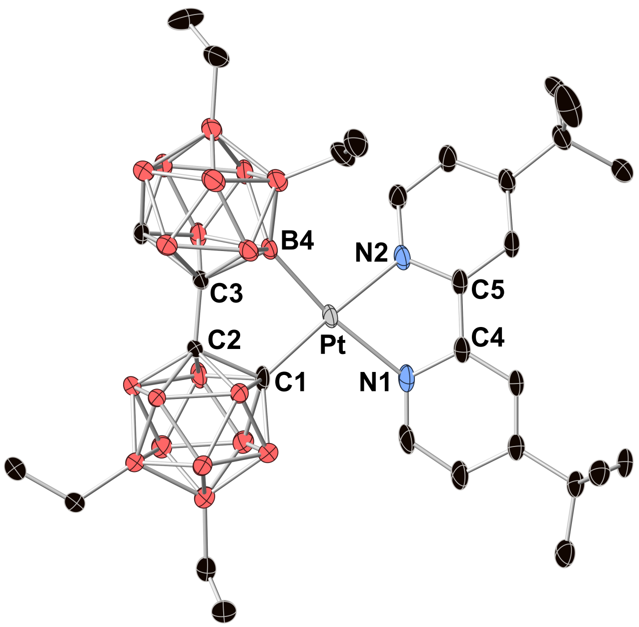

Figure S14: Thermal ellipsoid plot of **8** (50% probability). H atoms were omitted for clarity. Labels indicate the numbering scheme used for Table S5.

Table S5: Selected bond angles and distances for **8**.

**Parameter**	**8**
**Pt-C1 (Å)**	2.036
**Pt-B4 (Å)**	2.073
**C1-C2 (Å)**	1.704
**C2-C3 (Å)**	1.517
**C3-B4 (Å)**	1.725
**Pt-N1 (Å)**	2.175
**Pt-N2 (Å)**	2.053
**C1-Pt-B4 (°)**	87.02
**Pt-C1-C2 (°)**	115.78
**C1-C2-C3 (°)**	110.73
**C2-C3-B4 (°)**	114.11
**C3-B4-Pt (°)**	112.04
**C1-Pt-N2 (°)**	176.55
**B4-Pt-N1 (°)**	168.82
**Pt···Pt (Å)**	7.979
**C1-B4 (Å)**	2.829
**C1-C2-C3-B4 dihedral (°)**	6.1
**N1-C4-C5-N2 dihedral (°)**	4.43

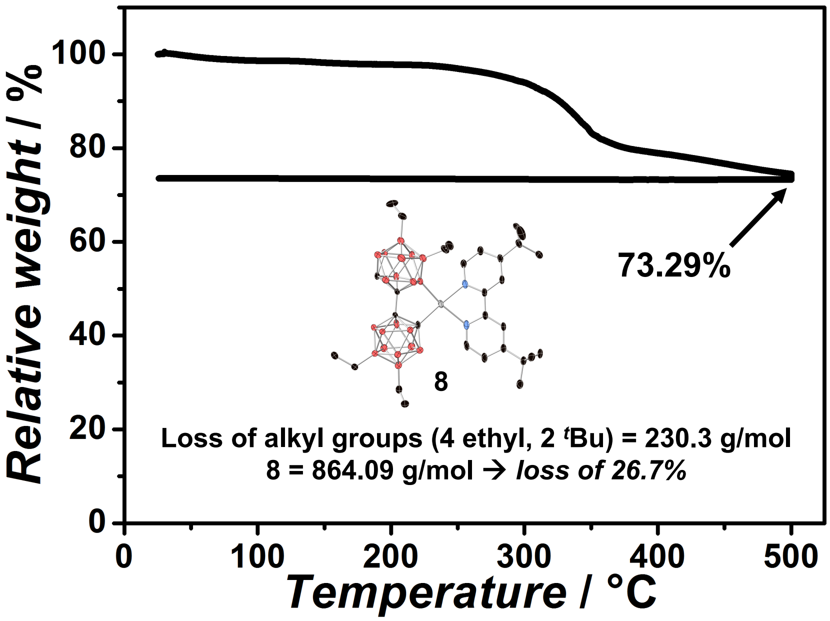

Figure S15: Thermogravimetric analysis (TGA) of compound **8** up to 500°C. The **bc** framework remains intact, and the loss in mass corresponds to the cleavage of alkyl groups from both ligands.

Geometry Optimized Coordinates for DFT Calculations.

Ni(bc)(dppe) [3a].

Atom	X	Y	Z
Ni	0.68130784	7.86224652	3.24437611
P	-1.26868014	6.72001114	3.49170712
P	-0.35978197	8.8625613	1.48195488
B	2.994782	9.96752888	1.84304495
H	2.15585703	10.70107025	1.43005302
B	3.23819456	8.35001487	1.20090549
H	2.57821322	7.90811987	0.32079833
B	4.91677016	7.86346719	1.51662324
H	5.47736619	7.09155985	0.80188353
B	5.72325876	9.20504807	2.36574499
H	6.89104651	9.4186741	2.25652971
B	4.52873294	10.51587323	2.56025468
H	4.80735364	11.67367185	2.6067092
B	3.60506102	7.28269331	2.53582347
H	3.21624832	6.17007578	2.57232808
B	5.14459512	7.79364483	3.28269304

H	5.78629056	6.99009515	3.87502496
B	4.90873847	9.4372323	3.92810226
H	5.38900024	9.76040877	4.96402319
B	3.22580874	9.91603121	3.57803344
H	2.60670085	10.57563694	4.33280972
B	4.54729128	9.54722488	1.06994424
H	4.85107648	10.00636013	0.01242494
B	2.60772569	6.03541539	5.17769788
H	2.89773382	5.35984693	4.25706749
B	3.84885902	6.65963133	6.29942675
H	4.98880656	6.41497752	6.07806891
B	3.34270067	8.28910086	6.8096799
H	4.13515	9.16323281	6.93920908
B	1.79192645	8.64829172	5.99882394
H	1.50448676	9.72803552	5.62248972
B	1.08329627	5.89959664	6.02883733
H	0.3122927	5.08756852	5.63067479
B	0.5869789	7.50365337	6.54357491
H	-0.54352648	7.85927784	6.52479365
B	1.79673804	8.1336022	7.682261
H	1.4943848	8.91833046	8.52716949
B	3.06178246	6.89127174	7.87581888
H	3.68343158	6.764142	8.88497246
B	2.61071988	5.5001382	6.85356997
H	2.90324816	4.37040944	7.09598034
B	1.35053747	6.40831532	7.7173315
H	0.71775639	5.93315735	8.60900253
C	-1.91952823	7.91177759	1.08800196
H	-2.58281384	8.52963135	0.46663606
H	-1.62387702	7.03743491	0.49123538
C	-2.5705466	7.48548301	2.38974153
H	-3.38946478	6.76892979	2.23778317
H	-2.98066464	8.35282125	2.92527119
C	-1.0474131	5.05443258	2.73237353
C	0.24288978	4.63942213	2.36840491
H	1.09707882	5.28630376	2.58010927
C	0.43949325	3.40309725	1.74601711
H	1.4484258	3.09148819	1.47218807
C	-0.65088629	2.57185862	1.48147561
H	-0.49763654	1.60539699	0.99872821
C	-1.94167344	2.97750644	1.84029941
H	-2.795531	2.32848128	1.63963107
C	-2.1411169	4.21143655	2.46129865

H	-3.15141682	4.50912923	2.7478294
C	-2.29594903	6.45549847	5.0041215
C	-2.9761234	7.55956098	5.54956077
H	-2.85121997	8.55445379	5.11901217
C	-3.79819518	7.40497557	6.66549029
H	-4.31677757	8.27138693	7.07805176
C	-3.94312428	6.14780841	7.26001555
H	-4.58120248	6.02752767	8.13671425
C	-3.25883008	5.0500927	6.7342386
H	-3.35722195	4.06842137	7.19976275
C	-2.43748049	5.20047844	5.61354259
H	-1.90696793	4.3356255	5.21768429
C	0.21883843	9.1871958	-0.24334024
C	0.47520604	10.48188264	-0.71773625
H	0.37231565	11.33895554	-0.0535171
C	0.86485767	10.68321591	-2.04459738
H	1.06303568	11.69593879	-2.39807745
C	1.00698938	9.59669255	-2.90997871
H	1.31491646	9.75637099	-3.94422614
C	0.7641221	8.30161687	-2.44222271
H	0.88608483	7.44563465	-3.10727826
C	0.37758482	8.09670953	-1.11759315
H	0.21941031	7.07633048	-0.76518842
C	-1.02072573	10.47452079	2.08785902
C	-0.566376	10.97767514	3.31674014
H	0.19465376	10.42901651	3.87602975
C	-1.07752853	12.17621948	3.82380492
H	-0.71197908	12.55802354	4.77801063
C	-2.04553305	12.88221701	3.10668539
H	-2.44294202	13.81922213	3.50026345
C	-2.5049618	12.38832435	1.88015861
H	-3.25782244	12.93995193	1.31483395
C	-1.99676589	11.19210177	1.37164925
H	-2.35156691	10.82760083	0.40602493
C	2.48255388	8.57661818	2.7522921
C	3.63924617	8.28576866	3.95188095
C	1.39900971	7.28054514	5.02025565
C	3.02725268	7.70851603	5.22368025

Pd(bc)(dppe) [3b].

Atom	X	Y	Z
Pd	15.58256413	8.49645518	6.2289578
P	15.01837909	8.30849417	8.5542649
P	15.65562364	6.09576714	6.33611364
B	16.60307539	11.69309857	5.97702522
H	17.70209199	11.29569759	6.13361777
B	15.45025585	11.85015242	7.29005019
H	15.81232819	11.52468236	8.37652499
B	13.85303729	11.44548776	6.68008236
H	13.06824241	10.83262751	7.32590635
B	14.00459414	11.02285348	4.98540999
H	13.3620404	10.16580996	4.49223514
B	14.57206743	12.45614052	4.08314336
H	14.3200695	12.54198452	2.92724544
B	16.19058608	12.87233603	4.70074203
H	17.04637913	13.24474433	3.96883882
B	16.02820289	13.29623364	6.42455179
H	16.78880618	14.05978937	6.93408099
B	14.31062169	13.15470279	6.86956146
H	13.81359523	13.83008373	7.71732687
B	13.41307777	12.62423949	5.42319364
H	12.27322255	12.89844065	5.20821138
B	14.76445909	13.77476568	5.26030695
H	14.60128367	14.9084484	4.92990555
B	17.82101347	9.50511385	4.11333045
H	18.36938798	9.84580307	5.10007737
B	17.74674001	7.82419216	3.61953811
H	18.27728369	7.02636937	4.32055908
B	16.17142232	7.53205003	2.89807701
H	15.57192947	6.52089551	3.08538155
B	15.25905139	9.02957683	2.93071524
H	14.09517601	9.06808693	3.11633389
B	16.10156743	10.2422882	1.92530967
H	15.47303305	11.13064293	1.45320178
B	16.03378037	8.53008543	1.43038068
H	15.35151433	8.19113982	0.51347585
B	17.58995094	7.77323593	1.84801716
H	18.04839981	6.86889236	1.22027561
B	18.61265529	9.01270075	2.61822462
H	19.80327437	9.02161977	2.56443472
B	17.69761742	10.5374246	2.66209135

H	18.16144216	11.62870242	2.69435277
B	17.54872068	9.45636923	1.25884024
H	17.97649458	9.7848219	0.19572575
C	15.257256	10.61351766	6.10059872
C	15.67866065	11.23667068	4.58377799
C	16.33697671	8.62816929	4.22294213
C	16.31217381	10.21392518	3.63083582
C	15.57892821	5.61702877	8.14245864
H	15.3053562	4.55600585	8.22787885
H	16.59023284	5.73449785	8.5573958
C	14.59045863	6.51708165	8.87431675
H	14.5912978	6.32733723	9.95704275
H	13.56746987	6.35167369	8.50728497
C	16.52815433	8.53887633	9.58419723
C	16.53892468	8.22514395	10.9557677
H	15.62588933	7.89094845	11.45161237
C	17.71376567	8.35557337	11.69756051
H	17.71192231	8.11336149	12.76152402
C	18.88851274	8.80094336	11.0804166
H	19.80516533	8.90254986	11.66349661
C	18.88463739	9.12013499	9.72102789
H	19.79506954	9.47597137	9.23724282
C	17.70887112	8.99121009	8.9750359
H	17.70058807	9.24774783	7.91326066
C	13.67127949	9.18736468	9.44304604
C	13.94097098	10.24622502	10.32403174
H	14.96929755	10.55010672	10.51581248
C	12.89314246	10.92213484	10.95344997
H	13.11608685	11.7470196	11.63147162
C	11.56881383	10.5515707	10.71036025
H	10.75254689	11.0843361	11.20027533
C	11.29182661	9.50324225	9.82747411
H	10.25942705	9.21547685	9.62400127
C	12.33456322	8.82942861	9.1907552
H	12.09675222	8.03537847	8.48118364
C	14.09886713	5.37022822	5.67361223
C	13.18144533	6.20528304	5.01728525
H	13.42636271	7.2587865	4.86718257
C	11.96811289	5.69193913	4.54936639
H	11.26513929	6.34964298	4.03678327
C	11.66268635	4.34180812	4.73347776
H	10.71687803	3.94013569	4.36630869
C	12.57304246	3.50098042	5.38424692

H	12.33947626	2.44442833	5.52410255
C	13.7847922	4.01035194	5.85318907
H	14.49263642	3.34204695	6.3466601
C	17.02267263	5.03209254	5.72281875
C	16.88493753	4.22135078	4.58586054
H	15.93878556	4.18986281	4.04695643
C	17.96340088	3.45767019	4.13275667
H	17.84551703	2.83509287	3.24477418
C	19.18735286	3.49692422	4.80445721
H	20.0283884	2.90190281	4.44611798
C	19.33536229	4.31253101	5.93045388
H	20.29194895	4.35922862	6.45264754
C	18.26363111	5.08174583	6.38330304
H	18.40791195	5.7366142	7.24437378

Pt(bc)(dppe) [3c].

Atom	X	Y	Z
Pt	-1.57127888	5.16033774	10.05138955
P	-2.65813417	6.17826253	8.29028714
P	-3.55382819	4.00652176	10.30184773
B	-0.19915394	5.39377446	13.07347848
H	-0.53555026	6.52233185	13.05093001
B	1.43513248	4.88067805	13.57614938
H	2.22726566	5.71164345	13.87512623
B	1.90747243	3.50044913	12.55038509
H	3.0191684	3.38950701	12.15116437
B	0.56486637	3.16960204	11.43215197
H	0.7205181	2.81917381	10.31768321
B	-0.85629841	2.71809476	12.35721598
H	-1.66473941	2.03588998	11.81187679
B	0.76138316	2.16857237	12.86209245
H	1.09908719	1.03266379	12.7281856
B	1.29751685	3.21914901	14.20067246
H	2.02924337	2.83599853	15.06089994
B	-0.00299561	4.40241299	14.51291208
H	-0.22503051	4.88692535	15.57957112
B	-1.32038019	4.08245153	13.36395287
H	-2.44830056	4.37401701	13.57057719
B	-0.41532269	2.72613798	14.07865956
H	-0.94079445	1.97899033	14.84646882
B	1.67169504	5.13157743	9.28706958

H	1.53181399	3.98116658	9.07611392
B	3.02908587	5.78316241	10.24756128
H	3.81005371	5.025678	10.72055215
B	2.40545427	7.15708799	11.19700806
H	2.75993654	7.33791764	12.31453437
B	0.67818543	7.34870002	10.80991274
H	-0.13157222	7.6712395	11.60379083
B	0.50935602	7.72305942	9.10459095
H	-0.46256842	8.32416913	8.77372004
B	1.12082258	6.36373357	8.17340595
H	0.61907009	5.9929045	7.16833473
B	2.85626104	6.18405183	8.52131583
H	3.59855627	5.72205318	7.71031981
B	3.3149822	7.44966288	9.69299436
H	4.41151673	7.91710079	9.72956013
B	1.85420035	8.40995699	10.0504317
H	1.87147999	9.56254832	10.35633931
B	2.13746398	7.80776877	8.39663073
H	2.36705123	8.53881158	7.48182934
C	-0.66805607	4.33271648	11.7746308
C	0.95487918	4.79237031	11.9368736
C	0.28159611	6.10891902	9.67234133
C	1.43363806	5.77767054	10.87044881
C	-4.22699635	5.23603316	7.93718111
H	-4.89332875	5.85592062	7.32239418
H	-3.95393616	4.34425771	7.3549954
C	-4.85755584	4.84548562	9.2683929
H	-5.71570715	4.17143446	9.14585044
H	-5.19543279	5.73473931	9.81941418
C	-1.88672755	6.31517159	6.64789203
C	-1.58624128	5.11382599	5.98306734
H	-1.85375216	4.15470897	6.43120123
C	-0.89297621	5.13876213	4.77447213
H	-0.65189416	4.20340806	4.26829827
C	-0.48186094	6.35966678	4.22923447
H	0.07745446	6.37746722	3.29331691
C	-0.77264267	7.55394878	4.89213023
H	-0.43721628	8.50541434	4.47835898
C	-1.47360756	7.53607468	6.09960891
H	-1.66892075	8.46884135	6.62703062
C	-3.29637202	7.82848562	8.75919539
C	-3.02774138	8.32599885	10.04328912
H	-2.38885591	7.75285281	10.71827517

C	-3.56005971	9.55355768	10.44369648
H	-3.34153576	9.9373058	11.44073559
C	-4.35745231	10.29134504	9.56477227
H	-4.76802751	11.25283547	9.87632314
C	-4.62507917	9.80141783	8.28147614
H	-5.24100093	10.37949223	7.5911938
C	-4.09984469	8.57282938	7.87912254
H	-4.3026496	8.20081849	6.87332653
C	-4.367009	3.82408566	11.9188823
C	-4.81062476	4.99846103	12.5510807
H	-4.69979959	5.96571655	12.05677755
C	-5.34502124	4.94040022	13.83672261
H	-5.67927185	5.85527371	14.32690173
C	-5.4245005	3.7142708	14.50560445
H	-5.82522645	3.67155893	15.51911361
C	-4.97560846	2.54773789	13.88302321
H	-5.01993672	1.594201	14.40996441
C	-4.44787929	2.59807028	12.59139371
H	-4.07474203	1.68893443	12.12191278
C	-3.45519229	2.34985941	9.52998875
C	-4.58891744	1.52636373	9.42767858
H	-5.53676871	1.8401564	9.86851242
C	-4.50175934	0.29298135	8.78090097
H	-5.3829744	-0.34603572	8.7113939
C	-3.28578804	-0.12305493	8.22701287
H	-3.21989918	-1.08797183	7.72239682
C	-2.15594223	0.69285299	8.32628615
H	-1.20454036	0.36730795	7.90432006
C	-2.23729503	1.92573687	8.97736674
H	-1.35493851	2.56197533	9.07167467

Pt(tebc)(dtb-bpy) [8], S_0.

Atom	X	Y	Z
Pt	4.14329669	-0.11314597	8.17752421
N	5.32584742	1.26298222	6.87668211
N	2.79800596	1.42017394	7.6811178
B	5.13543457	-3.14947895	7.83832985
H	4.15384929	-3.2653234	7.19906101
B	5.66670342	-4.36940745	9.02497111
H	4.97590482	-5.32405207	9.19776303
B	6.42181697	-3.51920232	10.38780123

H	6.26022134	-3.88525736	11.50583817
B	6.33082191	-1.76890397	10.04089997
H	6.14220168	-0.96809373	10.88312191
B	6.537634	-2.28543989	7.2531303
H	6.47215382	-1.81690112	6.15931922
B	7.26926286	-1.43805053	8.60513207
H	7.74304676	-0.35004557	8.515513
B	8.00153727	-2.96908178	8.02343375
B	7.45637432	-4.28397582	9.14851793
B	6.63117215	-4.0307487	7.57423808
H	6.68478373	-4.81754273	6.6782773
B	7.83365793	-2.64607668	9.77284684
H	8.75809032	-2.42231443	10.49214692
B	2.3445179	-2.78884934	9.11926855
H	2.46360449	-3.30953305	8.07070854
C	2.6388779	-3.76975827	10.50417671
H	2.99015191	-4.77934381	10.30638505
B	3.38652741	-2.95347473	11.81973924
H	4.21711678	-3.5256958	12.43743496
B	3.4929278	-1.26637312	11.26640427
H	4.41610137	-0.63711692	11.64814473
B	1.87117297	-0.63245951	10.97216349
B	1.18082105	-1.60904678	9.64724298
H	0.36821705	-1.21217655	8.86602848
B	1.03678358	-3.29363921	10.1958718
H	0.28100848	-4.11024847	9.77456271
B	1.66839131	-3.39852297	11.84586825
H	1.34761463	-4.29052333	12.56293139
B	2.17854115	-1.77716133	12.32965158
H	2.16567923	-1.43797059	13.4715173
B	0.70081754	-1.96713042	11.34142947
C	5.53875688	-1.5884514	8.49025731
C	5.08478638	-2.8545376	9.55154262
C	3.71824304	-2.56915319	10.16112539
B	2.94620937	-1.14446767	9.5458733
C	9.39465434	-3.00646563	7.22944021
H	9.59615312	-4.04651321	6.92231421
H	9.28052354	-2.4509094	6.28268283
C	10.61740825	-2.46260786	7.98748182
H	10.46842421	-1.41609382	8.29235622
H	11.52910771	-2.50507248	7.37259244
H	10.80734249	-3.03828413	8.90477386
C	8.36319139	-5.56016507	9.48820071

H	9.17945085	-5.61759009	8.74834031
H	8.86365986	-5.39310699	10.45670325
C	7.63371062	-6.91359107	9.52925656
H	6.8534428	-6.92555078	10.3049319
H	8.32664352	-7.74051717	9.74554983
H	7.1430122	-7.133892	8.56963713
C	-0.78296513	-1.68775832	11.87760152
H	-0.87375804	-0.61703542	12.1257046
H	-1.49836578	-1.85541931	11.05521357
C	-1.2196039	-2.51962733	13.09594451
H	-1.2140757	-3.5957281	12.8691194
H	-2.23665547	-2.25576713	13.4227728
H	-0.54437765	-2.36415354	13.95005267
C	1.52252747	0.91219614	11.24375344
H	2.19019659	1.53801169	10.62834757
H	0.50144937	1.12460473	10.88309765
C	1.6340956	1.37679536	12.70659422
H	0.94398399	0.82388665	13.35944366
H	1.4025342	2.44795918	12.80708958
H	2.6478037	1.21592433	13.10017092
C	6.5957804	1.13414126	6.45708495
H	7.05292203	0.16160771	6.61676407
C	7.31628926	2.16412519	5.85617725
H	8.34226703	1.96479636	5.55339424
C	6.72824608	3.41999004	5.67079815
C	5.39136636	3.53518494	6.07674545
H	4.87185753	4.48363446	5.9523983
C	4.71117397	2.45821745	6.64969081
C	3.28381885	2.50904365	7.01384186
C	2.45078612	3.57188448	6.64303758
H	2.88536632	4.40845244	6.10275025
C	1.0834873	3.56507588	6.92618288
C	0.60582959	2.42052883	7.58299841
H	-0.44815371	2.30690828	7.83397779
C	1.46951441	1.39594826	7.93557833
H	1.10108639	0.50849885	8.44098182
C	7.4656836	4.6196745	5.06313754
C	8.91832136	4.27466831	4.69163382
H	9.40681857	5.16189147	4.26591234
H	8.97027604	3.47529982	3.93890591
H	9.50243537	3.96447132	5.56935066
C	7.48188054	5.77368595	6.09377302
H	6.46707239	6.09232478	6.36889365

H	8.00384347	6.64441706	5.6715911
H	8.00376151	5.47397271	7.01299458
C	6.72486978	5.07733298	3.78380476
H	5.69050871	5.38122863	3.99481827
H	6.6966516	4.27293016	3.0354054
H	7.24320391	5.93988479	3.34074709
C	0.13857991	4.71161778	6.55101006
C	-0.99033389	4.16380762	5.6460413
H	-0.58226377	3.74054513	4.71735545
H	-1.68059644	4.9765739	5.37829536
H	-1.57448127	3.38108418	6.14782413
C	0.86342418	5.84241533	5.80024886
H	1.30226005	5.49139934	4.85498544
H	1.65778185	6.29607517	6.41031951
H	0.14527015	6.63648808	5.5547618
C	-0.47431723	5.2922673	7.84851641
H	-1.1681065	6.10853315	7.60117468
H	0.30687444	5.69411166	8.50884989
H	-1.03440922	4.53284224	8.41014041

Pt(tebc)(dtb-bpy) [8], T_1.

Atom	X	Y	Z
Pt	4.09257795	-0.41049265	7.66548585
N	5.14749894	1.45559988	7.6747957
N	2.69019873	0.93243385	6.91998323
B	4.74029355	-3.48223152	7.79531362
H	3.62177445	-3.54995942	7.4298175
B	5.41095738	-4.52913812	9.07134763
H	4.68955734	-5.31844252	9.59531486
B	6.56678328	-3.57042321	10.02324381
H	6.64633486	-3.69968704	11.2015399
B	6.57865735	-1.92438513	9.34015385
H	6.67445	-0.96567597	10.02075958
B	6.0349581	-2.95903283	6.73945483
H	5.75206625	-2.66383426	5.62116414
B	7.1578204	-1.99627723	7.68311941
H	7.67802397	-1.02770739	7.22553867
B	7.5629146	-3.70466938	7.29969971
B	7.17610547	-4.69430809	8.77354242
B	6.02109657	-4.6110659	7.40196689
H	5.77002756	-5.5504578	6.71066529

B	7.86804402	-3.04421982	8.93577023
H	8.95900507	-2.8399948	9.37137808
B	2.37717803	-2.53231402	9.57158608
H	2.20707831	-3.2782388	8.67689277
C	2.92534188	-3.20830939	11.05396109
H	3.13191729	-4.2759762	11.04225913
B	4.0370323	-2.22956421	11.93644779
H	4.94198037	-2.76044151	12.48339015
B	4.14122769	-0.73701847	10.97913103
H	5.17705478	-0.16973952	10.96350991
B	2.54444193	0.03579286	10.8833219
B	1.47262993	-1.10673088	10.01844975
H	0.5635391	-0.75129056	9.33249369
B	1.33401964	-2.59489663	10.98903146
H	0.44284704	-3.38158184	10.95768421
B	2.33989357	-2.41838313	12.43476469
H	2.12784752	-3.07838942	13.39958085
B	3.08362256	-0.81177767	12.38709816
H	3.37108384	-0.23002272	13.38612331
B	1.39457667	-1.01683278	11.81936654
C	5.4607932	-1.91965792	8.00571109
C	5.14017016	-2.87378769	9.38507435
C	3.99236498	-2.27634558	10.20312462
B	3.18616534	-0.93688915	9.48549965
C	8.70433265	-4.09582423	6.24628564
H	8.72400679	-5.19351615	6.13915376
H	8.40987391	-3.71896826	5.25238875
C	10.12312353	-3.59858823	6.57100287
H	10.15362835	-2.50260794	6.66048496
H	10.84135029	-3.88836492	5.78935323
H	10.48406957	-4.01035889	7.52437541
C	7.99625593	-6.01318739	9.16417295
H	8.4851663	-6.40306375	8.25573169
H	8.8223815	-5.7304672	9.83770762
C	7.18459027	-7.14465637	9.81679072
H	6.72163517	-6.81518785	10.75879303
H	7.81778519	-8.0142123	10.04724125
H	6.37422346	-7.49005549	9.15794758
C	0.12003817	-0.42530611	12.58593652
H	0.29317101	0.64463393	12.78780191
H	-0.74819354	-0.45432062	11.9068172
C	-0.25288855	-1.12661766	13.90399468
H	-0.48687291	-2.18896495	13.74252754

H	-1.13336951	-0.66105894	14.37127682
H	0.57162272	-1.08089951	14.6305081
C	2.37057994	1.62462421	10.77652875
H	2.79233562	1.96144055	9.81559765
H	1.29406214	1.85504863	10.7144098
C	2.99731816	2.44955378	11.91342532
H	2.55740419	2.193519	12.88772985
H	2.84974553	3.5282108	11.75507613
H	4.07941216	2.26958558	11.99115948
C	6.42717122	1.63901721	8.073698
H	6.9234034	0.7664898	8.49643561
C	7.07771077	2.85154823	7.95902844
H	8.10277815	2.91957319	8.31554165
C	6.41097013	3.9696347	7.39032302
C	5.09556894	3.76818693	6.98879752
H	4.53536276	4.58485464	6.53420748
C	4.44514927	2.5281226	7.14381159
C	3.07915588	2.25527765	6.78388023
C	2.16037142	3.21601152	6.30601655
H	2.49529057	4.24730378	6.22049272
C	0.86228603	2.877975	5.95821582
C	0.49538267	1.51075124	6.12643231
H	-0.50485732	1.15718718	5.88151009
C	1.41001535	0.60147137	6.60520921
H	1.14758109	-0.44624826	6.7480564
C	7.08715112	5.33649607	7.20529711
C	8.51541341	5.35567049	7.780892
H	8.95514504	6.35194141	7.63487322
H	9.16945654	4.62923635	7.27860871
H	8.52397038	5.14342007	8.8595934
C	6.25826314	6.42535806	7.92650233
H	5.23908506	6.49900534	7.52314964
H	6.73755592	7.40747721	7.80266659
H	6.18175806	6.21353243	9.00222096
C	7.16463962	5.66413866	5.69492606
H	6.16762306	5.69836185	5.23454944
H	7.75812377	4.90998019	5.15939722
H	7.63956662	6.64488118	5.54486437
C	-0.16051993	3.88735754	5.42747164
C	-0.65045365	3.42644724	4.03325239
H	0.18481359	3.37567806	3.32059216
H	-1.393769	4.13640099	3.64193077
H	-1.12224741	2.43542155	4.07226173

C	0.43073729	5.30106295	5.29060198
H	1.27998056	5.32191102	4.59230782
H	0.7639283	5.70148465	6.25899983
H	-0.3365598	5.98297002	4.89830704
C	-1.36266771	3.94776335	6.40004121
H	-2.12034048	4.64894924	6.02011562
H	-1.04562881	4.28900713	7.39559373
H	-1.84141649	2.96624314	6.51677651

References

1. A. B. Pangborn, M. A. Giardello, R. H. Grubbs, R. K. Rosen, F. J. Timmers, *Organometallics* **1996**, *15*, 1518-1520.
2. D. Drew, J. R. Doyle, A. G. Shaver, *Inorg. Synth.* **1990**, *28*, 346-349.
3. McDermott, J. X., White, J. F., Whitesides, G. M., *J. Am. Chem. Soc.* **1976**, *98*, 6521-6528.
4. C. F. Guerra, J. G. Snijders, G. te Velde, E. J. Baerends, *Theo. Chem. Acc.* **1998**, *99*, 391-403; G. te Velde, F. M. Bickelhaupt, E. J. Baerends C. F. Guerra, S. J. A. Van Gisbergen, J. G. Snijders, T. Ziegler, *J. Comput. Chem.* **2001**, *22*, 931-967; ADF 2014/GUI 2014, SCM, Amsterdam, The Netherlands, http://www.scm.com, E.J. Baerends, T. Ziegler, J. Autschbach, D. Bashford, A. Bérces, F.M. Bickelhaupt, C. Bo, P.M. Boerrigter, L. Cavallo, D.P. Chong, L. Deng, R.M. Dickson, D.E. Ellis, M. van Faassen, L. Fan, T.H. Fischer, C. Fonseca Guerra, M. Franchini, A. Ghysels, A. Giammona, S.J.A. van Gisbergen, A.W. Götz, J.A. Groeneveld, O.V. Gritsenko, M. Grüning, S. Gusarov, F.E. Harris, P. van den Hoek, C.R. Jacob, H. Jacobsen, L. Jensen, J.W. Kaminski, G. van Kessel, F. Kootstra, A. Kovalenko, M.V. Krykunov, E. van Lenthe, D.A. McCormack, A. Michalak, M. Mitoraj, S.M. Morton, J. Neugebauer, V.P. Nicu, L. Noodleman, V.P. Osinga, S. Patchkovskii, M. Pavanello, P.H.T. Philipsen, D. Post, C.C. Pye, W. Ravenek, J.I. Rodríguez, P. Ros, P.R.T. Schipper, H. van Schoot, G. Schreckenbach, J.S. Seldenthuis, M. Seth, J.G. Snijders, M. Solà, M. Swart, D. Swerhone, G. te Velde, P. Vernooijs, L. Versluis, L. Visscher, O. Visser, F. Wang, T.A. Wesolowski, E.M. van Wezenbeek, G. Wiesenekker, S.K. Wolff, T.K. Woo, A.L. Yakovlev.
5. A. D. Becke, *Phys. Rev. A* **1988**, *38*, 3098-3100.
6. J. P. Perdew, *Phys. Rev. B* **1986**, *33*, 8822-8824.
7. S. Grimme, J. Antony, S. Ehrlich, H. Krieg, *J. Chem. Phys.* **2010**, *132*, 154104.
8. Ren, S., Xie, Z., *Organometallics* **2008**, *27,* 5167–5168.
9. Zheng, Z., Jiang, W., Zinn, A. A., Knobler, C. B., Hawthorne, M. F., *Inorg. Chem.* **1995***, 34,* 2095-2100.
10. G. T. L. Broadwood-Strong, P. A. Chaloner, P. B. Hitchcock, *Polyhedron* **1993**, *12*, 721-729.
11. J. Yang, D. K. Kersi, L. J. Giles, B. W. Stein, C. Feng, C. R. Tichnell, D. A. Shultz, M. L. Kirk, *Inorg. Chem.* **2014**, *53*, 4791-4793.
12. M. J. Martin, W. Y. Man, G. M. Rosair, A. J. Welch, *J. Organomet. Chem.* **2015**, *798*, 36-40.

13. H. J. Bae, H. Kim, K. M. Lee, T. Kim, M. Eo, Y. S. Lee, Y. Do, M. H. Lee, *Dalton Trans.* **2013**, *42*, 8549-8552
14. T. Kim, J. Lee, S. U. Lee, M. H. Lee, *Organometallics* **2015**, *34*, 3455–3458
15. C. Cornioley-Deuschel, A. von Zelewsky, *Inorg. Chem.* **1987**, *26*, 3354-3358
16. C. Shi, H. Sun, Q. Jiang, J. Wang, W. Huang, H. Yan, *Chem. Commun.* **2013**, *49*, 4746-4748.
17. C. Shi, H. Sun, X. Tang, W. Lv, H. Yan, Q. Zhao, J. Wang, W. Huang, *Angew. Chem. Int. Ed.* **2013**, *52*, 13434-13438.
18. T. Kim, H. Kim, K. M. Lee, Y. S. Lee, M. H. Lee, *Inorg. Chem.* **2013**, *52*, 160-168.
19. H. J. Bae, J. Chung, H. Kim, J. Park, K. M. Lee, T.-W. Koh, Y. S. Lee, S. Yoo, Y. Do, M. H. Lee, *Inorg. Chem.* **2014**, *53*, 128-138.
20. For crystal structures of corresponding M(bph)(P^P) complexes, where P^P = chelated diphosphine ligand, see: a) B. L. Edelbach, D. A. Vicic, R. J. Lachicotte, W. D. Jones, *Organometallics* **1998**, *17*, 4784-4794; b) D. A. Vicic, W. D. Jones, *J. Am. Chem. Soc.* **1999**, *121*, 7606-7617.
21. P. Pyykkö, *Chem. Rev.* **1988**, *88*, 563-594.
22. P. Sgarbossa, E. Pizzo, A. Scarso, S. M. Sbovata, R. A. Michelin, M. Mozzon, G. Strukul, F. Benetollo, *J. Organomet. Chem.* **2006**, *691*, 3659-3666.
23. G. S. Kazakov, I. B. Sivaev, K. Yu. Suponitsky, A. D. Kirilin, V. I. Bregadze, A. J. Welch, *J. Organomet. Chem.* **2016**, *805*, 1-5.
24. M. J. Petersson, W. A. Loughlin, I. D. Jenkins, *Chem. Commun.* **2008**, *37*, 4493-4494.

1H NMR

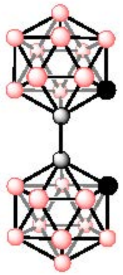

2

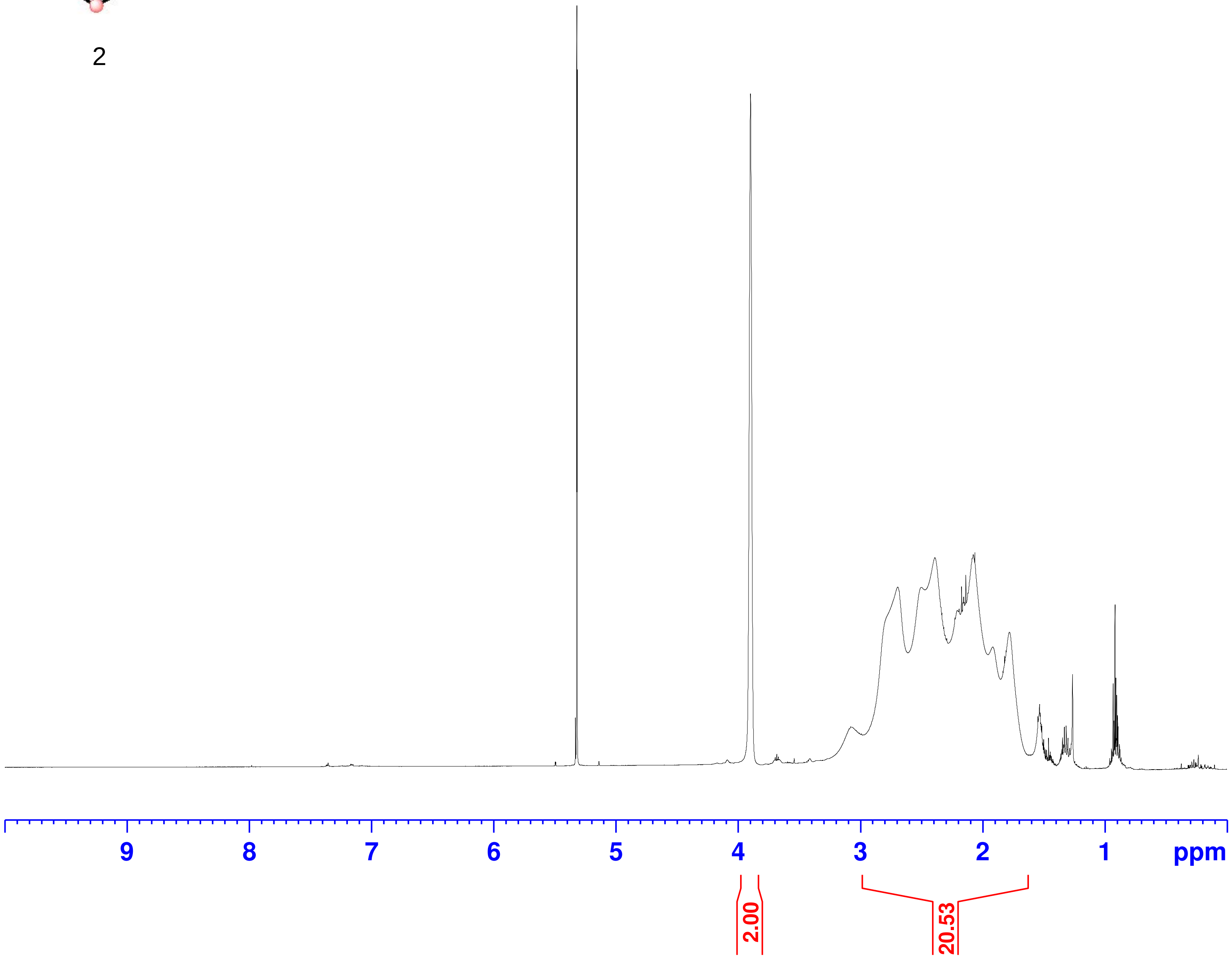

Current Data Parameters
NAME bis-carborane
EXPNO 10
PROCNO 1

F2 - Acquisition Parameters
Date_ 20150918
Time 16.05
INSTRUM av500
PROBHD 5 mm DCH 13C-1
PULPROG zg30
TD 65536
SOLVENT CD2Cl2
NS 24
DS 0
SWH 10000.000 Hz
FIDRES 0.152588 Hz
AQ 3.2767999 sec
RG 23.34
DW 50.000 usec
DE 10.00 usec
TE 298.0 K
D1 2.00000000 sec
TD0 1

======== CHANNEL f1 ========
SFO1 500.1330008 MHz
NUC1 1H
P1 10.00 usec
PLW1 13.50000000 W

F2 - Processing parameters
SI 65536
SF 500.1300194 MHz
WDW EM
SSB 0
LB 0.30 Hz
GB 0
PC 1.00

11B NMR

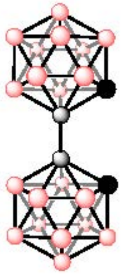

2

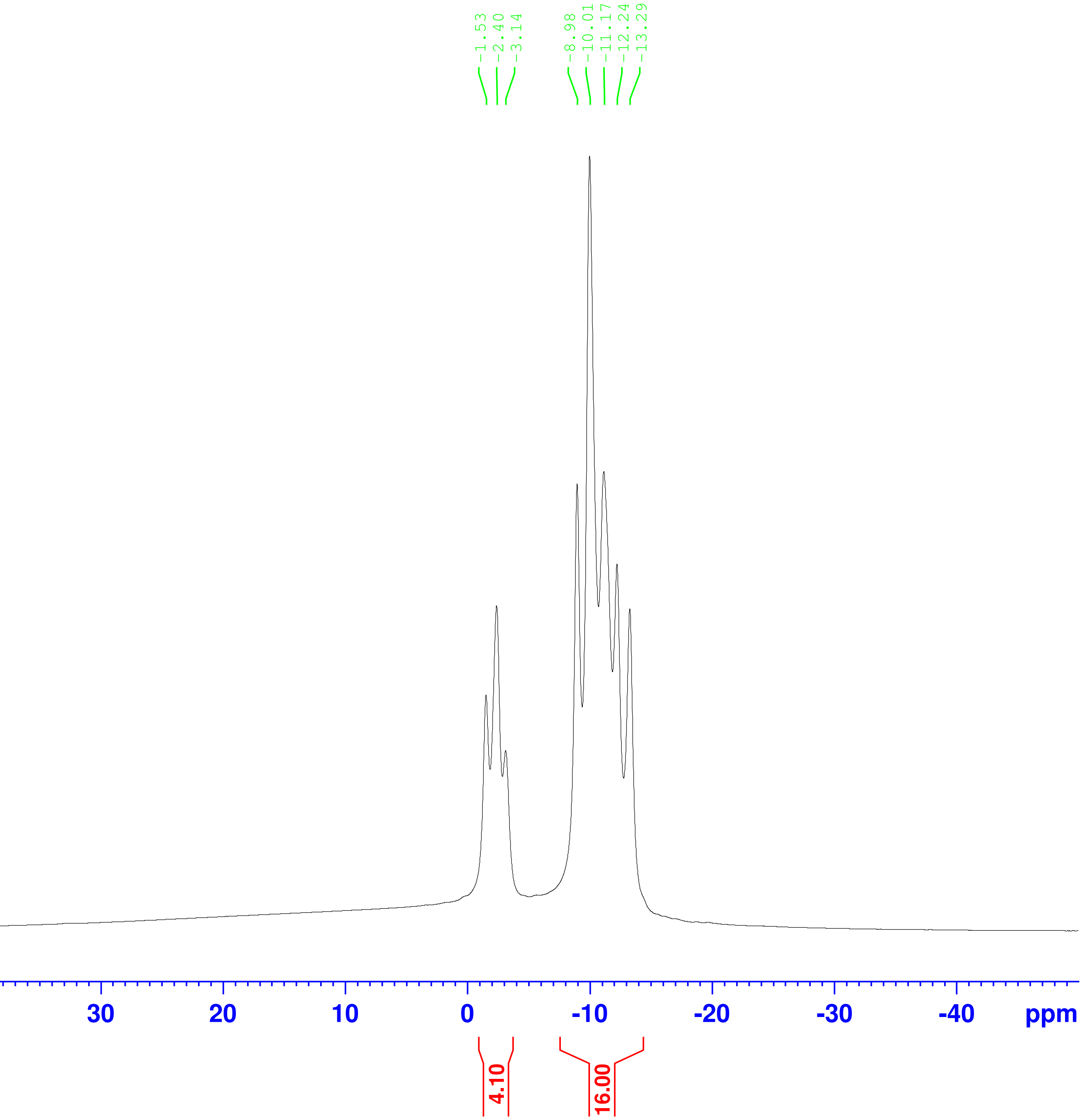

Current Data Parameters
NAME bis-carborane 11B
EXPNO 3
PROCNO 1

F2 - Acquisition Parameters
Date_ 20160114
Time 12.31
INSTRUM drx500
PROBHD 5 mm bb-Z Z800
PULPROG zgdc
TD 7168
SOLVENT D2O
NS 4096
DS 4
SWH 64102.562 Hz
FIDRES 8.942880 Hz
AQ 0.0559104 sec
RG 362
DW 7.800 usec
DE 6.00 usec
TE 296.8 K
D1 0.00000400 sec
d11 0.03000000 sec
TD0 1

======== CHANNEL f1 ========
NUC1 11B
P1 8.00 usec
PL1 -2.00 dB
SFO1 160.5257140 MHz

======== CHANNEL f2 ========
CPDPRG[2 waltz16
NUC2 off
PCPD2 100.00 usec
PL2 0 dB
PL12 17.52 dB
SFO2 500.3300000 MHz

F2 - Processing parameters
SI 32768
SF 160.5257924 MHz
WDW EM
SSB 0
LB 10.00 Hz
GB 0
PC 1.40

11B{1H} NMR

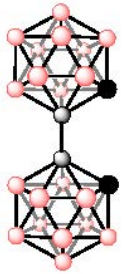

2

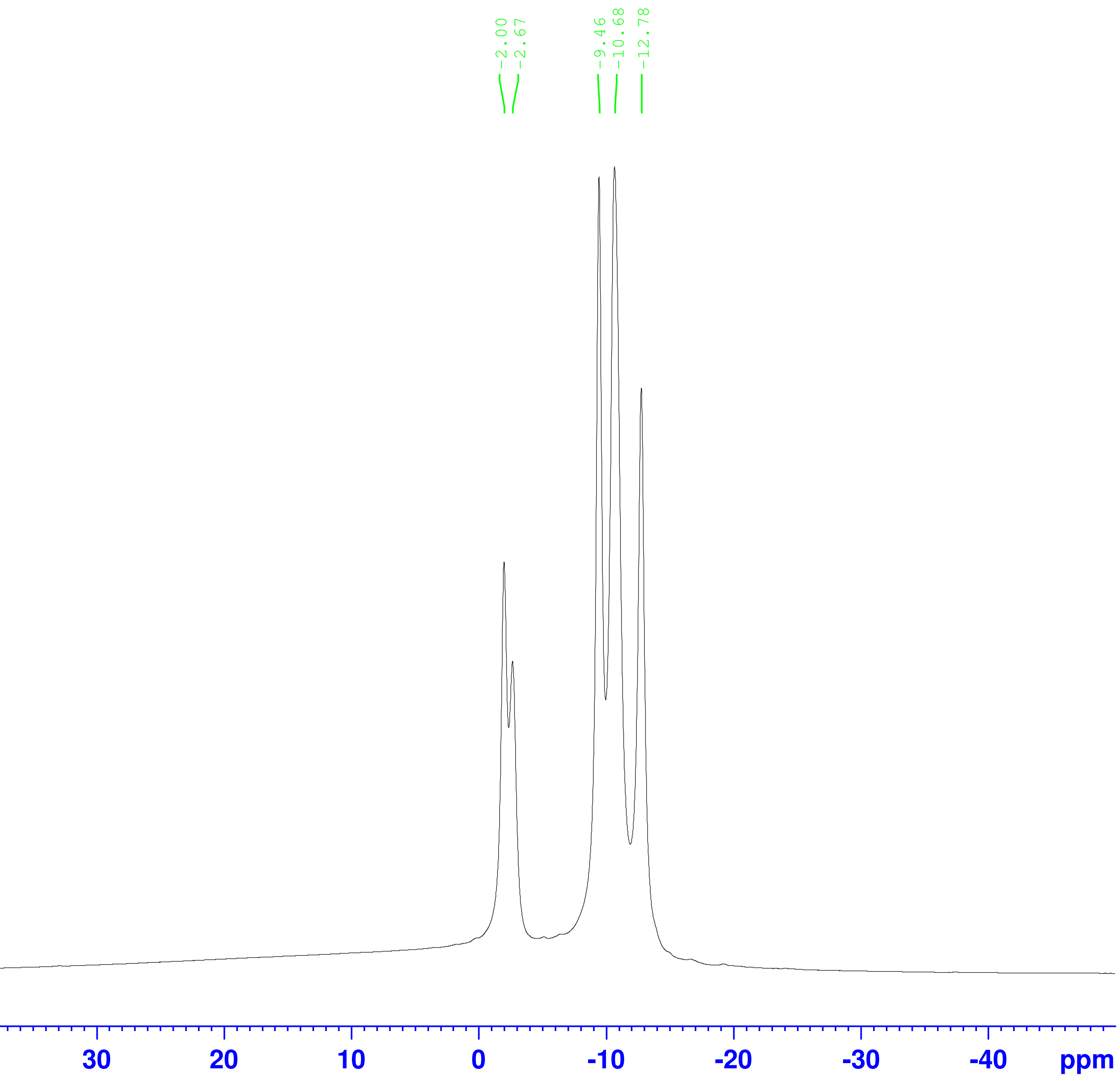

BRUKER

Current Data Parameters
NAME bis-carborane 11B
EXPNO 2
PROCNO 1

F2 - Acquisition Parameters
Date_ 20160114
Time 12.22
INSTRUM drx500
PROBHD 5 mm bb-Z Z800
PULPROG zgdc
TD 7168
SOLVENT D2O
NS 4096
DS 4
SWH 64102.562 Hz
FIDRES 8.942880 Hz
AQ 0.0559104 sec
RG 362
DW 7.800 usec
DE 6.00 usec
TE 297.1 K
D1 0.00000400 sec
d11 0.03000000 sec
TD0 1

======== CHANNEL f1 ========
NUC1 11B
P1 8.00 usec
PL1 -2.00 dB
SFO1 160.5257136 MHz

======== CHANNEL f2 ========
CPDPRG[2 waltz16
NUC2 1H
PCPD2 100.00 usec
PL2 0 dB
PL12 17.52 dB
SFO2 500.3330020 MHz

F2 - Processing parameters
SI 32768
SF 160.5257924 MHz
WDW EM
SSB 0
LB 10.00 Hz
GB 0
PC 1.40

13C{1H} NMR

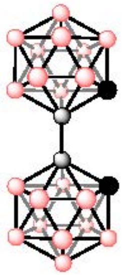

2

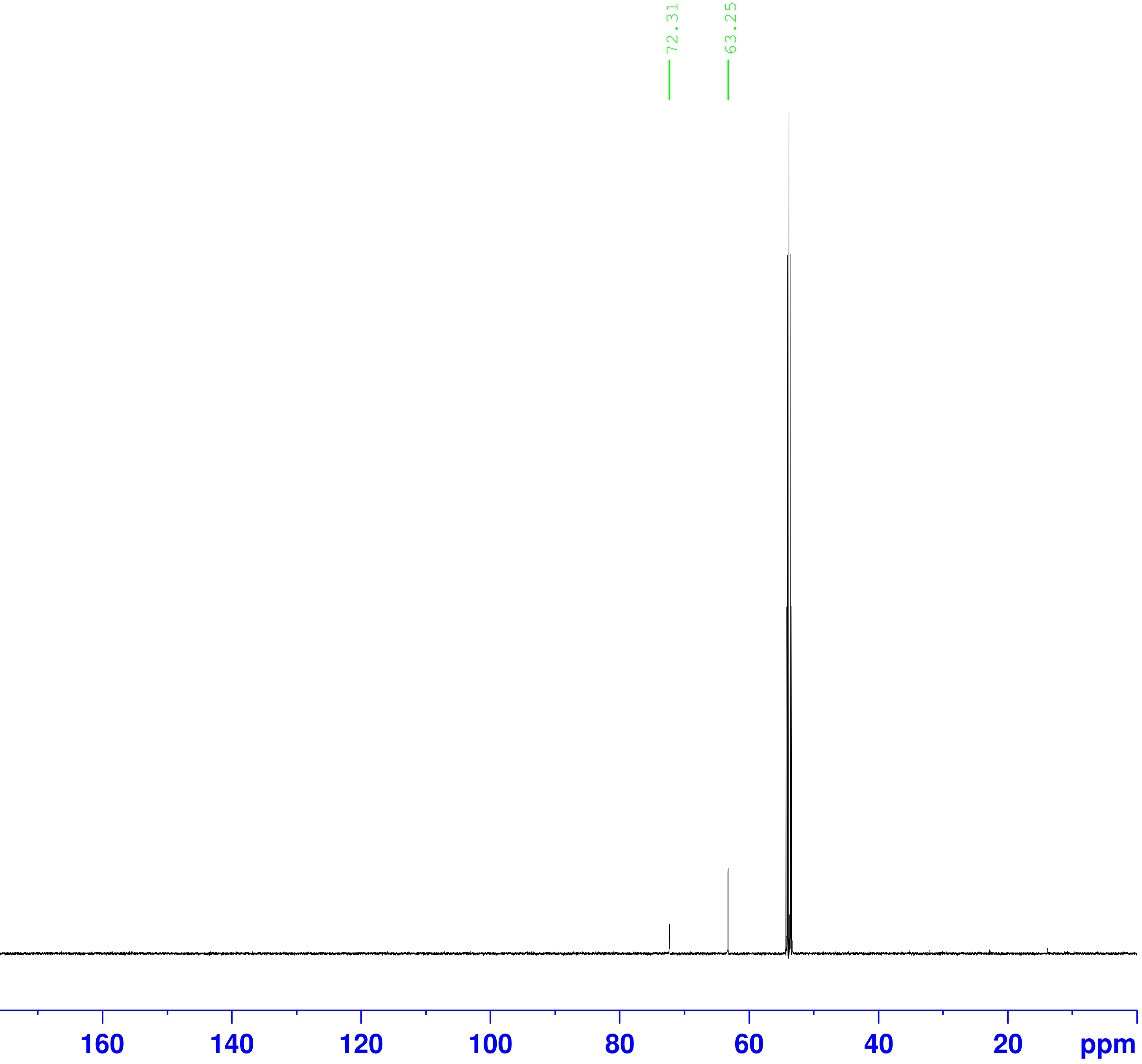

Current Data Parameters
NAME bis-carborane
EXPNO 11
PROCNO 1

F2 - Acquisition Parameters
Date_ 20150918
Time 16.08
INSTRUM av500
PROBHD 5 mm DCH 13C-1
PULPROG zgpg30
TD 65536
SOLVENT CD2Cl2
NS 32
DS 2
SWH 31250.000 Hz
FIDRES 0.476837 Hz
AQ 1.0485760 sec
RG 204.54
DW 16.000 usec
DE 18.00 usec
TE 298.0 K
D1 2.00000000 sec
D11 0.03000000 sec
TD0 1

======== CHANNEL f1 ========
SFO1 125.7722511 MHz
NUC1 13C
P1 9.63 usec
PLW1 23.00000000 W

======== CHANNEL f2 ========
SFO2 500.1330008 MHz
NUC2 1H
CPDPRG[2 waltz16
PCPD2 80.00 usec
PLW2 13.50000000 W
PLW12 0.21094000 W
PLW13 0.13500001 W

F2 - Processing parameters
SI 131072
SF 125.7577396 MHz
WDW EM
SSB 0
LB 1.00 Hz
GB 0
PC 1.40

1H NMR

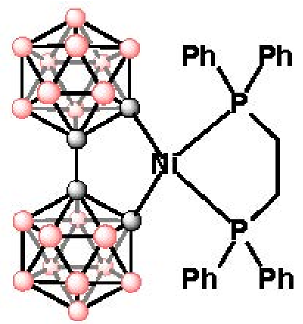

3a

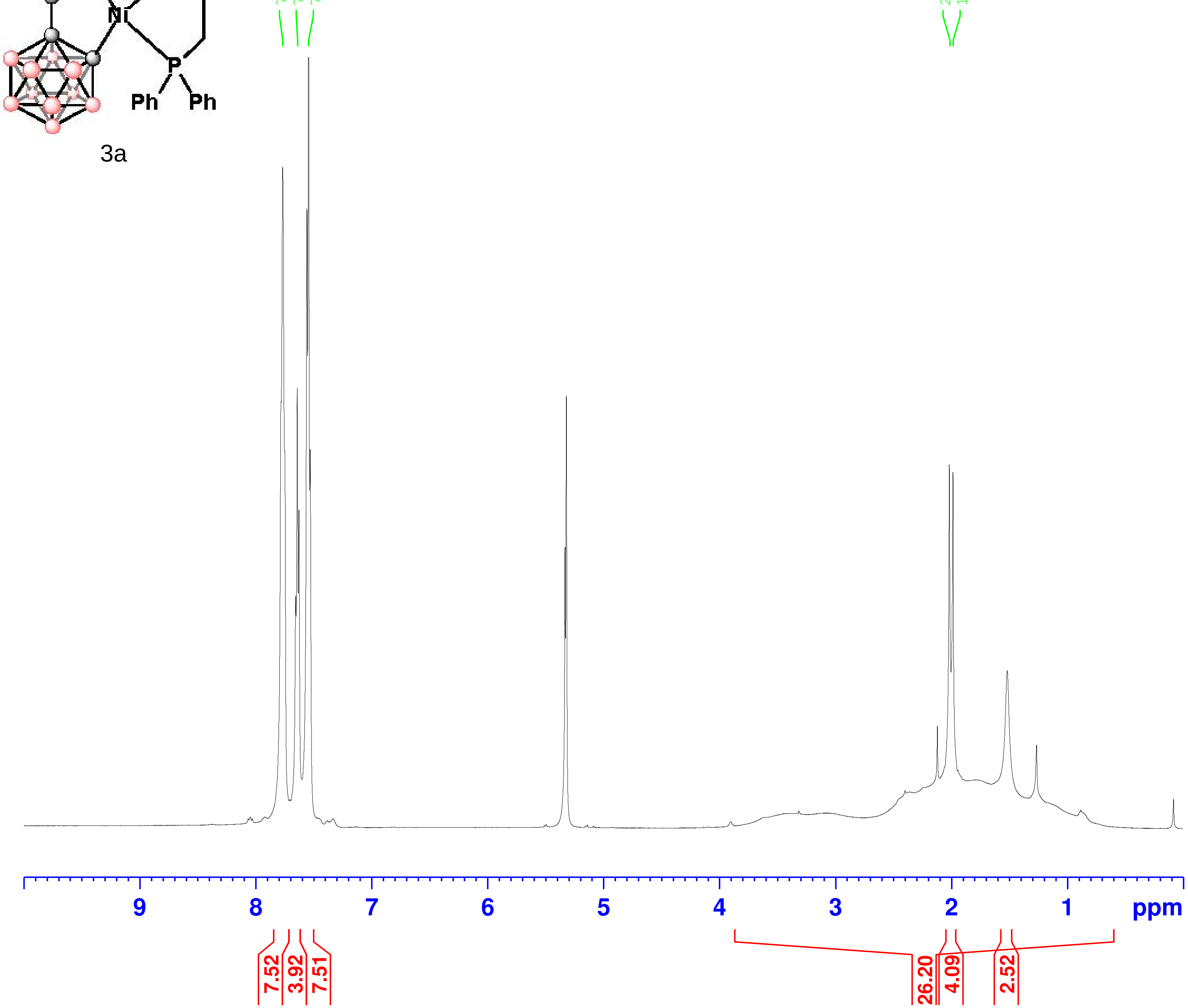


```
Current Data Parameters
NAME      kk-1-235 ni(bc)(dppe) pure
EXPNO                        10
PROCNO                        1

F2 - Acquisition Parameters
Date_                  20150920
Time                      16.43
INSTRUM                   av500
PROBHD    5 mm DCH 13C-1
PULPROG                    zg30
TD                        65536
SOLVENT                 CD2Cl2
NS                           64
DS                            0
SWH               10000.000 Hz
FIDRES             0.152588 Hz
AQ                3.2767999 sec
RG                        12.14
DW                   50.000 usec
DE                    10.00 usec
TE                      298.0 K
D1               2.00000000 sec
TD0                           1

======== CHANNEL f1 ========
SFO1           500.1330008 MHz
NUC1                         1H
P1                    10.00 usec
PLW1            13.50000000 W

F2 - Processing parameters
SI                        65536
SF             500.1300193 MHz
WDW                          EM
SSB       0
LB                      0.30 Hz
GB        0
PC                         1.00
```

11B NMR

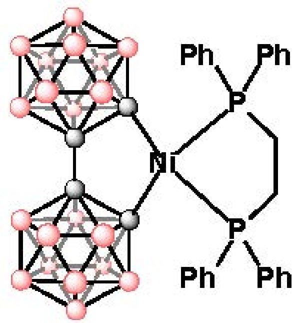

3a

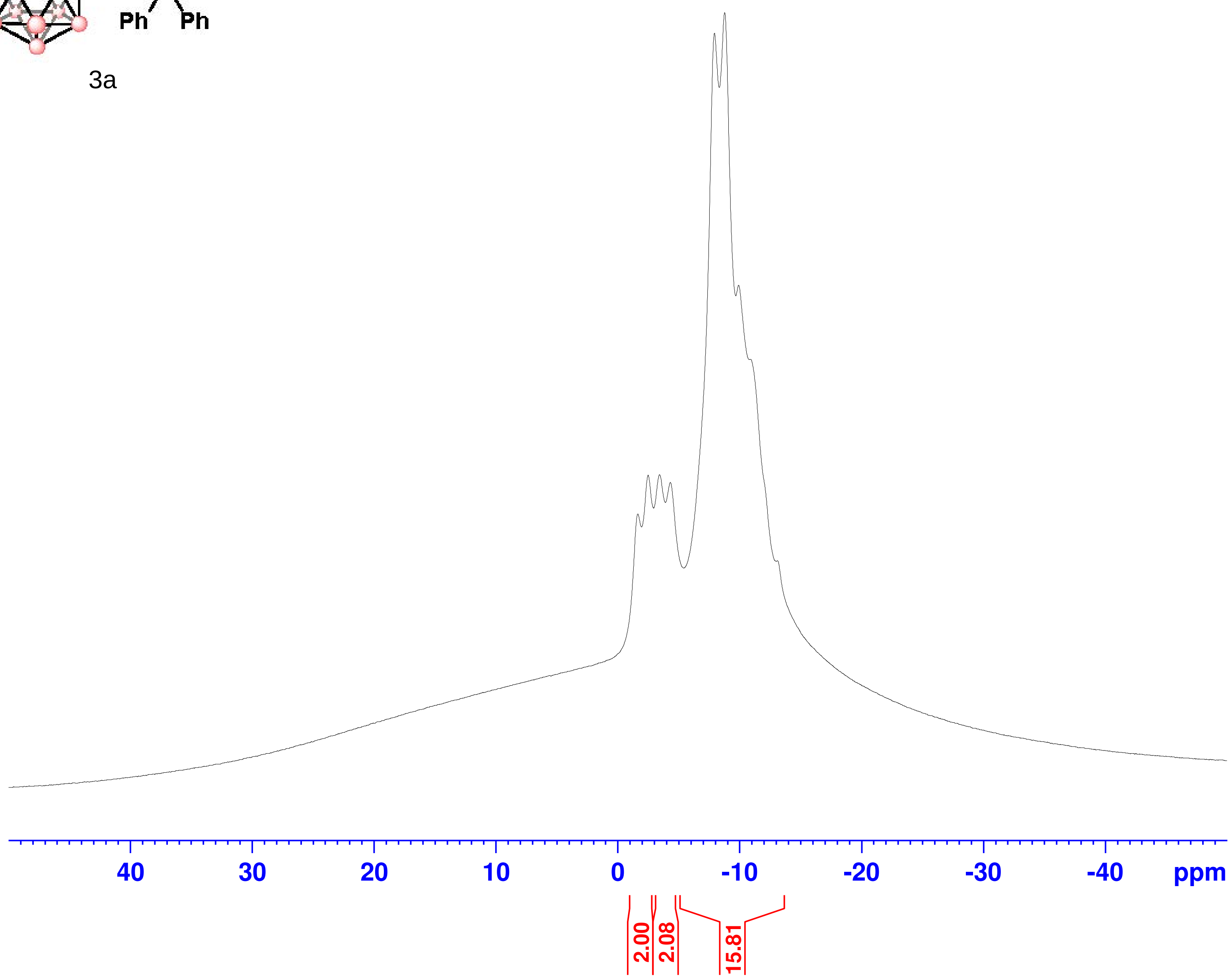


```
Current Data Parameters
NAME      kk-1-235 ni(bc)(dppe) pure 11b
EXPNO                   2
PROCNO                  1

F2 - Acquisition Parameters
Date_            20150922
Time                14.35
INSTRUM            drx500
PROBHD    5 mm bb-Z Z800
PULPROG              zgdc
TD                   7168
SOLVENT            CD2Cl2
NS                   4096
DS                      4
SWH             64102.562 Hz
FIDRES           8.942880 Hz
AQ              0.0559104 sec
RG                  574.7
DW                  7.800 usec
DE                   6.00 usec
TE                  296.8 K
D1             0.00000400 sec
d11            0.03000000 sec
TD0                     1

======== CHANNEL f1 ========
NUC1                  11B
P1                   8.00 usec
PL1                 -2.00 dB
SFO1          160.5257140 MHz

======== CHANNEL f2 ========
CPDPRG[2          waltz16
NUC2                  off
PCPD2              100.00 usec
PL2       0 dB
PL12                17.52 dB
SFO2          500.3300000 MHz

F2 - Processing parameters
SI                  32768
SF            160.5257717 MHz
WDW                    EM
SSB       0
LB                  10.00 Hz
GB        0
PC                   1.40
```

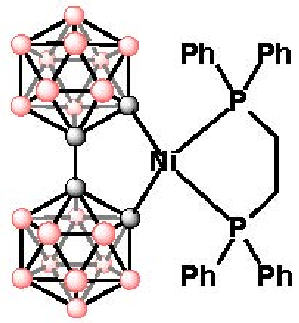

3a

11B{1H} NMR

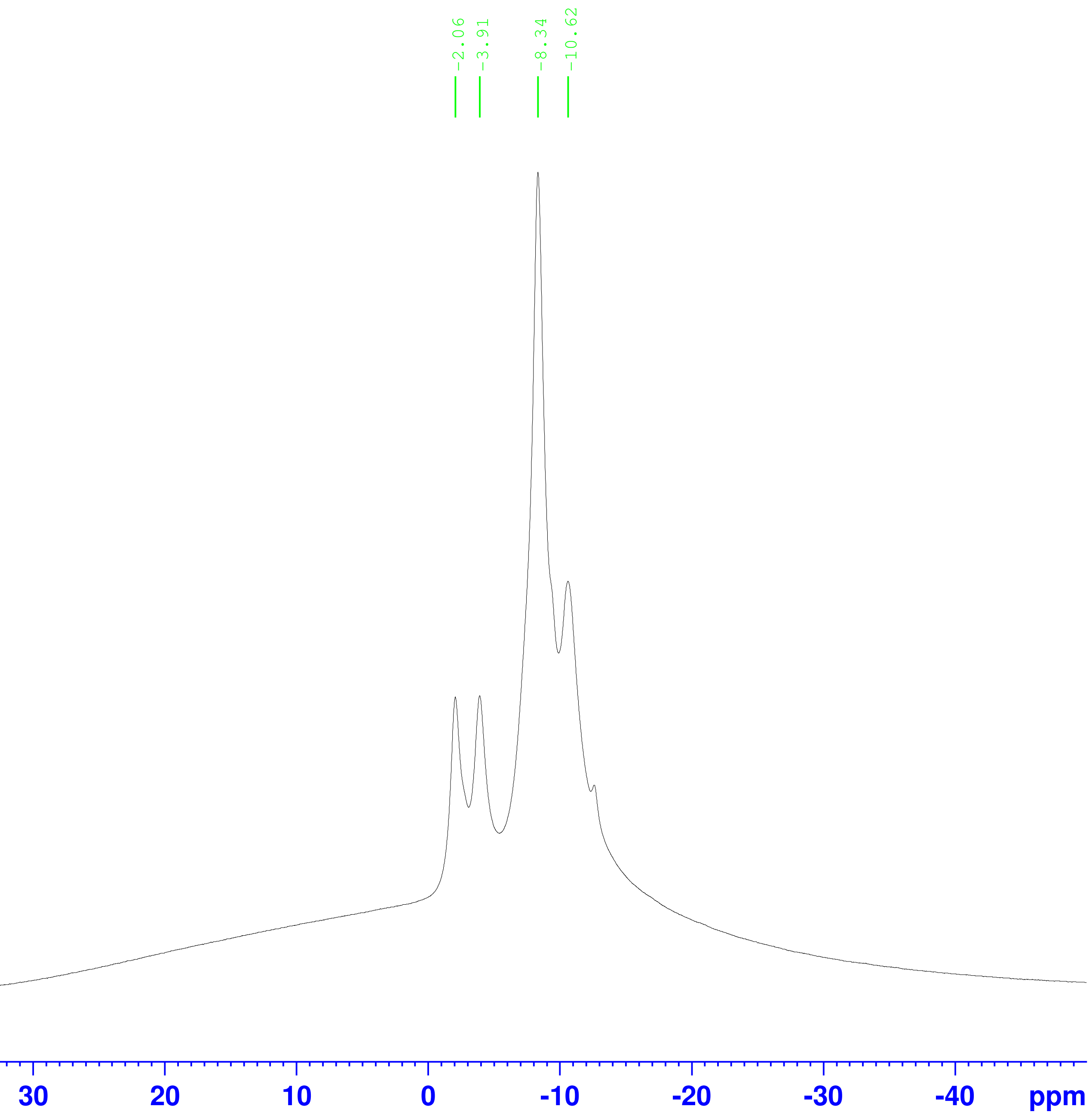


```
Current Data Parameters
NAME      Ni(bc)(dppe) pure 11B
EXPNO                    1
PROCNO                   1

F2 - Acquisition Parameters
Date_              20150922
Time                  13.40
INSTRUM              drx500
PROBHD    5 mm bb-Z Z800
PULPROG                zgdc
TD                     7168
SOLVENT              CD2Cl2
NS                     4096
DS                        4
SWH              64102.562 Hz
FIDRES            8.942880 Hz
AQ               0.0559104 sec
RG                    574.7
DW                    7.800 usec
DE                     6.00 usec
TE                    297.1 K
D1               0.00000400 sec
d11              0.03000000 sec
TD0                       1

======== CHANNEL f1 ========
NUC1                    11B
P1                     8.00 usec
PL1                   -2.00 dB
SFO1            160.5257136 MHz

======== CHANNEL f2 ========
CPDPRG[2            waltz16
NUC2                     1H
PCPD2                100.00 usec
PL2        0 dB
PL12                  17.52 dB
SFO2            500.3330020 MHz

F2 - Processing parameters
SI                    32768
SF              160.5257717 MHz
WDW                      EM
SSB        0
LB                    10.00 Hz
GB         0
PC                     1.40
```

13C{1H} NMR

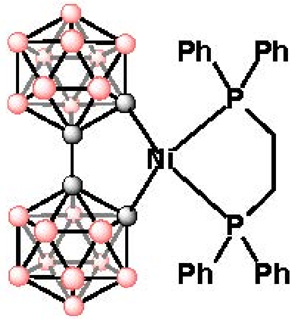

3a

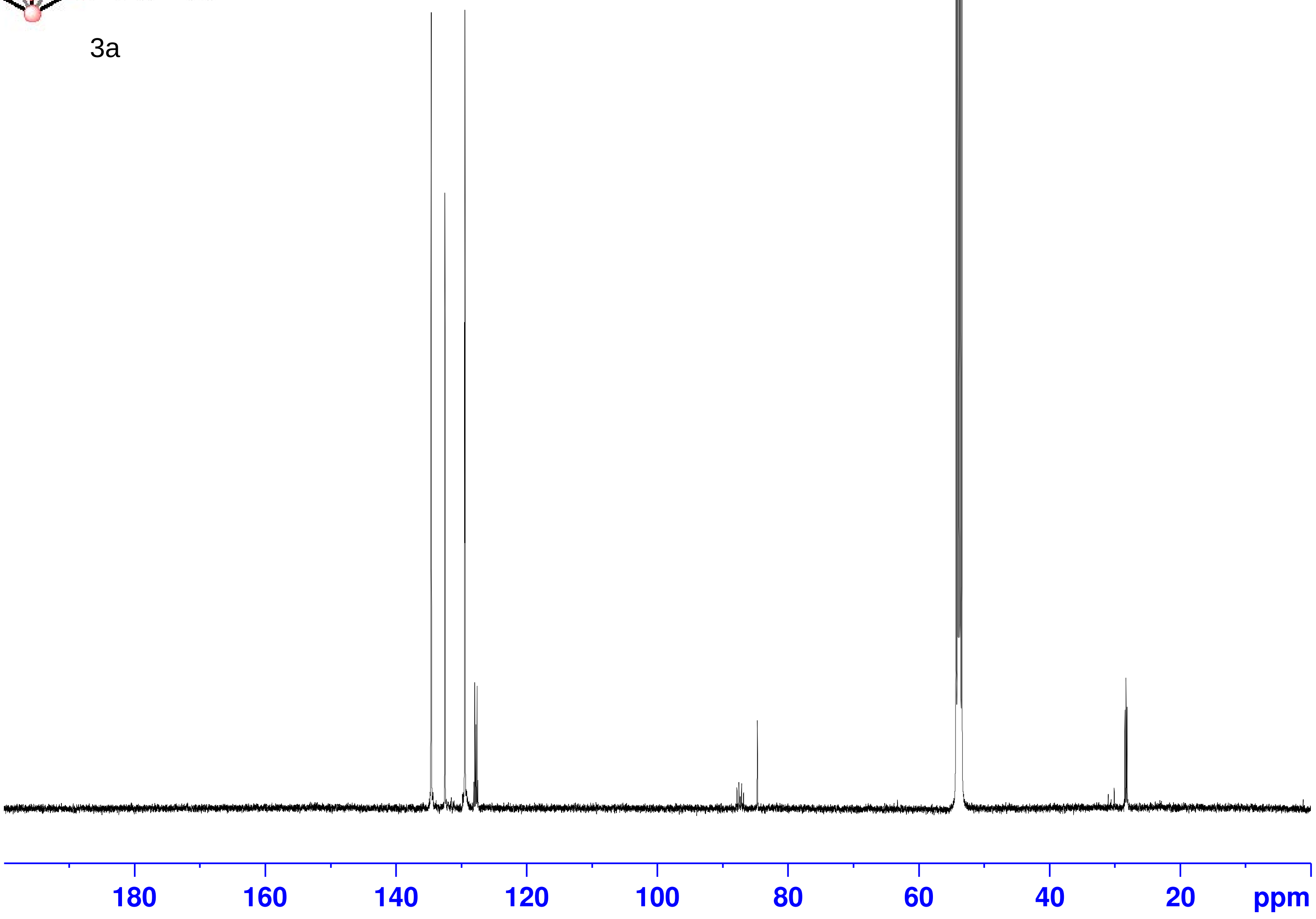


```
Current Data Parameters
NAME      kk-1-235 ni(bc)(dppe) pure
EXPNO                    11
PROCNO                   1

F2 - Acquisition Parameters
Date_              20150920
Time                  17.37
INSTRUM               av500
PROBHD    5 mm DCH 13C-1
PULPROG             zgpg30
TD                    65536
SOLVENT             CD2Cl2
NS                     1024
DS                        2
SWH              31250.000 Hz
FIDRES            0.476837 Hz
AQ               1.0485760 sec
RG                   204.54
DW                   16.000 usec
DE                    18.00 usec
TE                    298.0 K
D1               2.00000000 sec
D11              0.03000000 sec
TD0                       1

======== CHANNEL f1 ========
SFO1         125.7722511 MHz
NUC1                    13C
P1                     9.63 usec
PLW1         23.00000000 W

======== CHANNEL f2 ========
SFO2         500.1330008 MHz
NUC2                     1H
CPDPRG[2            waltz16
PCPD2                 80.00 usec
PLW2         13.50000000 W
PLW12         0.21094000 W
PLW13         0.13500001 W

F2 - Processing parameters
SI                   131072
SF           125.7577378 MHz
WDW                      EM
SSB      0
LB                     1.00 Hz
GB       0
PC                     1.40
```

31P{1H} NMR

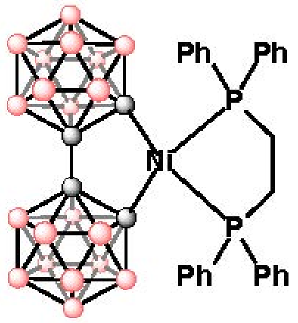

3a

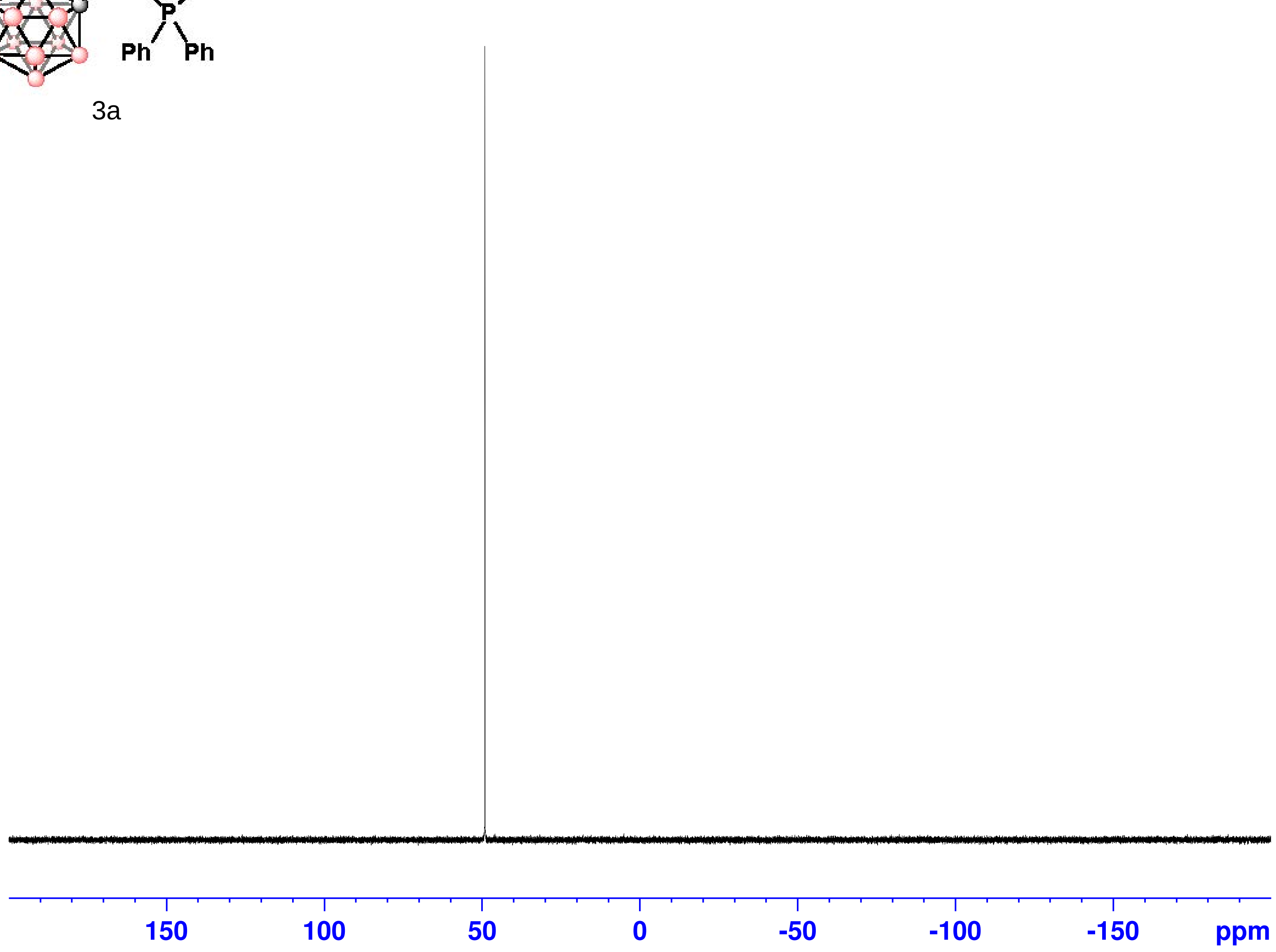


```
Current Data Parameters
NAME      kk-1-235 ni(bc)(dppe) pure, 31p
EXPNO                    1
PROCNO                   1

F2 - Acquisition Parameters
Date_             20150920
Time                 15.34
INSTRUM             drx500
PROBHD    5 mm bb-Z Z800
PULPROG            zgdc30
TD                  131072
SOLVENT             CD2Cl2
NS                     128
DS                       0
SWH             100000.000 Hz
FIDRES            0.762939 Hz
AQ               0.6553600 sec
RG                  9195.2
DW                   5.000 usec
DE                    6.00 usec
TE                   297.1 K
D1              2.00000000 sec
d11             0.03000000 sec
TD0                      1

======== CHANNEL f1 ========
NUC1                   31P
P1                    9.00 usec
PL1       0 dB
SFO1           202.5370460 MHz

======== CHANNEL f2 ========
CPDPRG[2           waltz16
NUC2                    1H
PCPD2               100.00 usec
PL2       0 dB
PL12                 17.52 dB
SFO2           500.3320013 MHz

F2 - Processing parameters
SI                  131072
SF             202.5371980 MHz
WDW                     EM
SSB       0
LB                    1.00 Hz
GB        0
PC                    1.40
```

1H NMR

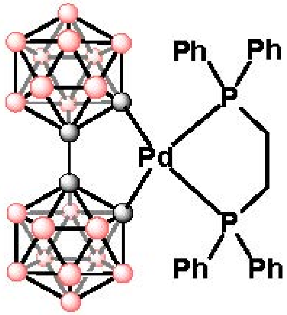

3b

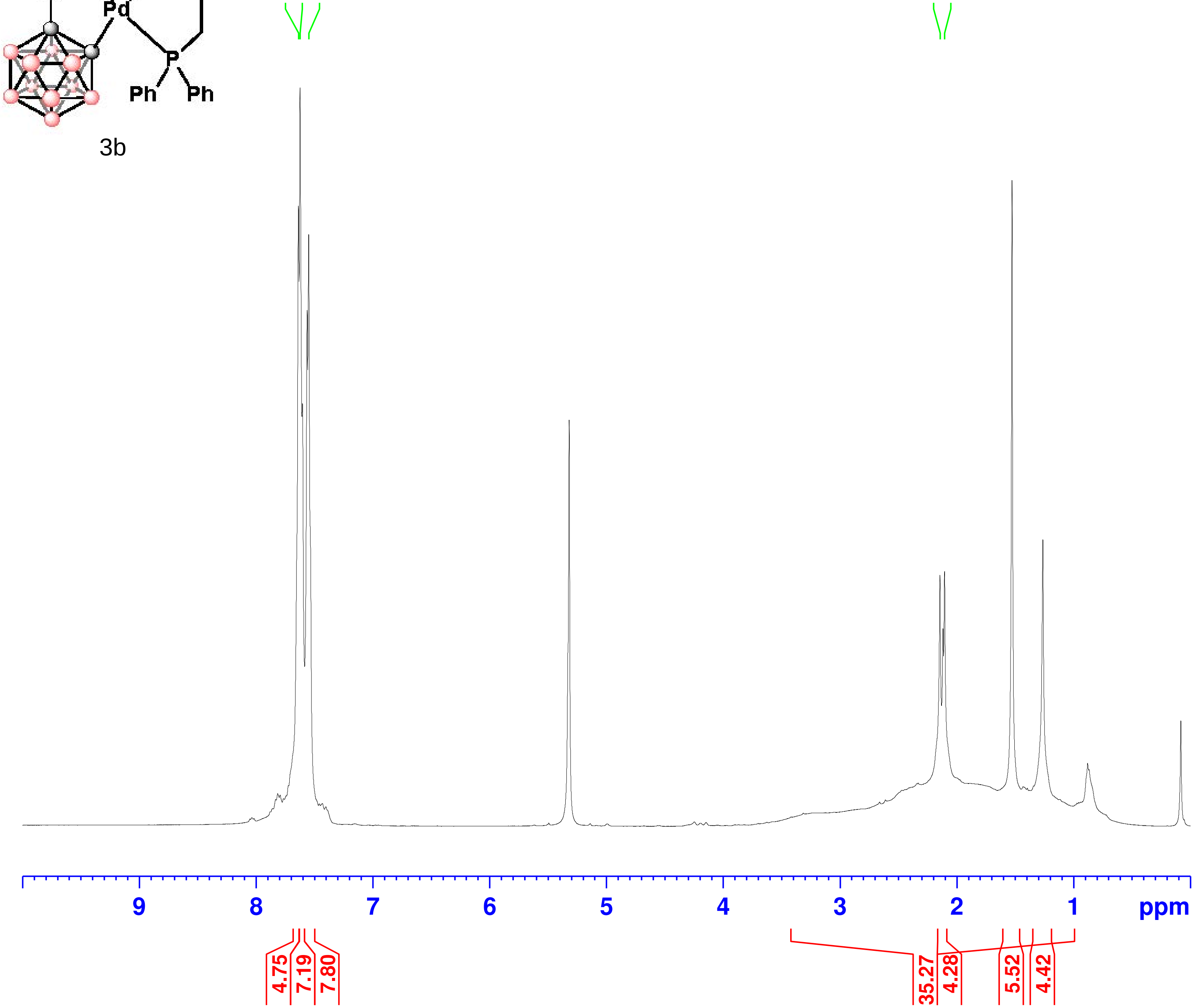


```
Current Data Parameters
NAME      kk-1-241 pd(bc)(dppe) pure
EXPNO                    20
PROCNO                    1

F2 - Acquisition Parameters
Date_              20150920
Time                  19.02
INSTRUM               av500
PROBHD    5 mm DCH 13C-1
PULPROG                zg30
TD                    65536
SOLVENT              CD2Cl2
NS                       64
DS                        0
SWH             10000.000 Hz
FIDRES           0.152588 Hz
AQ              3.2767999 sec
RG                    23.34
DW                  50.000 usec
DE                   10.00 usec
TE                   298.0 K
D1             2.00000000 sec
TD0                       1

======== CHANNEL f1 ========
SFO1         500.1330008 MHz
NUC1                     1H
P1                   10.00 usec
PLW1           13.50000000 W

F2 - Processing parameters
SI                    65536
SF           500.1300196 MHz
WDW                      EM
SSB       0
LB                     0.30 Hz
GB        0
PC                     1.00
```

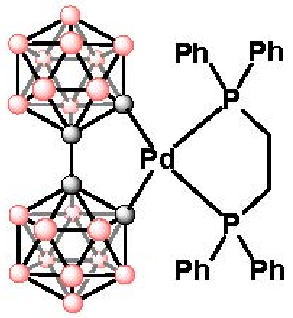

3b

11B NMR

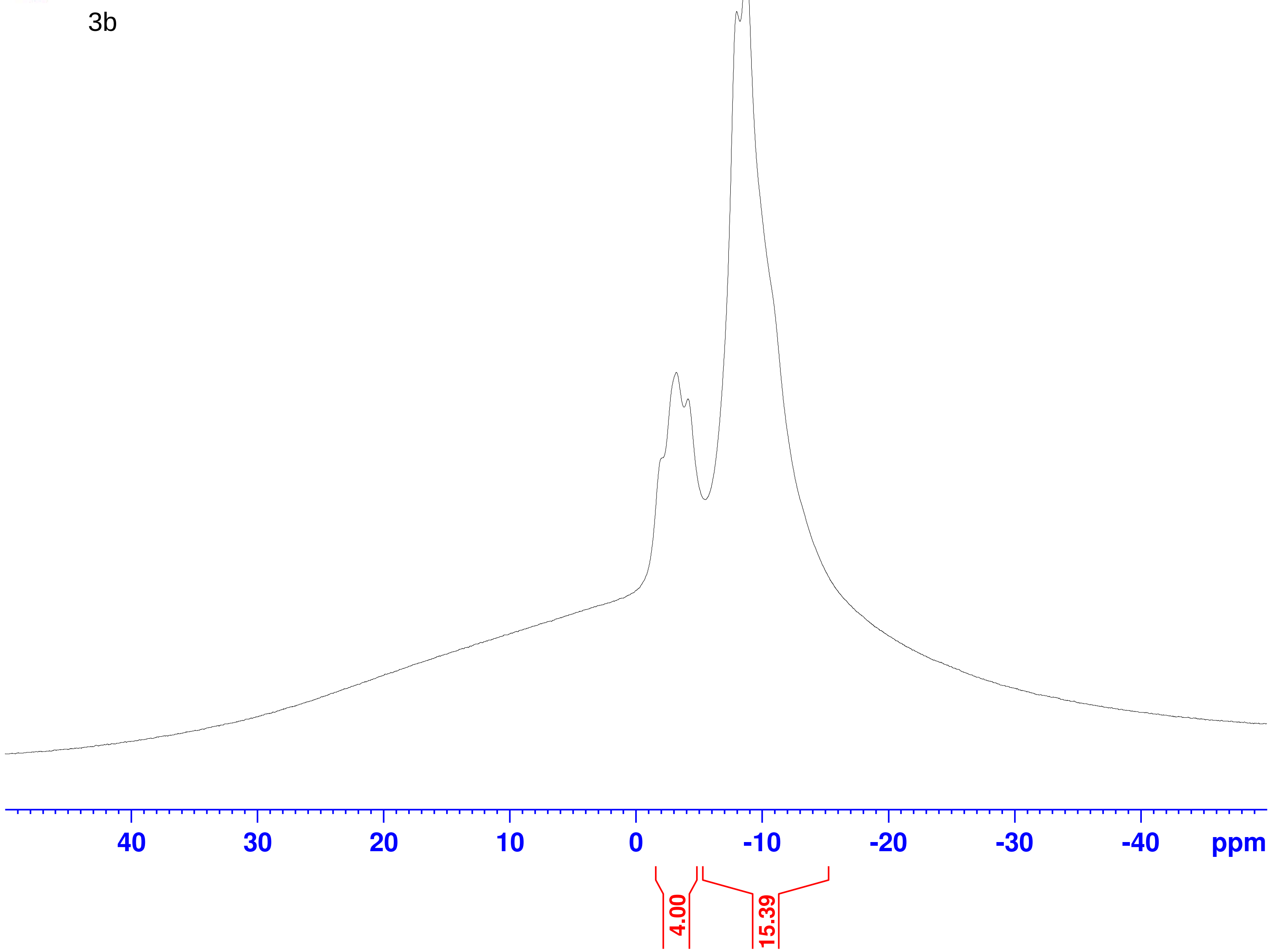

Current Data Parameters
NAME kk-1-241 pd(bc)(dppe) pure 11b
EXPNO 2
PROCNO 1

F2 - Acquisition Parameters
Date_ 20150922
Time 14.26
INSTRUM drx500
PROBHD 5 mm bb-Z Z800
PULPROG zgdc
TD 7168
SOLVENT CD2Cl2
NS 4096
DS 4
SWH 64102.562 Hz
FIDRES 8.942880 Hz
AQ 0.0559104 sec
RG 574.7
DW 7.800 usec
DE 6.00 usec
TE 296.9 K
D1 0.00000400 sec
d11 0.03000000 sec
TD0 1

======== CHANNEL f1 ========
NUC1 11B
P1 8.00 usec
PL1 -2.00 dB
SFO1 160.5257140 MHz

======== CHANNEL f2 ========
CPDPRG[2 waltz16
NUC2 off
PCPD2 100.00 usec
PL2 0 dB
PL12 17.52 dB
SFO2 500.3300000 MHz

F2 - Processing parameters
SI 32768
SF 160.5257717 MHz
WDW EM
SSB 0
LB 10.00 Hz
GB 0
PC 1.40

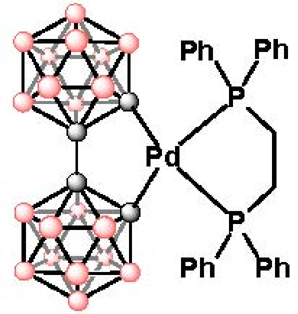

3b

11B{1H} NMR

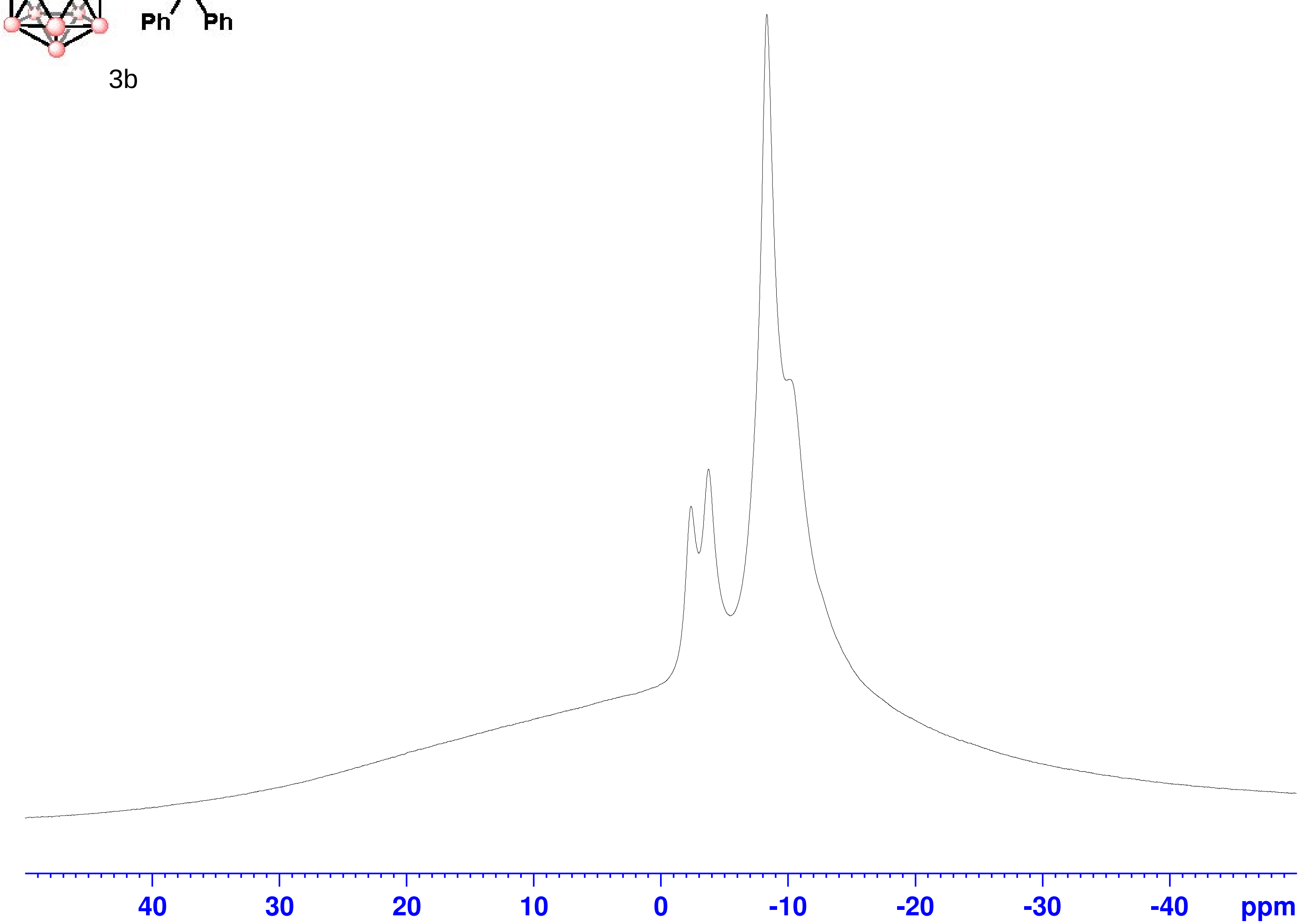


```
Current Data Parameters
NAME      kk-1-241 pd(bc)(dppe) pure 11b
EXPNO                     1
PROCNO                    1

F2 - Acquisition Parameters
Date_              20150922
Time                  13.54
INSTRUM              drx500
PROBHD    5 mm bb-Z Z800
PULPROG                zgdc
TD                     7168
SOLVENT             CD2Cl2
NS                     4096
DS                        4
SWH            64102.562 Hz
FIDRES          8.942880 Hz
AQ             0.0559104 sec
RG                    574.7
DW                7.800 usec
DE                 6.00 usec
TE                  297.1 K
D1            0.00000400 sec
d11           0.03000000 sec
TD0                       1

======== CHANNEL f1 ========
NUC1                    11B
P1                 8.00 usec
PL1                -2.00 dB
SFO1         160.5257136 MHz

======== CHANNEL f2 ========
CPDPRG[2            waltz16
NUC2                     1H
PCPD2            100.00 usec
PL2       0 dB
PL12               17.52 dB
SFO2         500.3330020 MHz

F2 - Processing parameters
SI                    32768
SF           160.5257717 MHz
WDW                      EM
SSB       0
LB                 10.00 Hz
GB        0
PC                     1.40
```

13C{1H} NMR

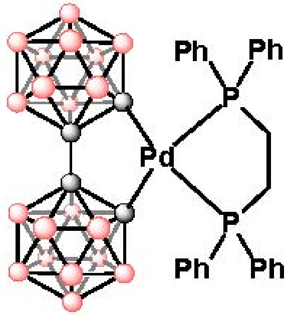

3b

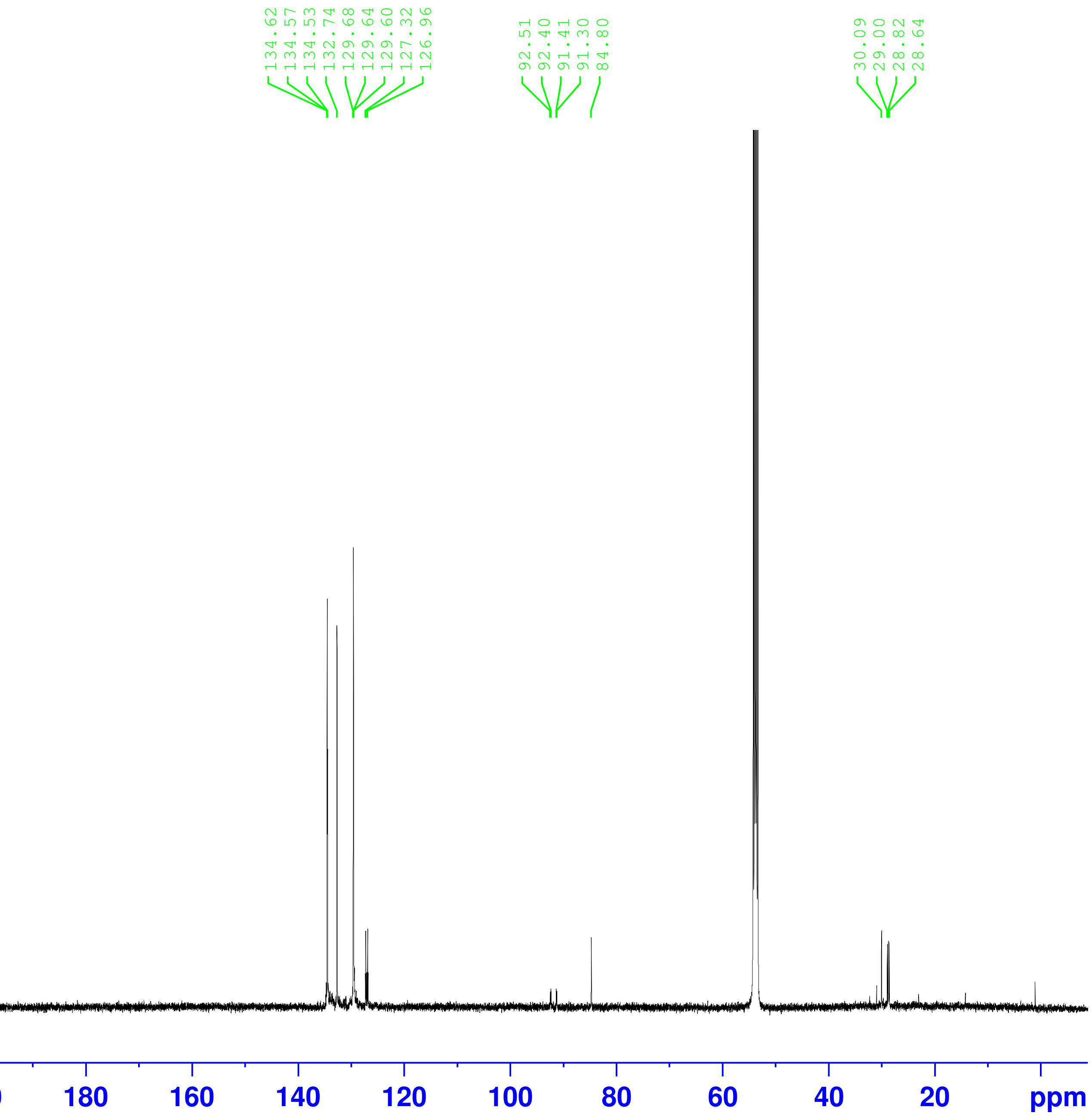

Current Data Parameters
NAME kk-1-241 pd(bc)(dppe) pure
EXPNO 22
PROCNO 1

F2 - Acquisition Parameters
Date_ 20150920
Time 19.56
INSTRUM av500
PROBHD 5 mm DCH 13C-1
PULPROG zgpg30
TD 65536
SOLVENT CD2Cl2
NS 1024
DS 2
SWH 31250.000 Hz
FIDRES 0.476837 Hz
AQ 1.0485760 sec
RG 204.54
DW 16.000 usec
DE 18.00 usec
TE 298.0 K
D1 2.00000000 sec
D11 0.03000000 sec
TD0 1

======== CHANNEL f1 ========
SFO1 125.7722511 MHz
NUC1 13C
P1 9.63 usec
PLW1 23.00000000 W

======== CHANNEL f2 ========
SFO2 500.1330008 MHz
NUC2 1H
CPDPRG[2 waltz16
PCPD2 80.00 usec
PLW2 13.50000000 W
PLW12 0.21094000 W
PLW13 0.13500001 W

F2 - Processing parameters
SI 131072
SF 125.7577377 MHz
WDW EM
SSB 0
LB 1.00 Hz
GB 0
PC 1.40

31P{1H} NMR

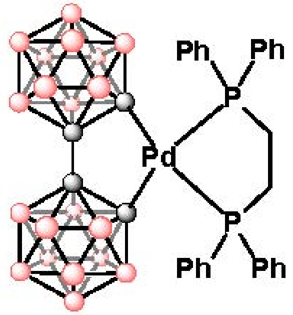

3b

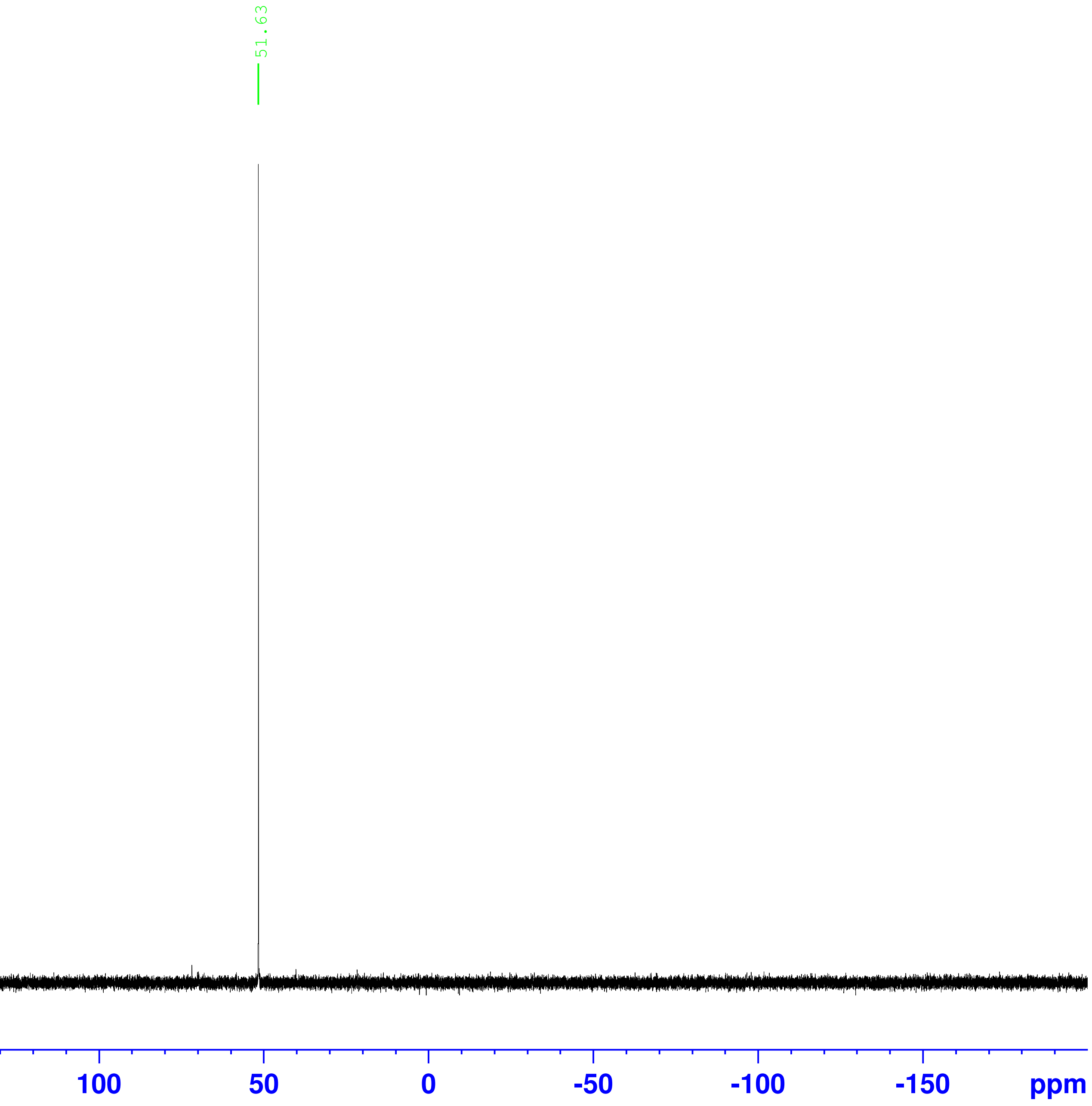


```
Current Data Parameters
NAME      kk-1-241 pd(bc)(dppe) pure, 31p
EXPNO                  1
PROCNO                 1

F2 - Acquisition Parameters
Date_           20150920
Time               15.47
INSTRUM           drx500
PROBHD   5 mm bb-Z Z800
PULPROG          zgdc30
TD                131072
SOLVENT          CD2Cl2
NS                   128
DS                     0
SWH          100000.000 Hz
FIDRES         0.762939 Hz
AQ            0.6553600 sec
RG                9195.2
DW                 5.000 usec
DE                  6.00 usec
TE                 297.3 K
D1           2.00000000 sec
d11          0.03000000 sec
TD0                    1

======== CHANNEL f1 ========
NUC1                 31P
P1                  9.00 usec
PL1      0 dB
SFO1         202.5370460 MHz

======== CHANNEL f2 ========
CPDPRG[2         waltz16
NUC2                  1H
PCPD2             100.00 usec
PL2      0 dB
PL12               17.52 dB
SFO2         500.3320013 MHz

F2 - Processing parameters
SI                131072
SF           202.5371980 MHz
WDW                   EM
SSB      0
LB                  1.00 Hz
GB       0
PC                  1.40
```

1H NMR

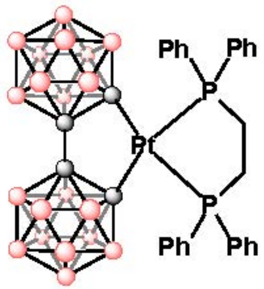

3c

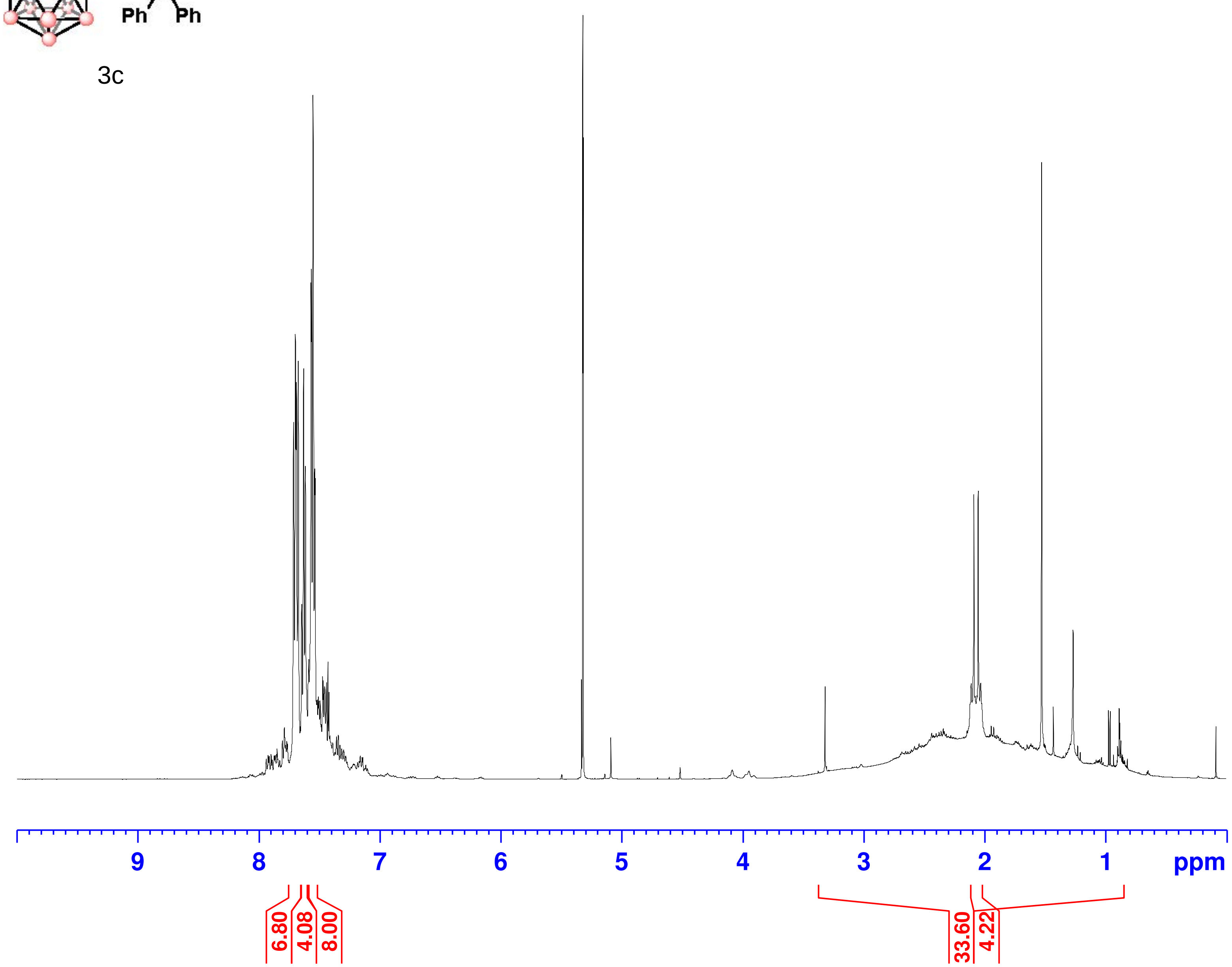


```
Current Data Parameters
NAME      KK-1-285 Pt(bc)(dppe)
EXPNO                   10
PROCNO                   1

F2 - Acquisition Parameters
Date_             20151221
Time                 18.41
INSTRUM              av500
PROBHD    5 mm DCH 13C-1
PULPROG               zg30
TD                   65536
SOLVENT             CD2Cl2
NS                      64
DS                       0
SWH              10000.000 Hz
FIDRES            0.152588 Hz
AQ               3.2767999 sec
RG                   12.14
DW                  50.000 usec
DE                   10.00 usec
TE                   298.0 K
D1              2.00000000 sec
TD0                      1

======== CHANNEL f1 ========
SFO1           500.1330008 MHz
NUC1                    1H
P1                   10.00 usec
PLW1           13.50000000 W

F2 - Processing parameters
SI                   65536
SF             500.1300194 MHz
WDW                     EM
SSB       0
LB                    0.30 Hz
GB        0
PC                    1.00
```

11B NMR

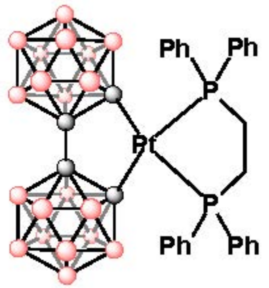

3c

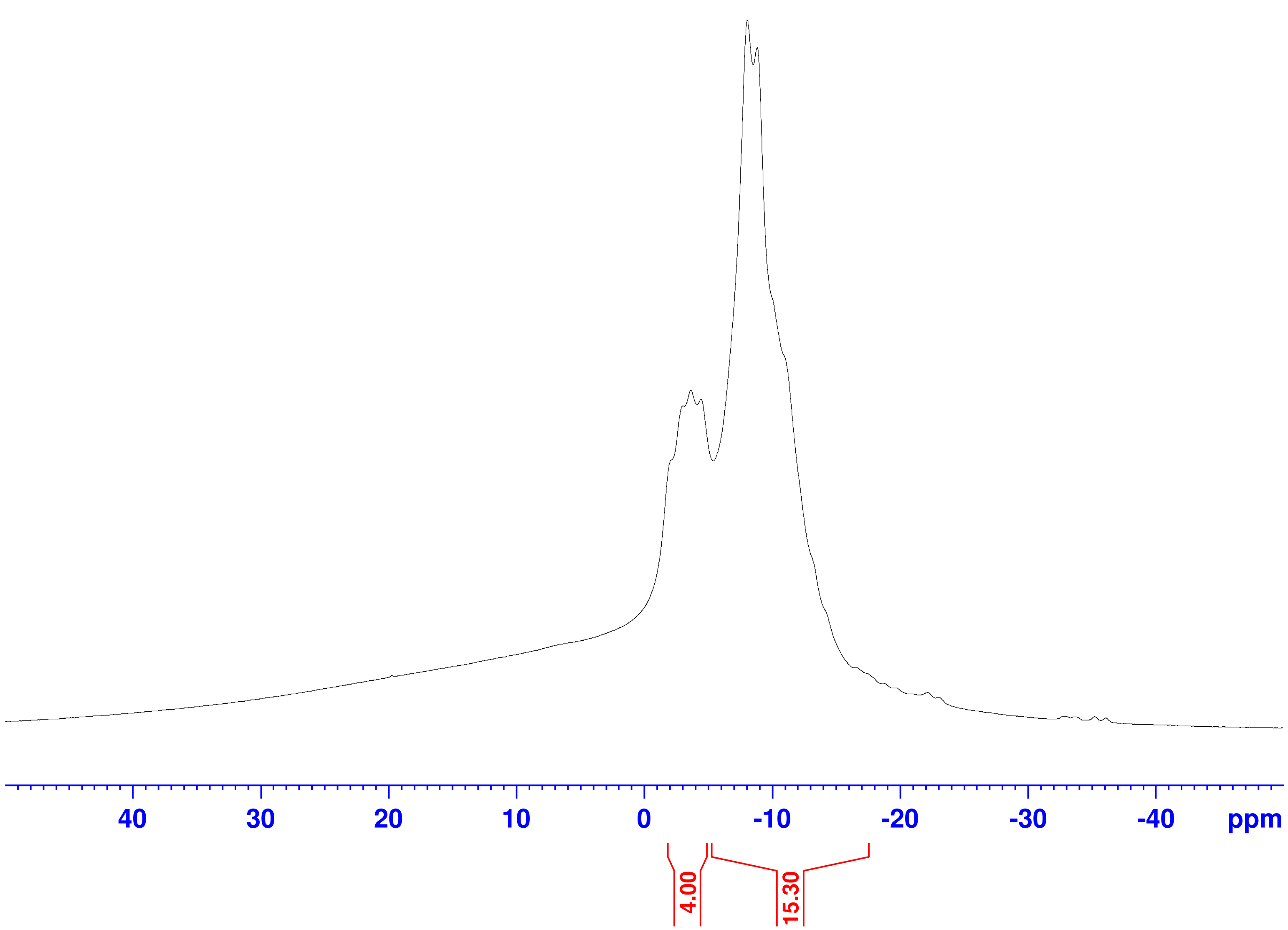


```
Current Data Parameters
NAME       KK-1-285 Pt(bc)(dppe) pure 11B
EXPNO                   3
PROCNO                  1

F2 - Acquisition Parameters
Date_            20151221
Time                18.29
INSTRUM            drx500
PROBHD    5 mm bb-Z Z800
PULPROG              zgdc
TD                   7168
SOLVENT            CD2Cl2
NS                   4096
DS                      4
SWH           64102.562 Hz
FIDRES         8.942880 Hz
AQ            0.0559104 sec
RG                  406.4
DW                  7.800 usec
DE                   6.00 usec
TE                  296.7 K
D1           0.00000400 sec
d11          0.03000000 sec
TD0                     1

======== CHANNEL f1 ========
NUC1                  11B
P1                   8.00 usec
PL1                 -2.00 dB
SFO1         160.5257140 MHz

======== CHANNEL f2 ========
CPDPRG[2          waltz16
NUC2                  off
PCPD2              100.00 usec
PL2         0 dB
PL12                17.52 dB
SFO2         500.3300000 MHz

F2 - Processing parameters
SI                  32768
SF           160.5257957 MHz
WDW                    EM
SSB         0
LB                  10.00 Hz
GB          0
PC                   1.40
```

11B{1H} NMR

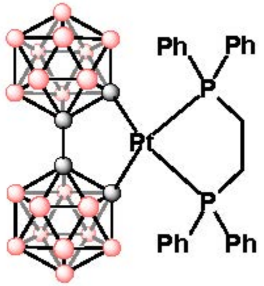

3c

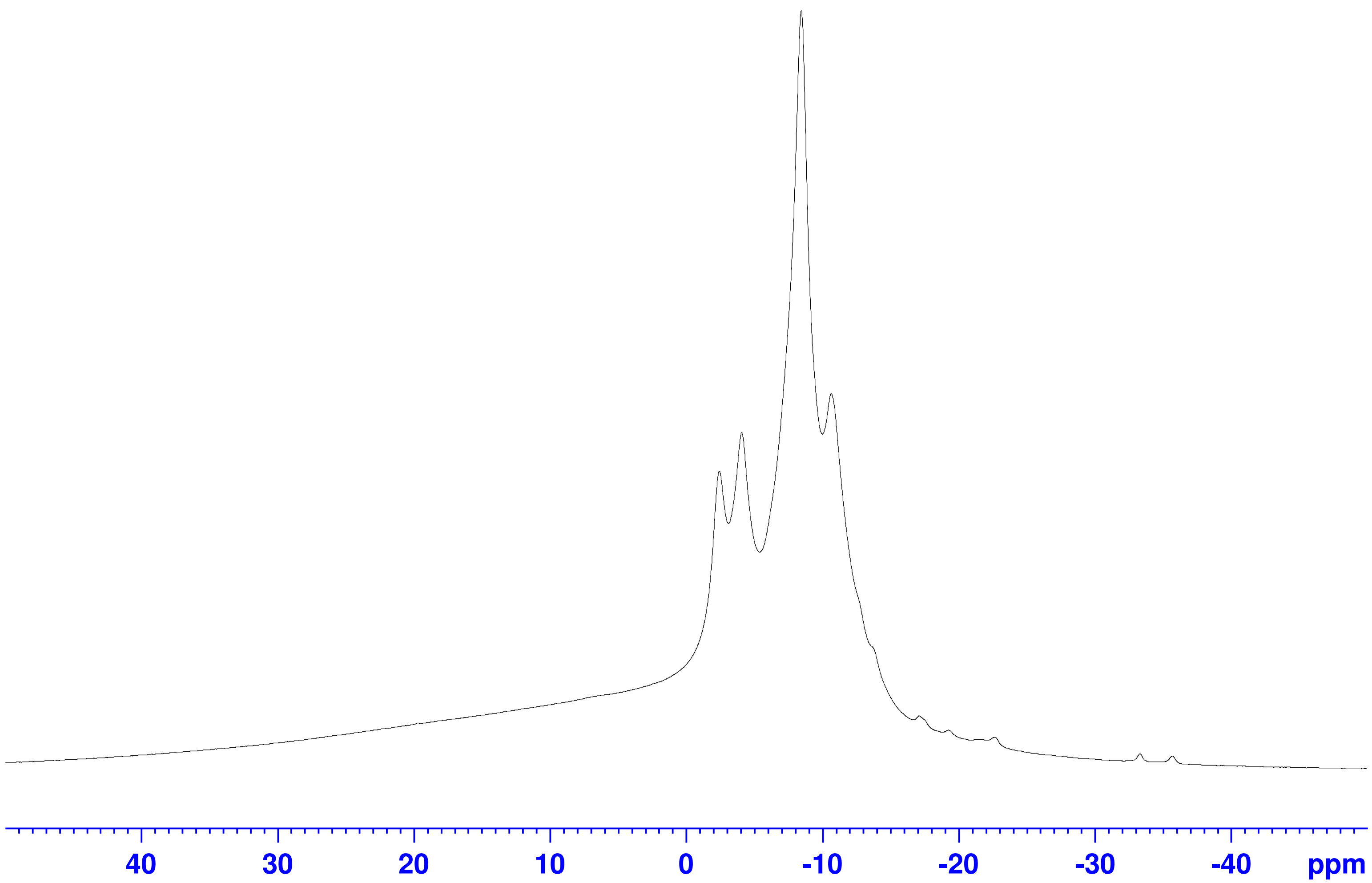

Current Data Parameters

NAME	KK-1-285 Pt(bc)(dppe) pure 11B	
EXPNO	2	
PROCNO	1	

F2 - Acquisition Parameters

Date_	20151221	
Time	18.21	
INSTRUM	drx500	
PROBHD	5 mm bb-Z Z800	
PULPROG	zgdc	
TD	7168	
SOLVENT	CD2Cl2	
NS	4096	
DS	4	
SWH	64102.562	Hz
FIDRES	8.942880	Hz
AQ	0.0559104	sec
RG	406.4	
DW	7.800	usec
DE	6.00	usec
TE	297.0	K
D1	0.00000400	sec
d11	0.03000000	sec
TD0	1	

======== CHANNEL f1 ========

NUC1	11B	
P1	8.00	usec
PL1	-2.00	dB
SFO1	160.5257136	MHz

======== CHANNEL f2 ========

CPDPRG[2	waltz16	
NUC2	1H	
PCPD2	100.00	usec
PL2	0	dB
PL12	17.52	dB
SFO2	500.3330020	MHz

F2 - Processing parameters

SI	32768	
SF	160.5257957	MHz
WDW	EM	
SSB	0	
LB	10.00	Hz
GB	0	
PC	1.40	

13C{1H} NMR

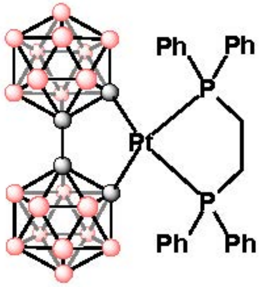

3c

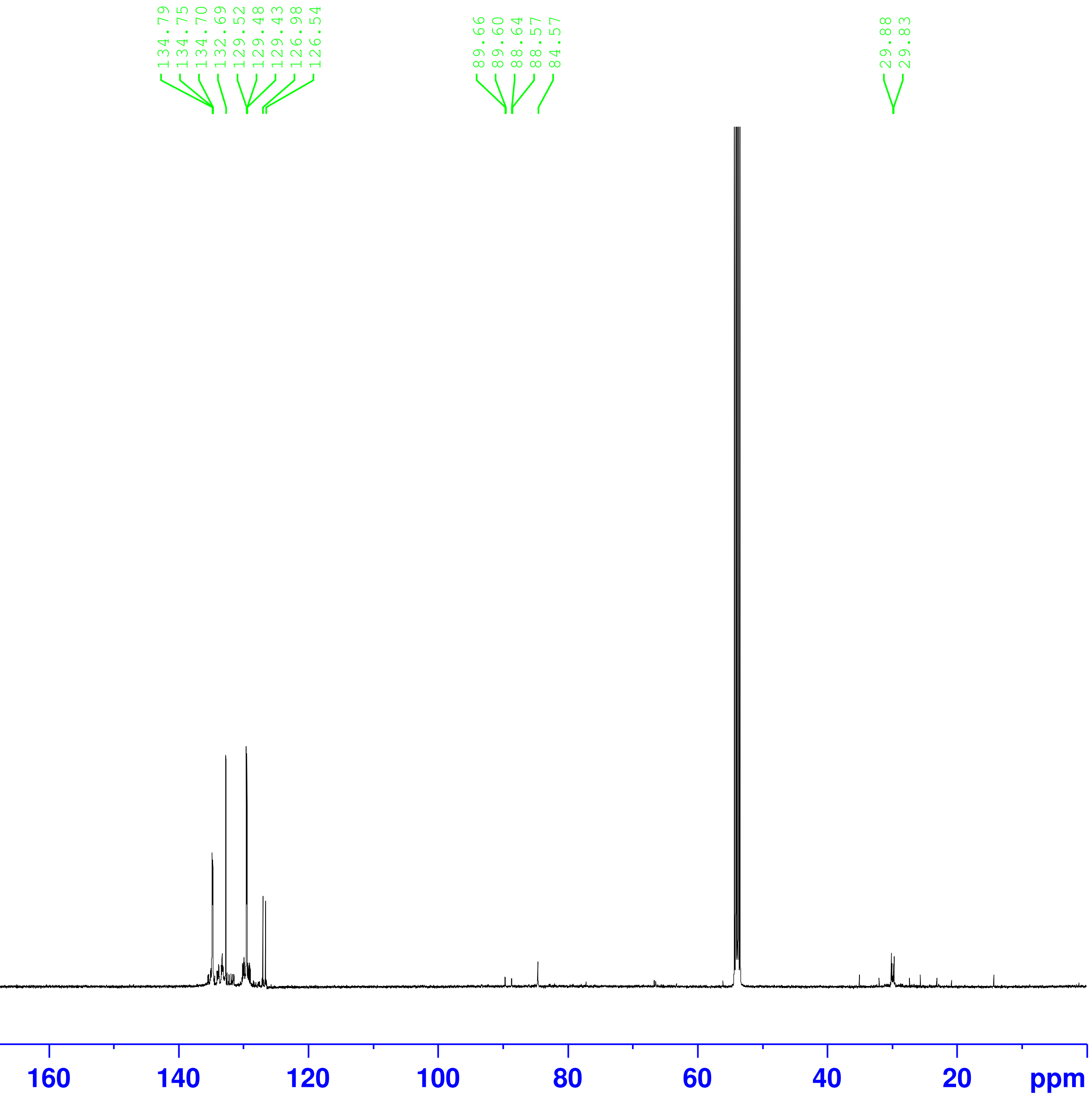


```
Current Data Parameters
NAME      KK-1-285 Pt(bc)(dppe)
EXPNO                    11
PROCNO                    1

F2 - Acquisition Parameters
Date_              20151221
Time                  19.37
INSTRUM               av500
PROBHD    5 mm DCH 13C-1
PULPROG             zgpg30
TD                    65536
SOLVENT             CD2Cl2
NS                     1024
DS                        2
SWH            31250.000 Hz
FIDRES          0.476837 Hz
AQ             1.0485760 sec
RG                   204.54
DW                16.000 usec
DE                 18.00 usec
TE                 298.0 K
D1            2.00000000 sec
D11           0.03000000 sec
TD0                       1

======== CHANNEL f1 ========
SFO1         125.7722511 MHz
NUC1                    13C
P1                  9.63 usec
PLW1         23.00000000 W

======== CHANNEL f2 ========
SFO2         500.1330008 MHz
NUC2                     1H
CPDPRG[2            waltz16
PCPD2              80.00 usec
PLW2         13.50000000 W
PLW12         0.21094000 W
PLW13         0.13500001 W

F2 - Processing parameters
SI                   131072
SF           125.7577387 MHz
WDW                      EM
SSB      0
LB                  1.00 Hz
GB       0
PC                     1.40
```

31P{1H} NMR

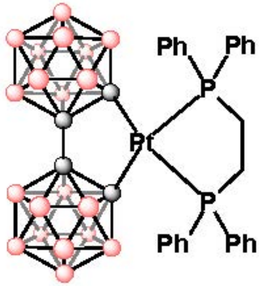

3c

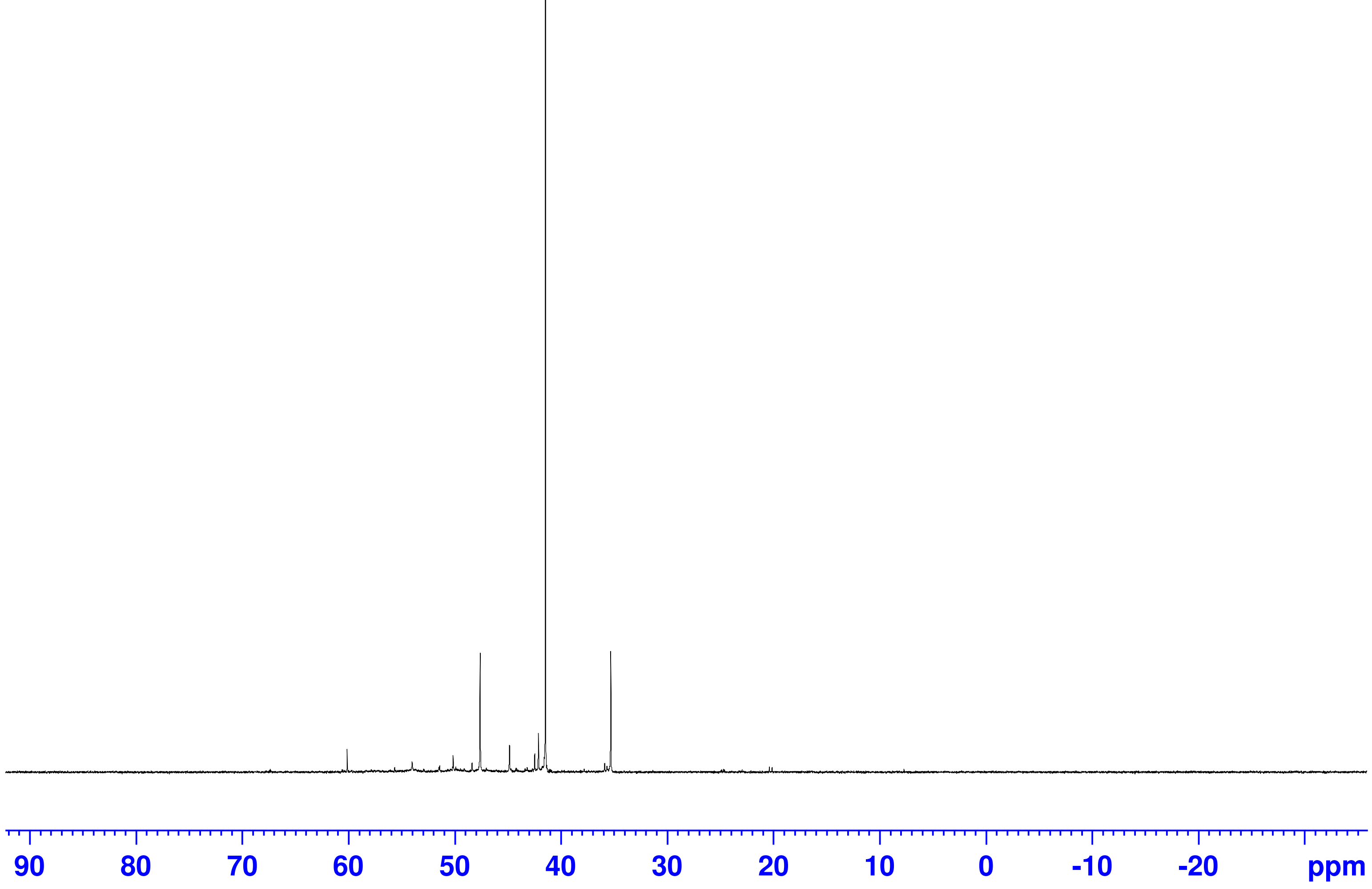


```
Current Data Parameters
NAME      KK-1-285 Pt(bc)(dppe) pure 31P
EXPNO                    2
PROCNO                   1

F2 - Acquisition Parameters
Date_             20151221
Time                 17.09
INSTRUM             drx500
PROBHD    5 mm bb-Z Z800
PULPROG            zgdc30
TD                  131072
SOLVENT             CD2Cl2
NS                    1024
DS                       0
SWH            100000.000 Hz
FIDRES           0.762939 Hz
AQ              0.6553600 sec
RG                   13004
DW                   5.000 usec
DE                    6.00 usec
TE                   297.1 K
D1              2.00000000 sec
d11             0.03000000 sec
TD0                      1

======== CHANNEL f1 ========
NUC1                   31P
P1                    9.00 usec
PL1       0 dB
SFO1           202.5370460 MHz

======== CHANNEL f2 ========
CPDPRG[2           waltz16
NUC2                    1H
PCPD2               100.00 usec
PL2       0 dB
PL12                 17.52 dB
SFO2           500.3320013 MHz

F2 - Processing parameters
SI                  131072
SF             202.5371730 MHz
WDW                     EM
SSB       0
LB                    1.00 Hz
GB        0
PC                    1.40
```

1H NMR

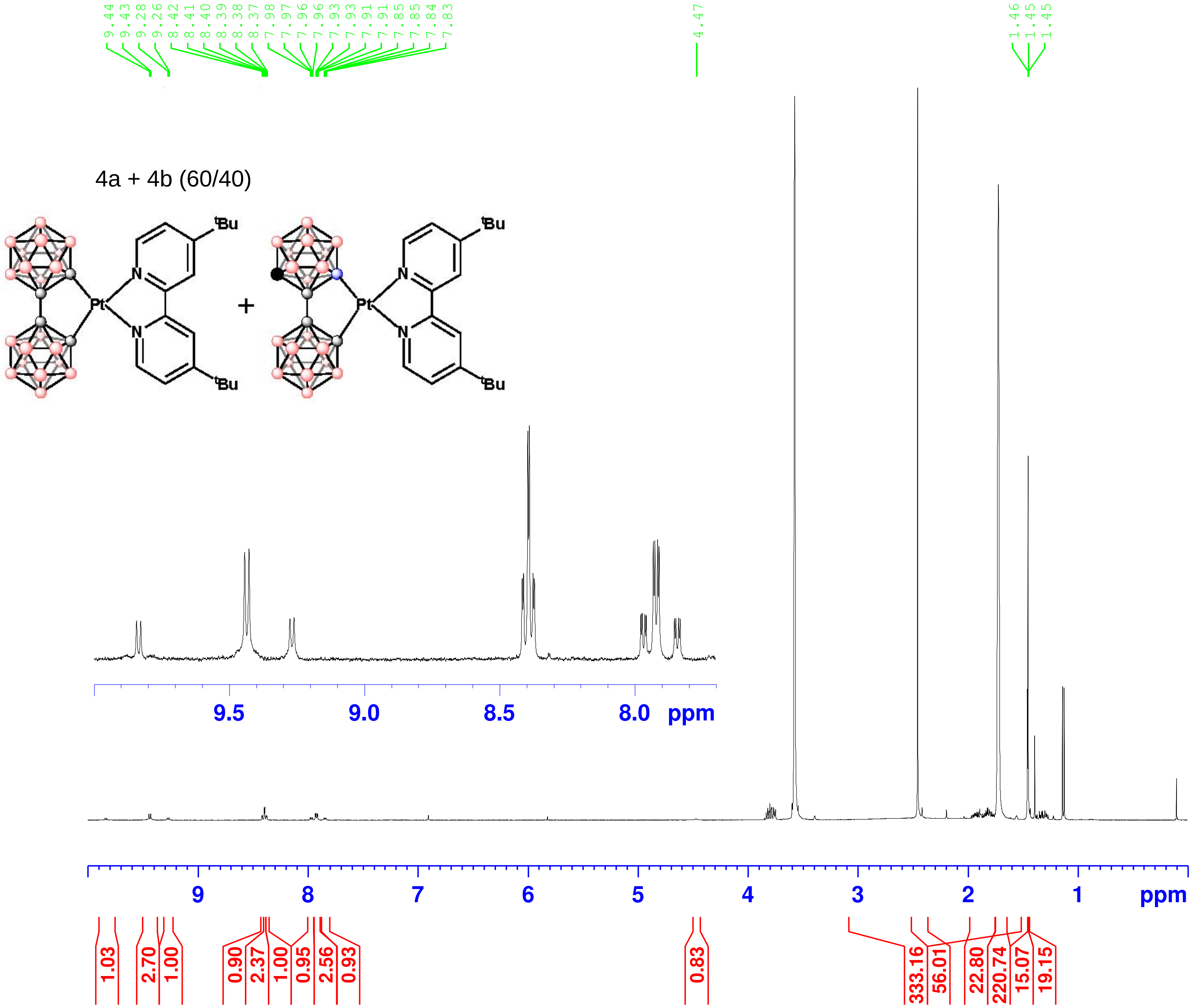

Current Data Parameters
NAME Sep25-2015-spokoyny
EXPNO 40
PROCNO 1

F2 - Acquisition Parameters
Date_ 20150925
Time 14.39
INSTRUM av400
PROBHD 5 mm PABBO BB/
PULPROG zg30
TD 52882
SOLVENT THF
NS 64
DS 0
SWH 8012.820 Hz
FIDRES 0.151523 Hz
AQ 3.2998369 sec
RG 155.85
DW 62.400 usec
DE 6.50 usec
TE 299.0 K
D1 2.00000000 sec
TD0 1

======== CHANNEL f1 ========
SFO1 400.1324008 MHz
NUC1 1H
P1 15.00 usec
PLW1 13.00000000 W

F2 - Processing parameters
SI 65536
SF 400.1300176 MHz
WDW EM
SSB 0
LB 0.30 Hz
GB 0
PC 1.00

1H NMR

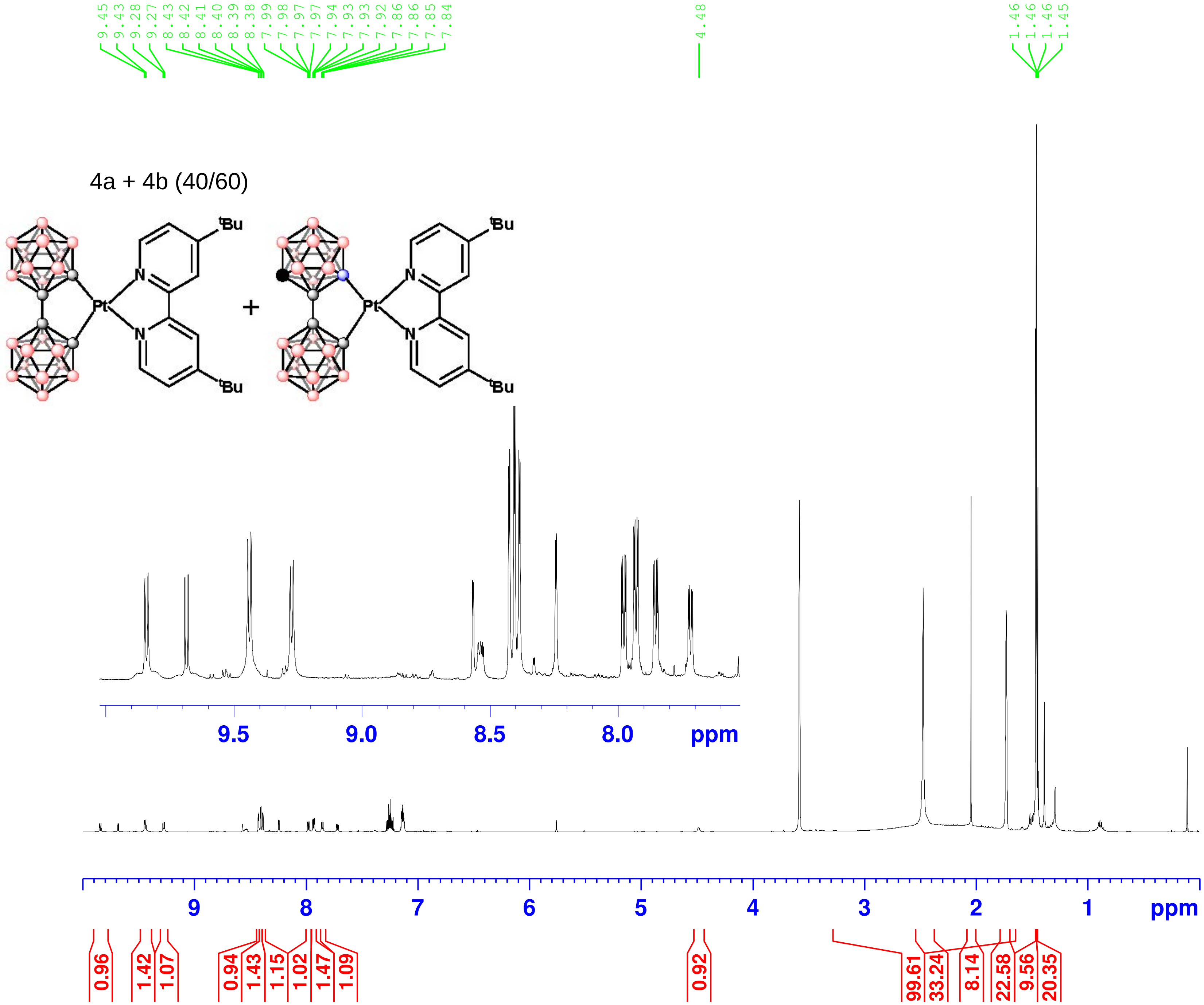


```
Current Data Parameters
NAME      KK-1-297 Pt(bc)(dtb-bpy)
EXPNO                 10
PROCNO                 1

F2 - Acquisition Parameters
Date_           20160127
Time                1.10
INSTRUM            av500
PROBHD    5 mm DCH 13C-1
PULPROG             zg30
TD                 65536
SOLVENT              THF
NS                   128
DS                     0
SWH            10000.000 Hz
FIDRES          0.152588 Hz
AQ             3.2767999 sec
RG                 23.34
DW                50.000 usec
DE                 10.00 usec
TE                 298.0 K
D1            2.00000000 sec
TD0                    1

======== CHANNEL f1 ========
SFO1         500.1330008 MHz
NUC1                  1H
P1                 10.00 usec
PLW1         13.50000000 W

F2 - Processing parameters
SI                 65536
SF           500.1290892 MHz
WDW                   EM
SSB      0
LB                  0.30 Hz
GB       0
PC                  1.00
```

11B NMR

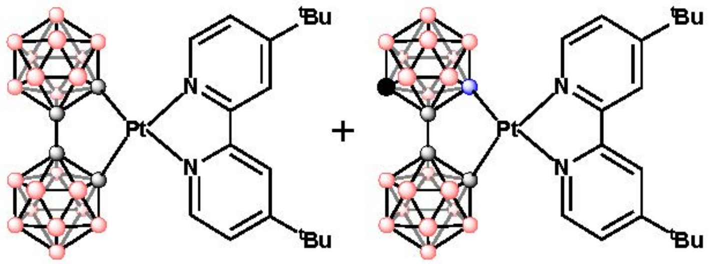

4a + 4b (40/60)

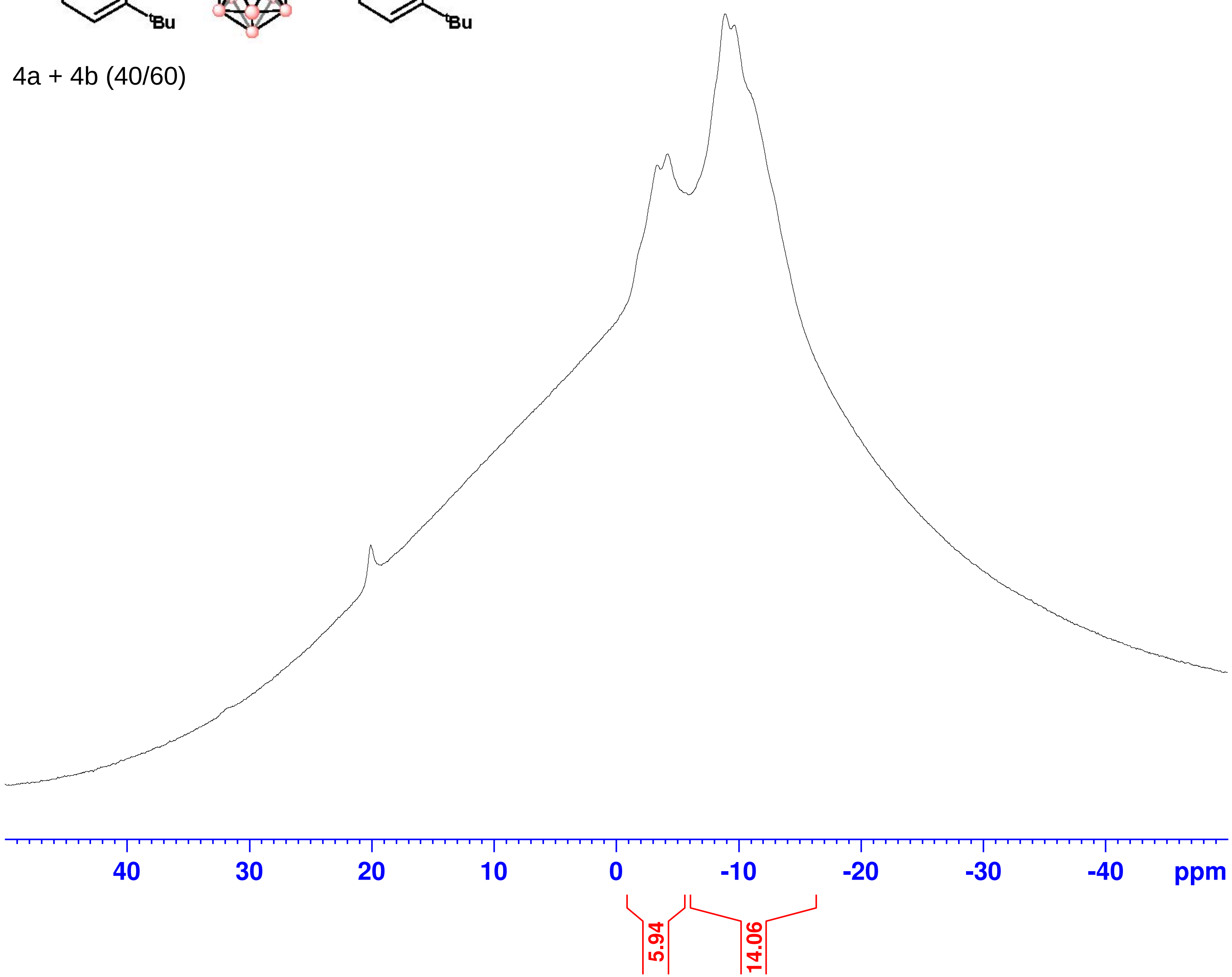


```
Current Data Parameters
NAME      KK-1-297 Pt(bc)(dtb-bpy) 11B
EXPNO                       2
PROCNO                      1

F2 - Acquisition Parameters
Date_                20160129
Time                    12.58
INSTRUM                drx500
PROBHD    5 mm bb-Z Z800
PULPROG                  zgdc
TD                       7168
SOLVENT                   THF
NS                       8192
DS                          4
SWH                 64102.562 Hz
FIDRES               8.942880 Hz
AQ                  0.0559104 sec
RG                      574.7
DW                      7.800 usec
DE                       6.00 usec
TE                      296.7 K
D1                 0.00000400 sec
d11                0.03000000 sec
TD0                         1

======== CHANNEL f1 ========
NUC1                      11B
P1                       8.00 usec
PL1                     -2.00 dB
SFO1              160.5257140 MHz

======== CHANNEL f2 ========
CPDPRG[2              waltz16
NUC2                      off
PCPD2                  100.00 usec
PL2       0 dB
PL12                    17.52 dB
SFO2              500.3300000 MHz

F2 - Processing parameters
SI                      32768
SF                160.5257546 MHz
WDW                        EM
SSB       0
LB                      10.00 Hz
GB        0
PC                       1.40
```

11B{1H} NMR

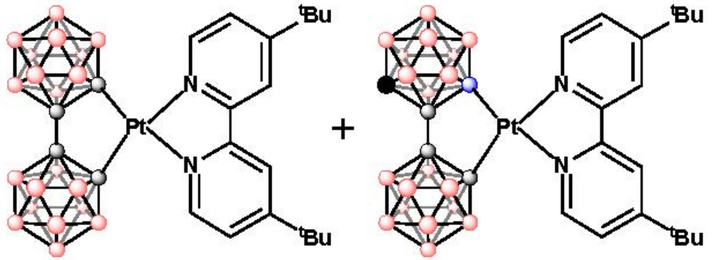

4a + 4b (40/60)

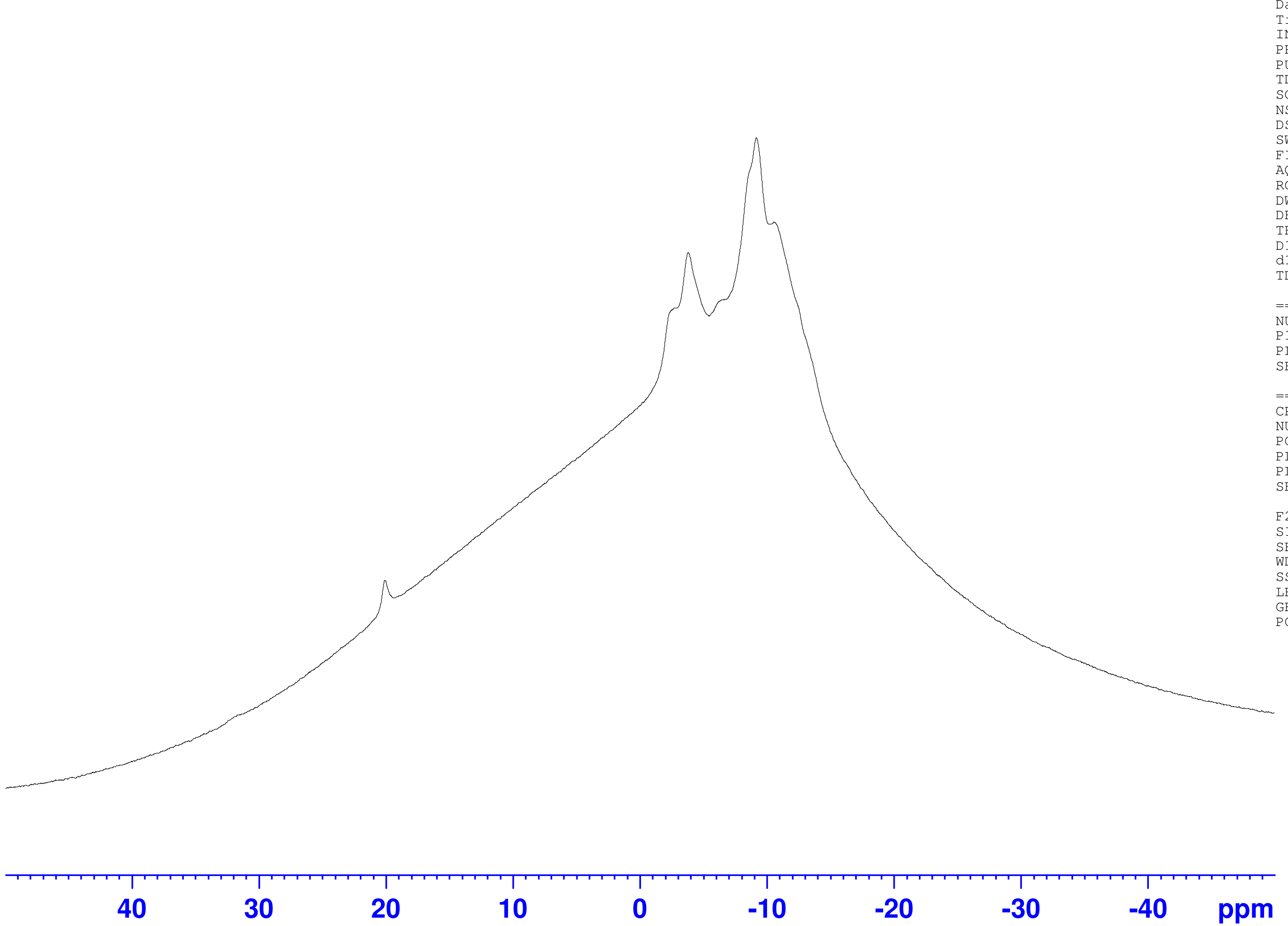


```
Current Data Parameters
NAME      KK-1-297 Pt(bc)(dtb-bpy) 11B
EXPNO                     1
PROCNO                    1

F2 - Acquisition Parameters
Date_              20160129
Time                  12.41
INSTRUM              drx500
PROBHD     5 mm bb-Z Z800
PULPROG                zgdc
TD                     7168
SOLVENT                 THF
NS                     8192
DS                        4
SWH               64102.562 Hz
FIDRES             8.942880 Hz
AQ                0.0559104 sec
RG                    574.7
DW                    7.800 usec
DE                     6.00 usec
TE                    297.1 K
D1               0.00000400 sec
d11              0.03000000 sec
TD0                       1

======== CHANNEL f1 ========
NUC1                    11B
P1                     8.00 usec
PL1                   -2.00 dB
SFO1            160.5257140 MHz

======== CHANNEL f2 ========
CPDPRG[2            waltz16
NUC2                     1H
PCPD2                100.00 usec
PL2          0 dB
PL12                  17.52 dB
SFO2            500.3330020 MHz

F2 - Processing parameters
SI                    32768
SF              160.5257546 MHz
WDW                      EM
SSB          0
LB                    10.00 Hz
GB           0
PC                     1.40
```

13C{1H} NMR

4a + 4b (40/60)

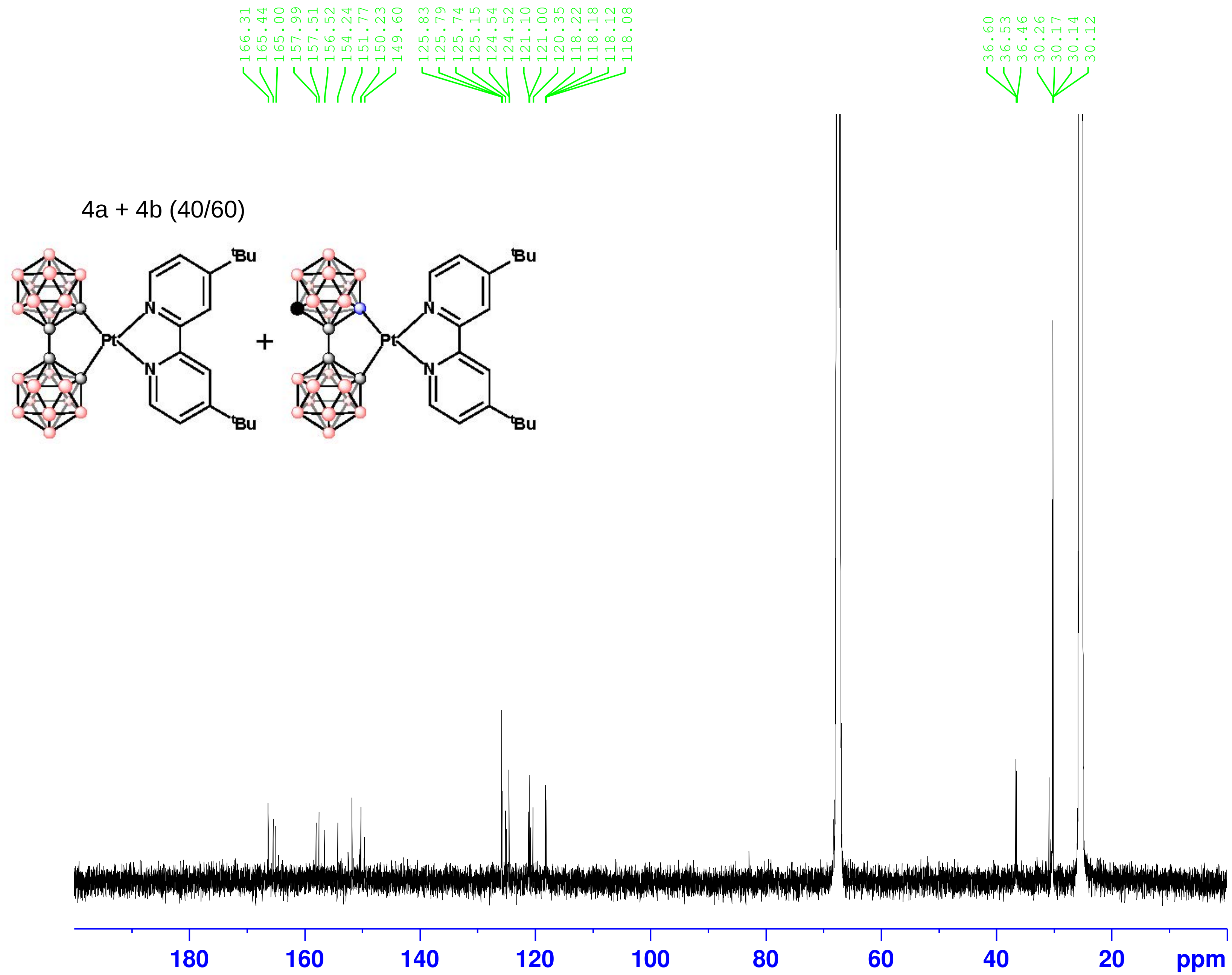

Current Data Parameters
NAME KK-1-297 Pt(bc)(dtb-bpy)
EXPNO 11
PROCNO 1

F2 - Acquisition Parameters
Date_ 20160127
Time 2.06
INSTRUM av500
PROBHD 5 mm DCH 13C-1
PULPROG zgpg30
TD 65536
SOLVENT THF
NS 1024
DS 2
SWH 31250.000 Hz
FIDRES 0.476837 Hz
AQ 1.0485760 sec
RG 204.54
DW 16.000 usec
DE 18.00 usec
TE 298.0 K
D1 2.00000000 sec
D11 0.03000000 sec
TD0 1

======== CHANNEL f1 ========
SFO1 125.7722511 MHz
NUC1 13C
P1 9.63 usec
PLW1 23.00000000 W

======== CHANNEL f2 ========
SFO2 500.1330008 MHz
NUC2 1H
CPDPRG[2 waltz16
PCPD2 80.00 usec
PLW2 13.50000000 W
PLW12 0.21094000 W
PLW13 0.13500001 W

F2 - Processing parameters
SI 131072
SF 125.7574332 MHz
WDW EM
SSB 0
LB 1.00 Hz
GB 0
PC 1.40

1H NMR

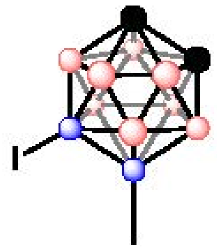

5

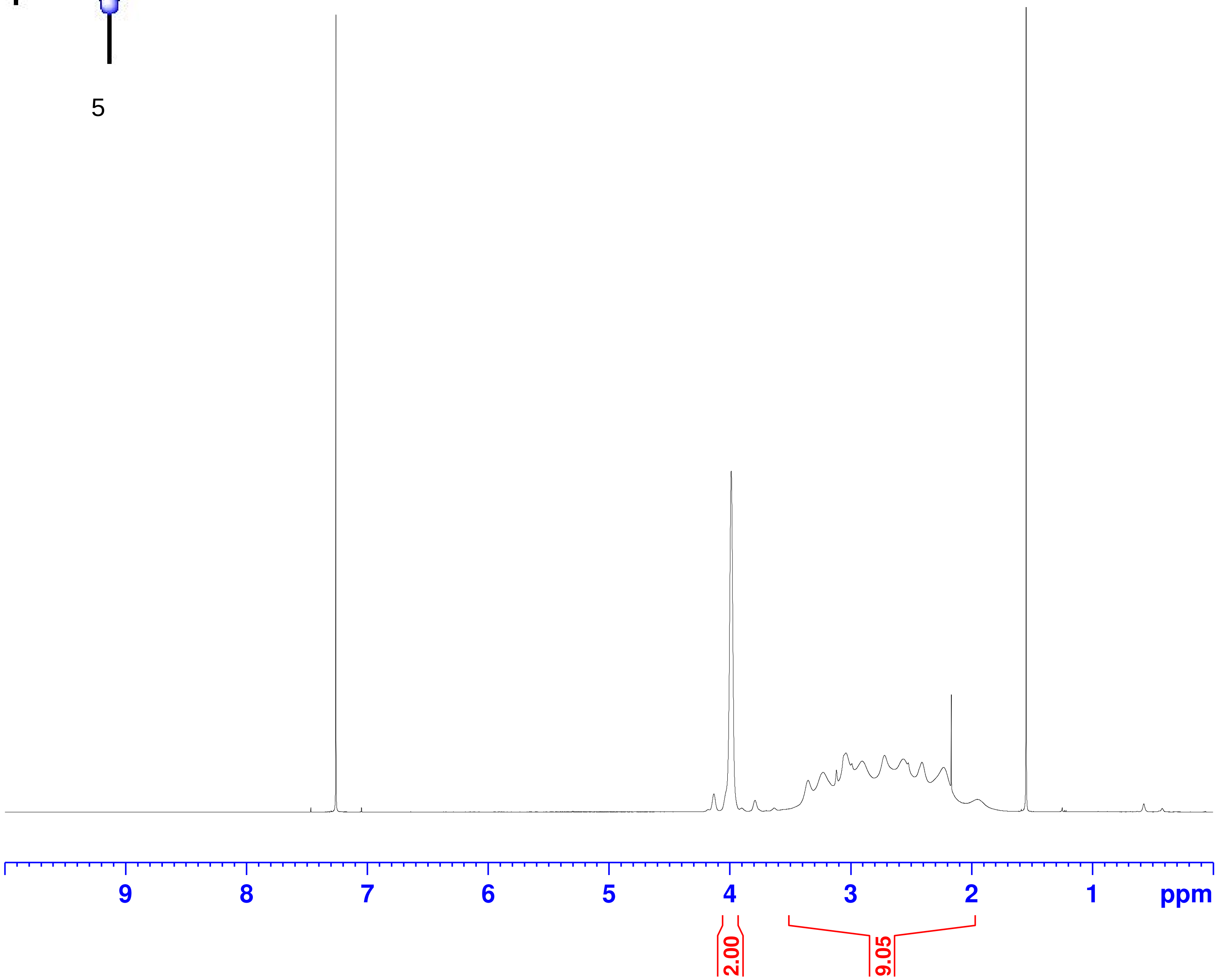

Current Data Parameters
NAME i2-ocb kk-1-265ish
EXPNO 10
PROCNO 1

F2 - Acquisition Parameters
Date_ 20151002
Time 11.30
INSTRUM av500
PROBHD 5 mm DCH 13C-1
PULPROG zg30
TD 65536
SOLVENT CDCl3
NS 32
DS 0
SWH 10000.000 Hz
FIDRES 0.152588 Hz
AQ 3.2767999 sec
RG 12.14
DW 50.000 usec
DE 10.00 usec
TE 298.0 K
D1 2.00000000 sec
TD0 1

======== CHANNEL f1 ========
SFO1 500.1330008 MHz
NUC1 1H
P1 10.00 usec
PLW1 13.50000000 W

F2 - Processing parameters
SI 65536
SF 500.1300122 MHz
WDW EM
SSB 0
LB 0.30 Hz
GB 0
PC 1.00

11B NMR

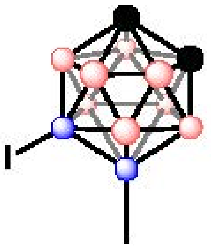

5

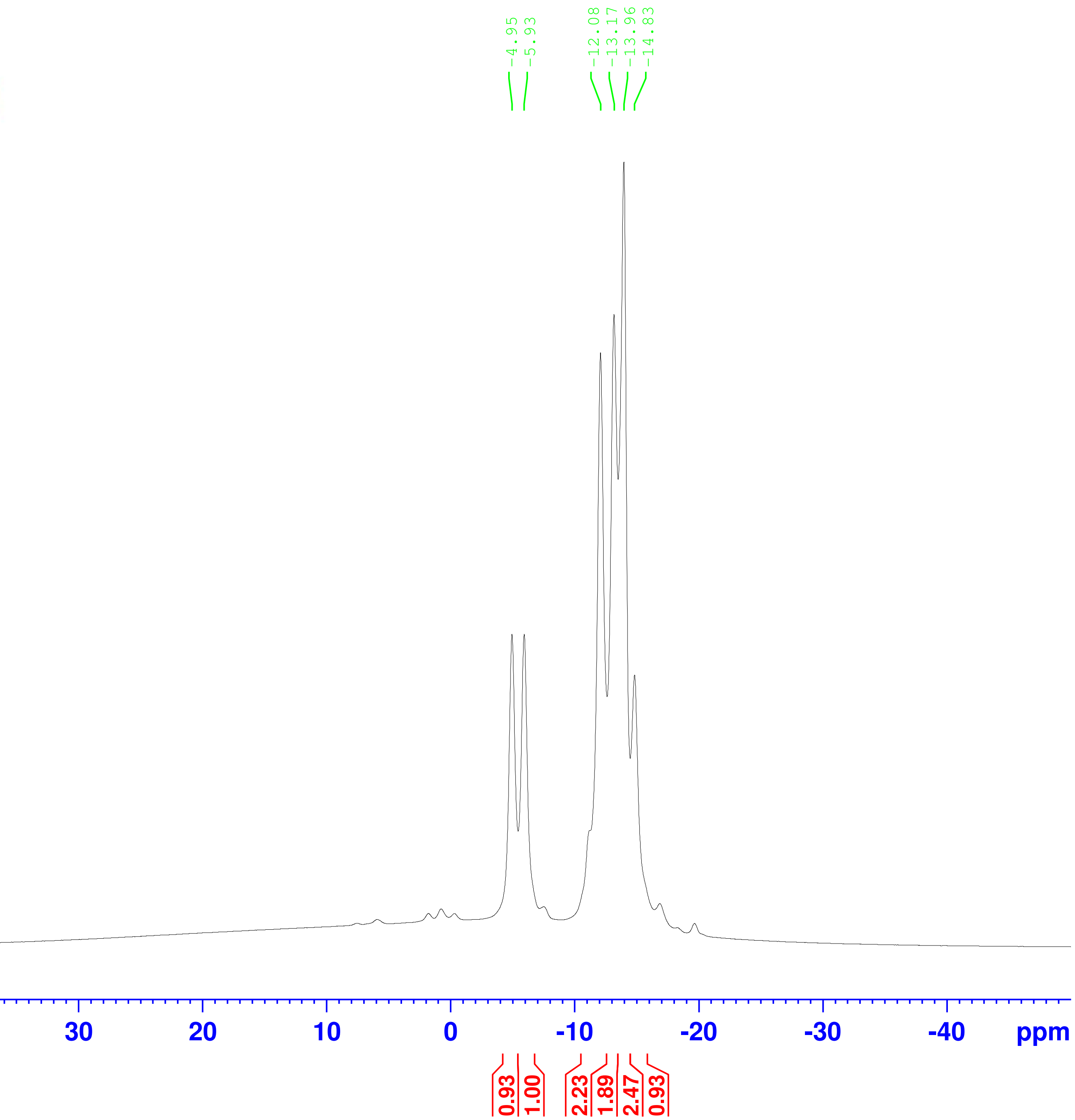

Current Data Parameters
NAME kk-1-264 i2-ocb
EXPNO 2
PROCNO 1

F2 - Acquisition Parameters

Date_	20151002
Time	12.33
INSTRUM	drx500
PROBHD	5 mm bb-Z Z800
PULPROG	zgdc
TD	7168
SOLVENT	CDCl3
NS	2048
DS	4
SWH	64102.562 Hz
FIDRES	8.942880 Hz
AQ	0.0559104 sec
RG	362
DW	7.800 usec
DE	6.00 usec
TE	296.9 K
D1	0.00000400 sec
d11	0.03000000 sec
TD0	1

======== CHANNEL f1 ========

NUC1	11B
P1	8.00 usec
PL1	-2.00 dB
SFO1	160.5257140 MHz

======== CHANNEL f2 ========

CPDPRG[2	waltz16
NUC2	off
PCPD2	100.00 usec
PL2	0 dB
PL12	17.52 dB
SFO2	500.3300000 MHz

F2 - Processing parameters

SI	32768
SF	160.5257010 MHz
WDW	EM
SSB	0
LB	10.00 Hz
GB	0
PC	1.40

11B{1H} NMR

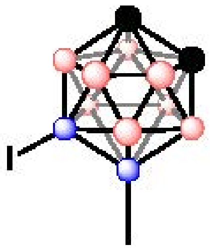

5

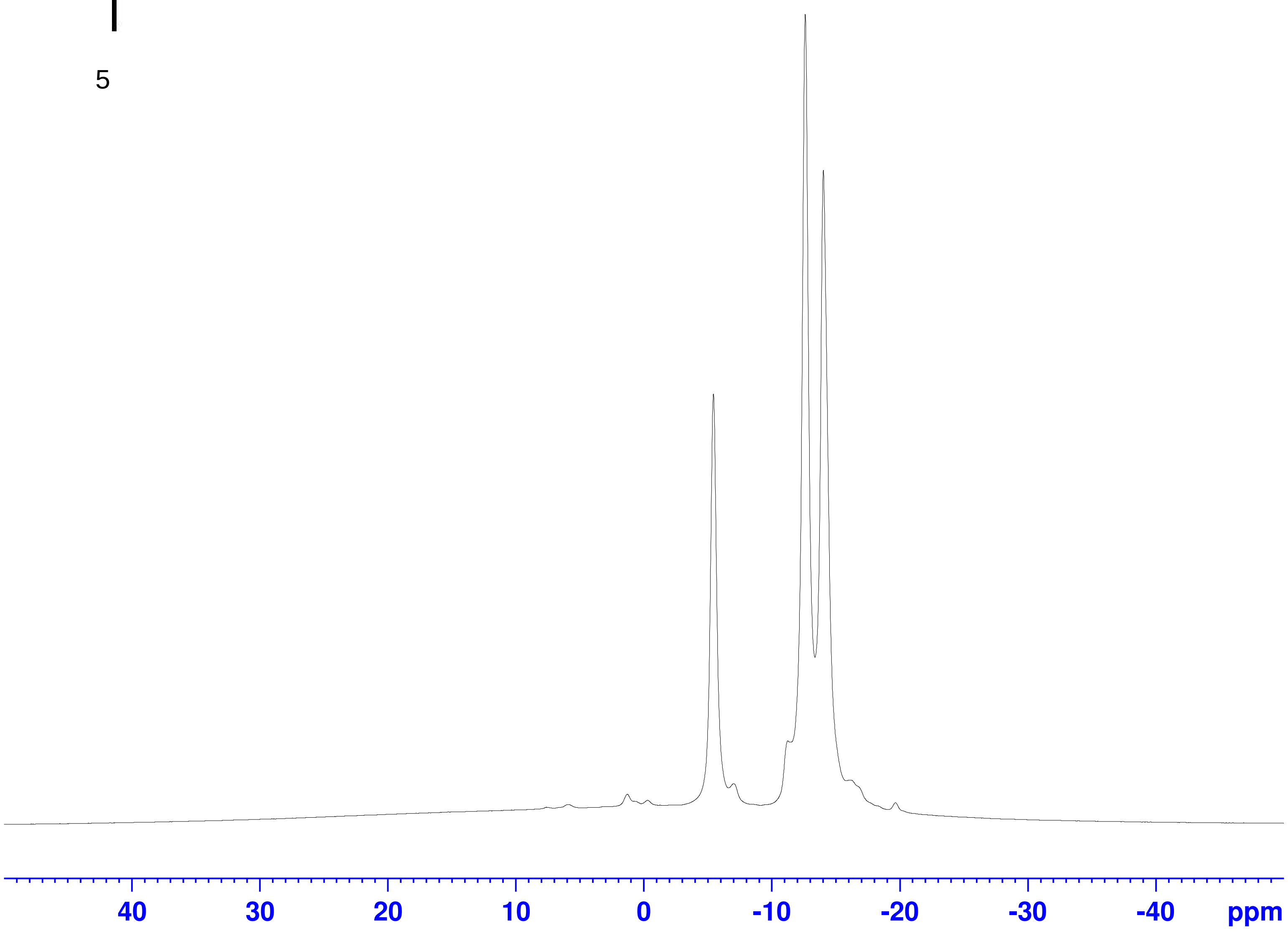


```
Current Data Parameters
NAME        kk-1-264 i2-ocb
EXPNO                     1
PROCNO                    1

F2 - Acquisition Parameters
Date_              20151002
Time                  12.22
INSTRUM              drx500
PROBHD     5 mm bb-Z Z800
PULPROG                zgdc
TD                     7168
SOLVENT                 D2O
NS                     4096
DS                        4
SWH           64102.562 Hz
FIDRES         8.942880 Hz
AQ            0.0559104 sec
RG                     1024
DW                7.800 usec
DE                 6.00 usec
TE                  297.2 K
D1           0.00000400 sec
d11          0.03000000 sec
TD0                       1

======== CHANNEL f1 ========
NUC1                    11B
P1                 8.00 usec
PL1                -2.00 dB
SFO1        160.5257136 MHz

======== CHANNEL f2 ========
CPDPRG[2            waltz16
NUC2                     1H
PCPD2            100.00 usec
PL2        0 dB
PL12               17.52 dB
SFO2        500.3330020 MHz

F2 - Processing parameters
SI                    32768
SF          160.5257013 MHz
WDW                      EM
SSB        0
LB                 10.00 Hz
GB         0
PC                     1.40
```

13C{1H} NMR

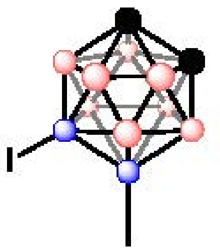

5

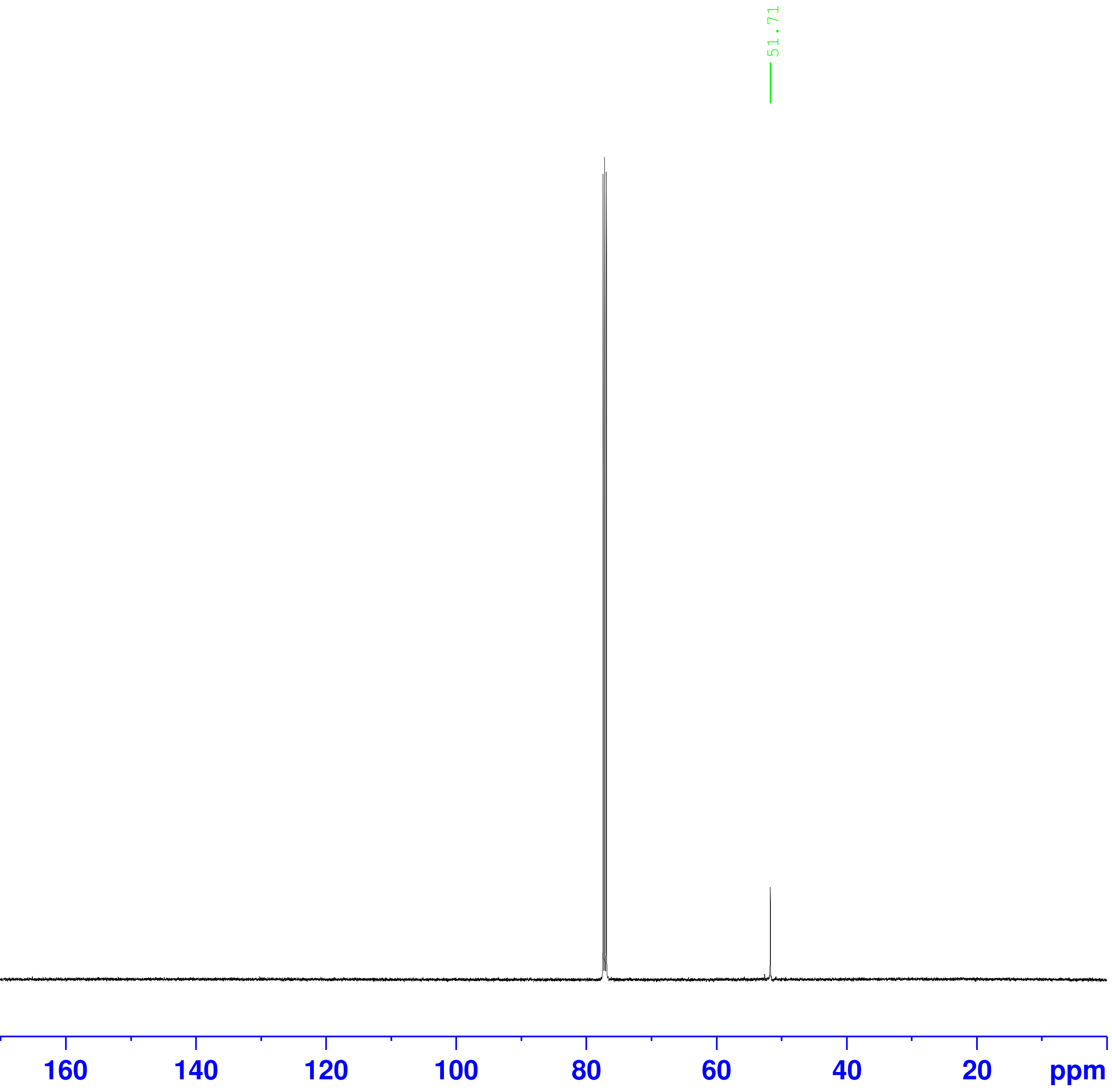


```
Current Data Parameters
NAME       i2-ocb kk-1-265ish
EXPNO                   11
PROCNO                   1

F2 - Acquisition Parameters
Date_             20151002
Time                 11.33
INSTRUM              av500
PROBHD    5 mm DCH 13C-1
PULPROG            zgpg30
TD                  65536
SOLVENT             CDCl3
NS                     32
DS                      2
SWH             31250.000 Hz
FIDRES           0.476837 Hz
AQ              1.0485760 sec
RG                 204.54
DW                 16.000 usec
DE                  18.00 usec
TE                  298.0 K
D1             2.00000000 sec
D11            0.03000000 sec
TD0                     1

======== CHANNEL f1 ========
SFO1          125.7722511 MHz
NUC1                  13C
P1                   9.63 usec
PLW1          23.00000000 W

======== CHANNEL f2 ========
SFO2          500.1330008 MHz
NUC2                   1H
CPDPRG[2          waltz16
PCPD2               80.00 usec
PLW2          13.50000000 W
PLW12          0.21094000 W
PLW13          0.13500001 W

F2 - Processing parameters
SI                 131072
SF            125.7577744 MHz
WDW                    EM
SSB        0
LB                   1.00 Hz
GB         0
PC                   1.40
```

1H NMR

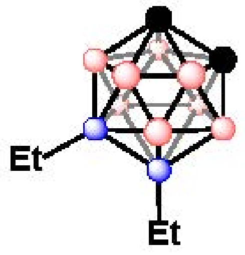

6

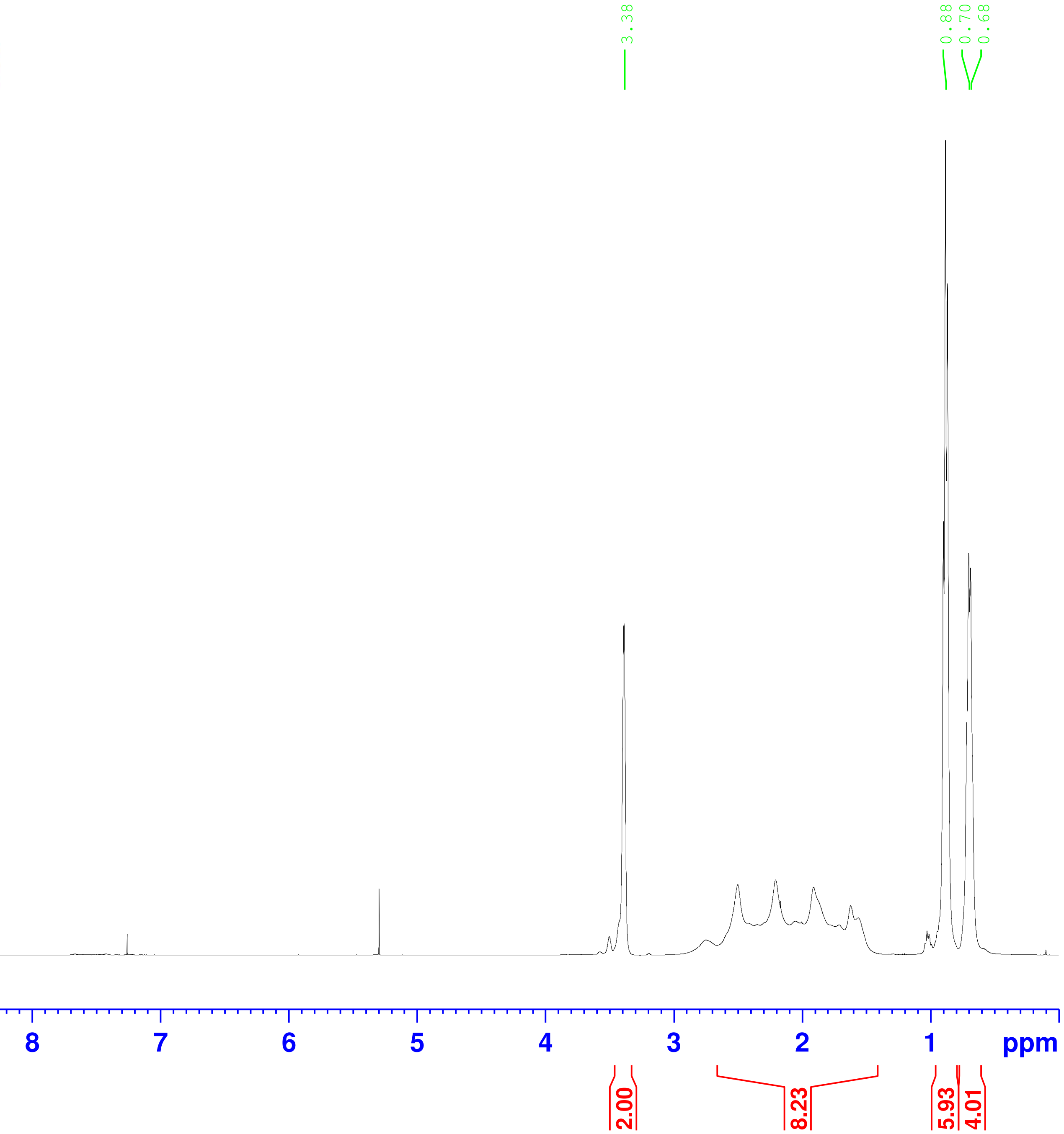


```
Current Data Parameters
NAME      diethyl-o-carborane pure
EXPNO                 10
PROCNO                 1

F2 - Acquisition Parameters
Date_           20151103
Time               17.47
INSTRUM            av500
PROBHD   5 mm DCH 13C-1
PULPROG             zg30
TD                 65536
SOLVENT            CDCl3
NS                    64
DS                     0
SWH         10000.000 Hz
FIDRES       0.152588 Hz
AQ          3.2767999 sec
RG                  3.63
DW             50.000 usec
DE             10.00 usec
TE             298.0 K
D1         2.00000000 sec
TD0                    1

======== CHANNEL f1 ========
SFO1       500.1330008 MHz
NUC1                  1H
P1             10.00 usec
PLW1       13.50000000 W

F2 - Processing parameters
SI                 65536
SF         500.1300123 MHz
WDW                   EM
SSB      0
LB                0.30 Hz
GB       0
PC                  1.00
```

11B NMR

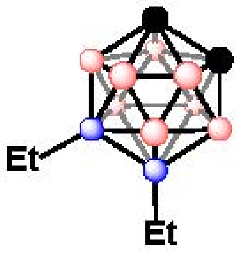

6

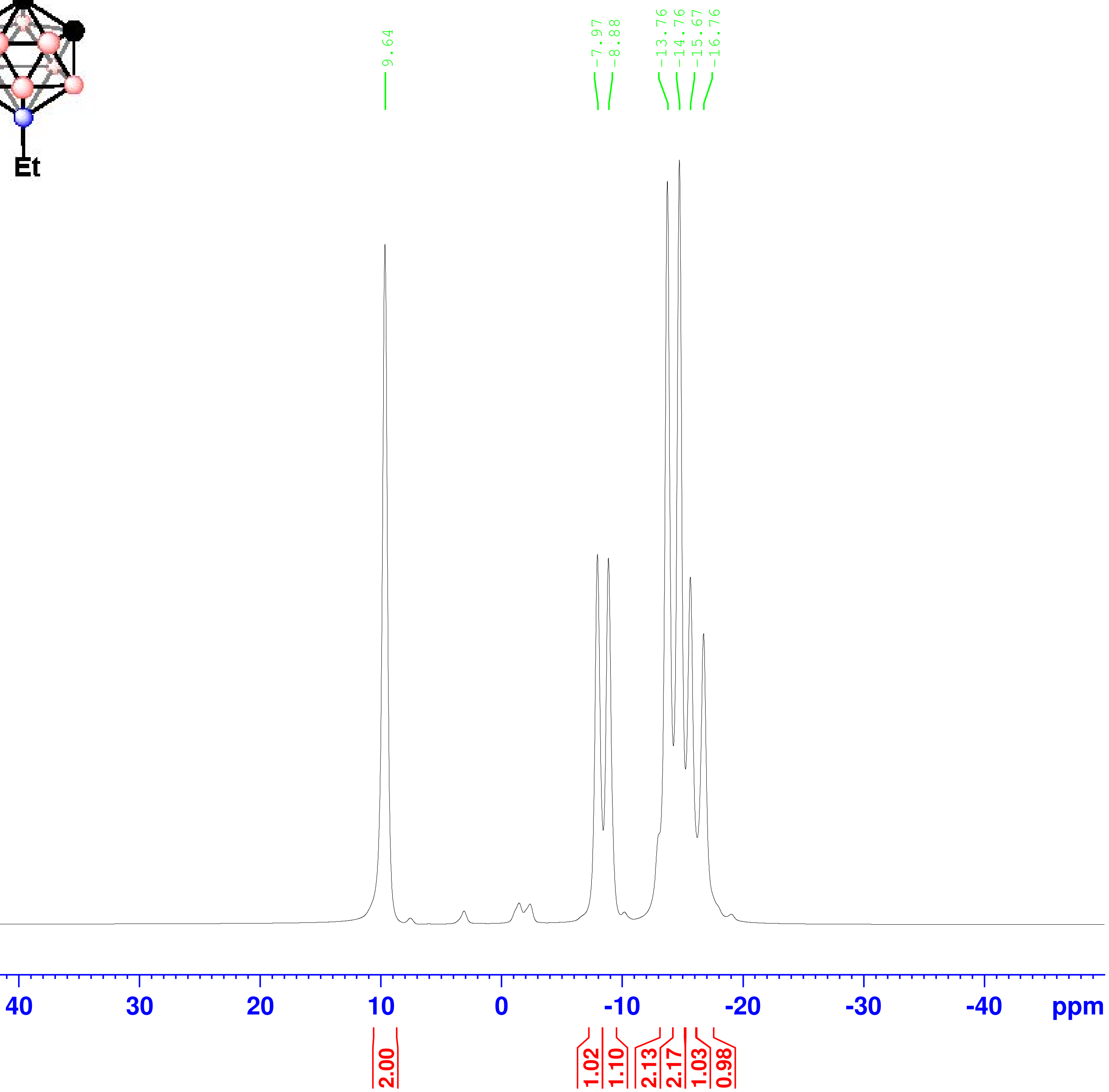


```
Current Data Parameters
NAME     diethyl-o-carborane pure 11b
EXPNO                   2
PROCNO                  1

F2 - Acquisition Parameters
Date_            20151103
Time                20.02
INSTRUM            drx500
PROBHD    5 mm bb-Z Z800
PULPROG                zg
TD                   7168
SOLVENT               D2O
NS                   4096
DS                      4
SWH             64102.562 Hz
FIDRES           8.942880 Hz
AQ              0.0559104 sec
RG                   25.4
DW                  7.800 usec
DE                   6.00 usec
TE                  298.0 K
D1             0.00000400 sec
TD0                     1

======== CHANNEL f1 ========
NUC1                  11B
P1                   8.00 usec
PL1                 -2.00 dB
SFO1          160.5257136 MHz

F2 - Processing parameters
SI                  32768
SF            160.5257136 MHz
WDW                    EM
SSB      0
LB                   1.00 Hz
GB       0
PC                   1.40
```

$^{11}B\{^{1}H\}$ NMR

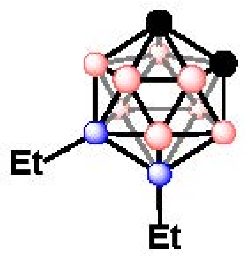

6

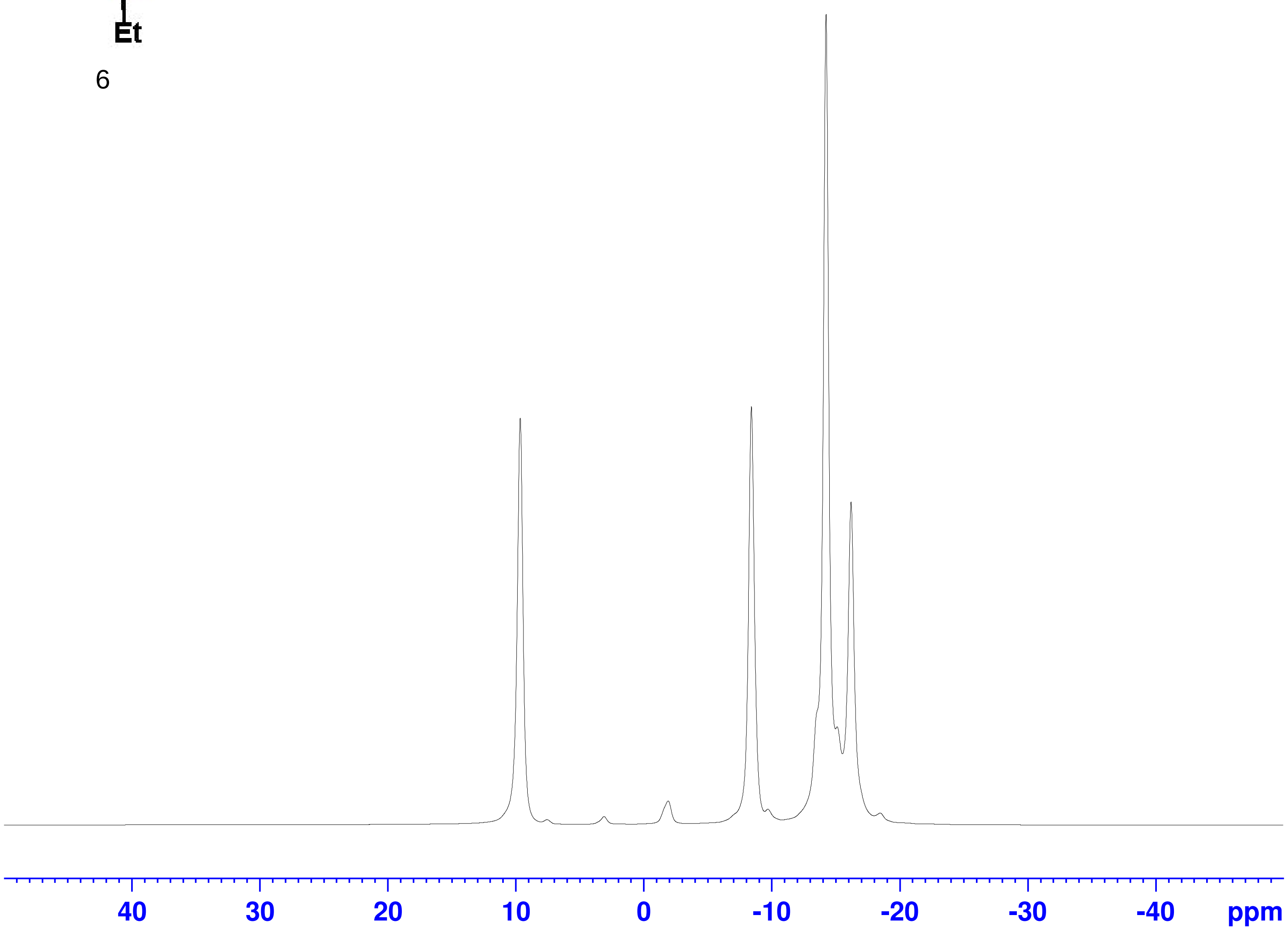


```
Current Data Parameters
NAME     diethyl-o-carborane pure 11b
EXPNO                    1
PROCNO                   1

F2 - Acquisition Parameters
Date_             20151103
Time                 20.15
INSTRUM             drx500
PROBHD    5 mm bb-Z Z800
PULPROG               zgdc
TD                    7168
SOLVENT                D2O
NS                    4096
DS                       4
SWH              64102.562 Hz
FIDRES            8.942880 Hz
AQ               0.0559104 sec
RG                    28.5
DW                   7.800 usec
DE                    6.00 usec
TE                   298.0 K
D1              0.00000400 sec
d11             0.03000000 sec
TD0                      1

======== CHANNEL f1 ========
NUC1                   11B
P1                    8.00 usec
PL1                  -2.00 dB
SFO1           160.5257136 MHz

======== CHANNEL f2 ========
CPDPRG[2           waltz16
NUC2                    1H
PCPD2               100.00 usec
PL2           0 dB
PL12                 17.52 dB
SFO2           500.3330020 MHz

F2 - Processing parameters
SI                   32768
SF             160.5257136 MHz
WDW                     EM
SSB           0
LB                   10.00 Hz
GB            0
PC                    1.40
```

13C{1H} NMR

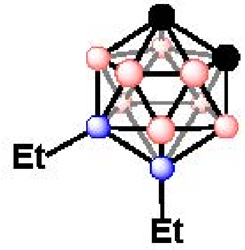

6

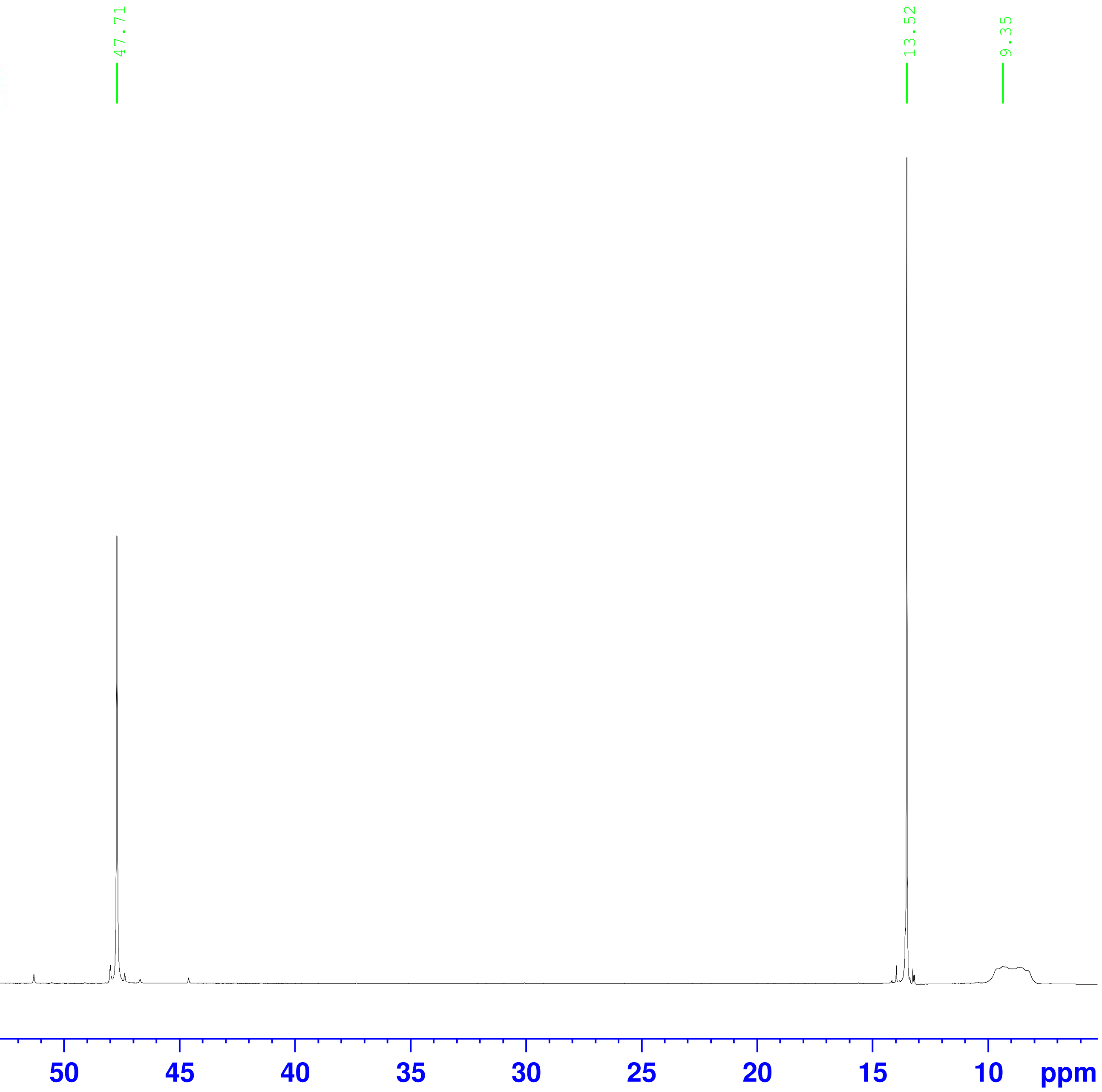


```
Current Data Parameters
NAME      diethyl-o-carborane pure
EXPNO                  11
PROCNO                  1

F2 - Acquisition Parameters
Date_            20151103
Time                18.43
INSTRUM             av500
PROBHD     5 mm DCH 13C-1
PULPROG            zgpg30
TD                  65536
SOLVENT             CDCl3
NS                   1024
DS                      2
SWH             31250.000 Hz
FIDRES           0.476837 Hz
AQ              1.0485760 sec
RG                 204.54
DW                 16.000 usec
DE                  18.00 usec
TE                  298.0 K
D1             2.00000000 sec
D11            0.03000000 sec
TD0                     1

======== CHANNEL f1 ========
SFO1          125.7722511 MHz
NUC1                  13C
P1                   9.63 usec
PLW1          23.00000000 W

======== CHANNEL f2 ========
SFO2          500.1330008 MHz
NUC2                   1H
CPDPRG[2          waltz16
PCPD2               80.00 usec
PLW2          13.50000000 W
PLW12          0.21094000 W
PLW13          0.13500001 W

F2 - Processing parameters
SI                 131072
SF            125.7577809 MHz
WDW                    EM
SSB      0
LB                   1.00 Hz
GB       0
PC                   1.40
```

1H NMR

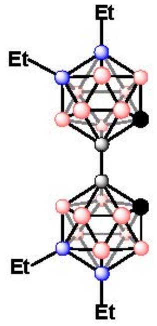

7

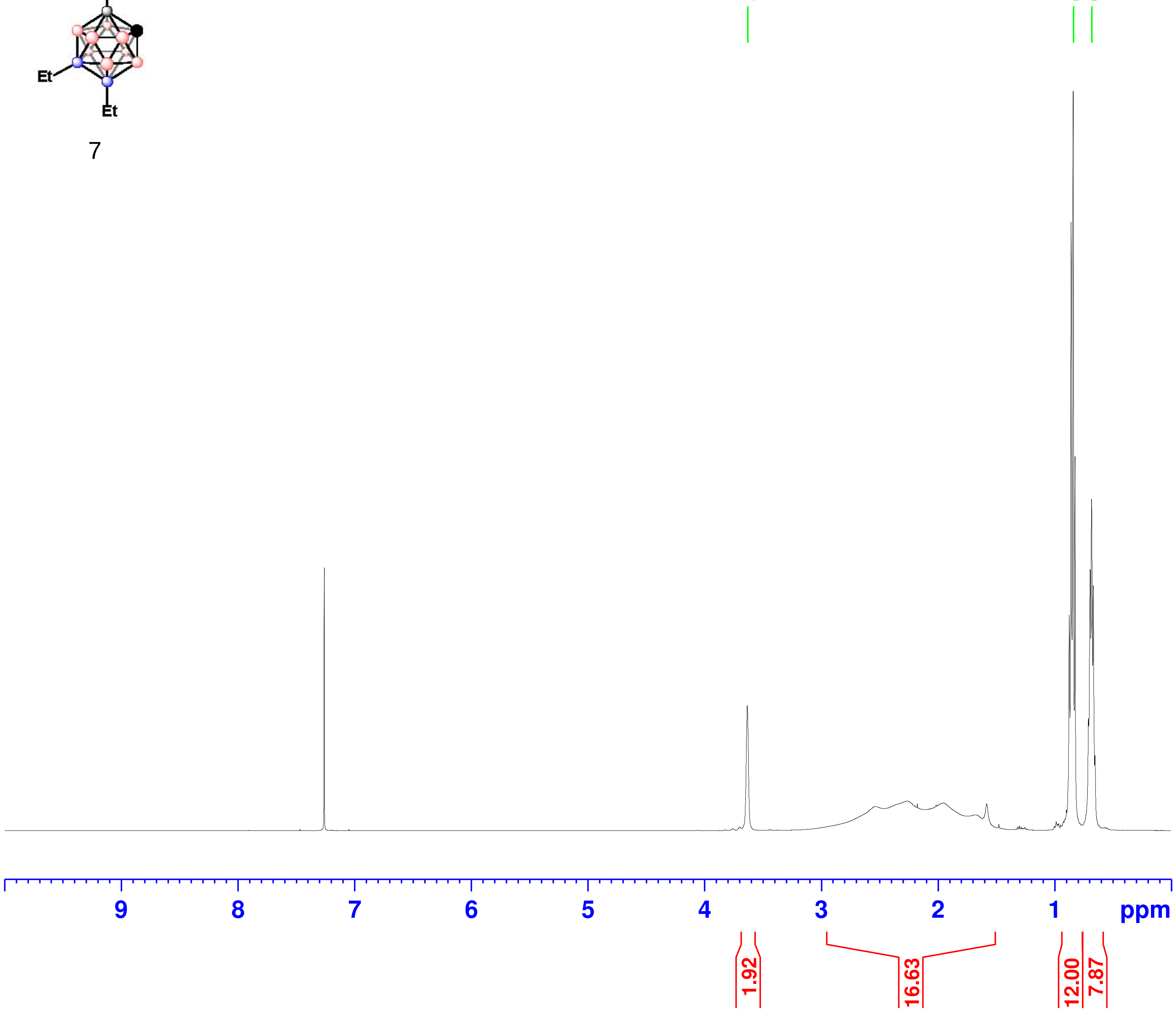

Current Data Parameters
NAME kk-1-210 tebc
EXPNO 10
PROCNO 1

F2 - Acquisition Parameters
Date_ 20151012
Time 17.06
INSTRUM av500
PROBHD 5 mm DCH 13C-1
PULPROG zg30
TD 65536
SOLVENT CDCl3
NS 128
DS 0
SWH 10000.000 Hz
FIDRES 0.152588 Hz
AQ 3.2767999 sec
RG 12.14
DW 50.000 usec
DE 10.00 usec
TE 298.0 K
D1 2.00000000 sec
TD0 1

======== CHANNEL f1 ========
SFO1 500.1330008 MHz
NUC1 1H
P1 10.00 usec
PLW1 13.50000000 W

F2 - Processing parameters
SI 65536
SF 500.1300121 MHz
WDW EM
SSB 0
LB 0.30 Hz
GB 0
PC 1.00

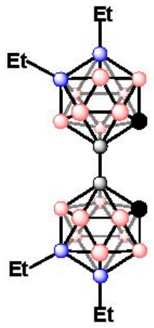

7

11B NMR

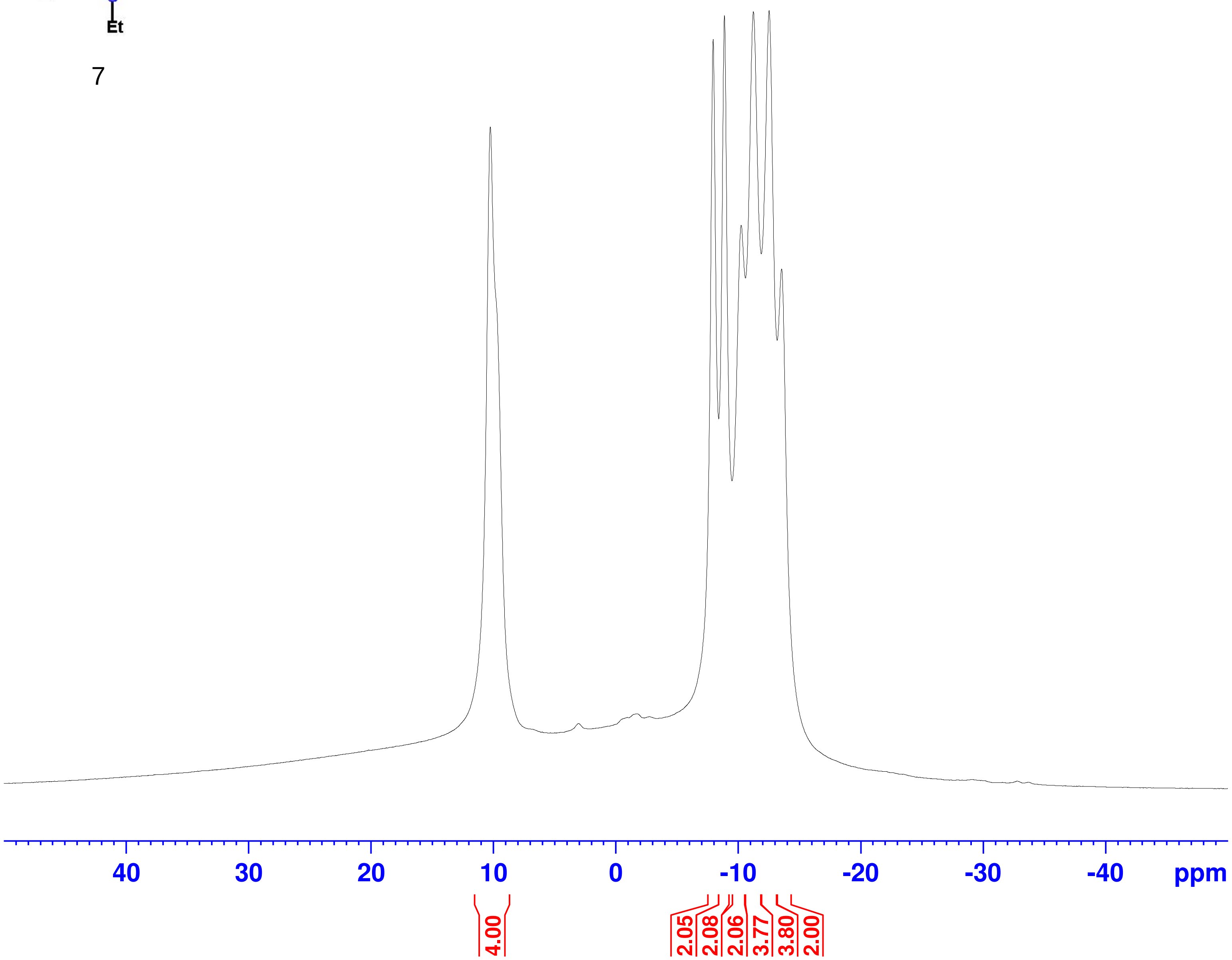

Current Data Parameters

NAME	tetraethyl-bis-carborane pure 11b
EXPNO	2
PROCNO	1

F2 - Acquisition Parameters

Date_	20151111
Time	16.24
INSTRUM	drx500
PROBHD	5 mm bb-Z Z800
PULPROG	zgdc
TD	7168
SOLVENT	D2O
NS	2048
DS	4
SWH	64102.562 Hz
FIDRES	8.942880 Hz
AQ	0.0559104 sec
RG	322.5
DW	7.800 usec
DE	6.00 usec
TE	298.0 K
D1	0.00000400 sec
d11	0.03000000 sec
TD0	1

======== CHANNEL f1 ========

NUC1	11B
P1	8.00 usec
PL1	-2.00 dB
SFO1	160.5257140 MHz

======== CHANNEL f2 ========

CPDPRG[2	waltz16
NUC2	off
PCPD2	100.00 usec
PL2	0 dB
PL12	17.52 dB
SFO2	500.3300000 MHz

F2 - Processing parameters

SI	32768
SF	160.5257136 MHz
WDW	EM
SSB	0
LB	10.00 Hz
GB	0
PC	1.40

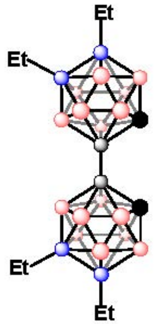

7

11B{1H} NMR

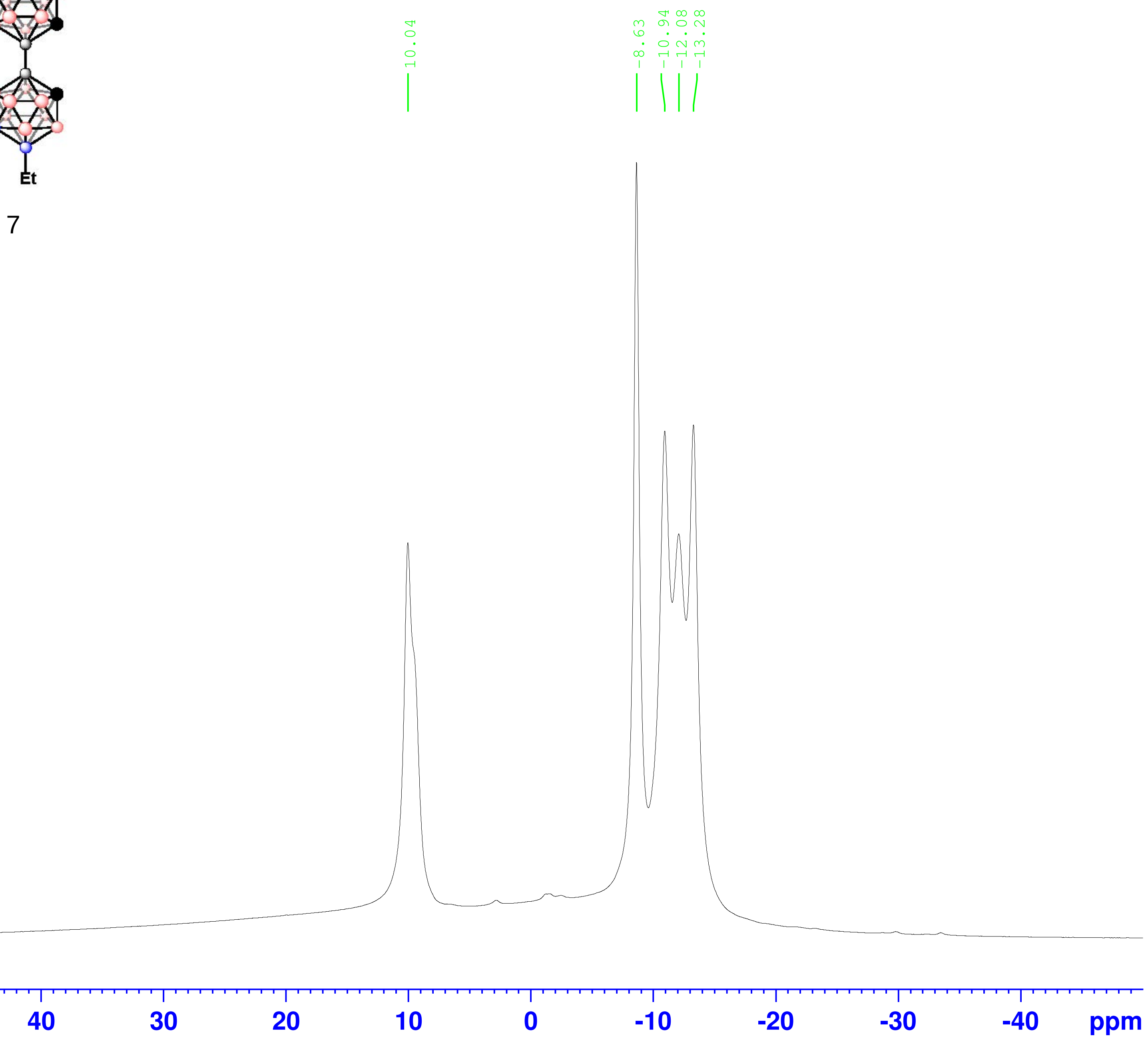

Current Data Parameters
NAME tetraethyl-bis-carborane pure 11b
EXPNO 1
PROCNO 1

F2 - Acquisition Parameters
Date_ 20151111
Time 16.19
INSTRUM drx500
PROBHD 5 mm bb-Z Z800
PULPROG zgdc
TD 7168
SOLVENT D2O
NS 2048
DS 4
SWH 64102.562 Hz
FIDRES 8.942880 Hz
AQ 0.0559104 sec
RG 322.5
DW 7.800 usec
DE 6.00 usec
TE 298.1 K
D1 0.00000400 sec
d11 0.03000000 sec
TD0 1

======== CHANNEL f1 ========
NUC1 11B
P1 8.00 usec
PL1 -2.00 dB
SFO1 160.5257136 MHz

======== CHANNEL f2 ========
CPDPRG[2 waltz16
NUC2 1H
PCPD2 100.00 usec
PL2 0 dB
PL12 17.52 dB
SFO2 500.3330020 MHz

F2 - Processing parameters
SI 32768
SF 160.5257476 MHz
WDW EM
SSB 0
LB 10.00 Hz
GB 0
PC 1.40

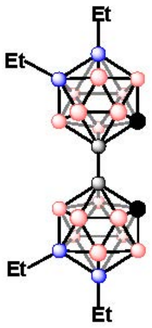

7

13C{1H} NMR

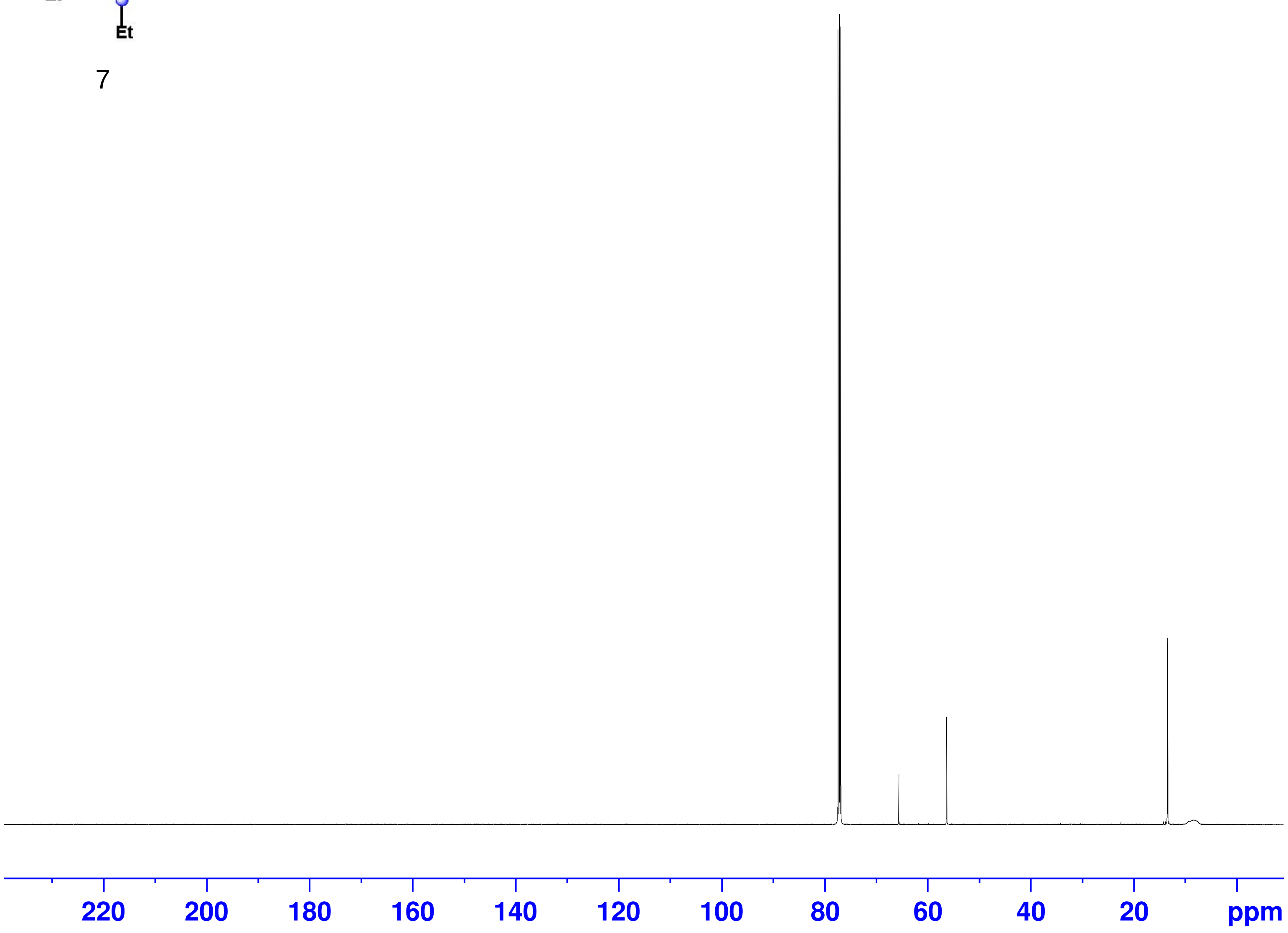


```
Current Data Parameters
NAME          kk-1-210 tebc
EXPNO                    11
PROCNO                    1

F2 - Acquisition Parameters
Date_              20151012
Time                  18.10
INSTRUM               av500
PROBHD    5 mm DCH 13C-1
PULPROG             zgpg30
TD                    65536
SOLVENT               CDCl3
NS                     1024
DS                        2
SWH           31250.000 Hz
FIDRES         0.476837 Hz
AQ            1.0485760 sec
RG                   204.54
DW               16.000 usec
DE                18.00 usec
TE                298.0 K
D1           2.00000000 sec
D11          0.03000000 sec
TD0                       1

======== CHANNEL f1 ========
SFO1        125.7722511 MHz
NUC1                    13C
P1                 9.63 usec
PLW1        23.00000000 W

======== CHANNEL f2 ========
SFO2        500.1330008 MHz
NUC2                     1H
CPDPRG[2            waltz16
PCPD2              80.00 usec
PLW2        13.50000000 W
PLW12        0.21094000 W
PLW13        0.13500001 W

F2 - Processing parameters
SI                   131072
SF          125.7577716 MHz
WDW                      EM
SSB       0
LB                  1.00 Hz
GB        0
PC                     1.40
```

1H NMR

8

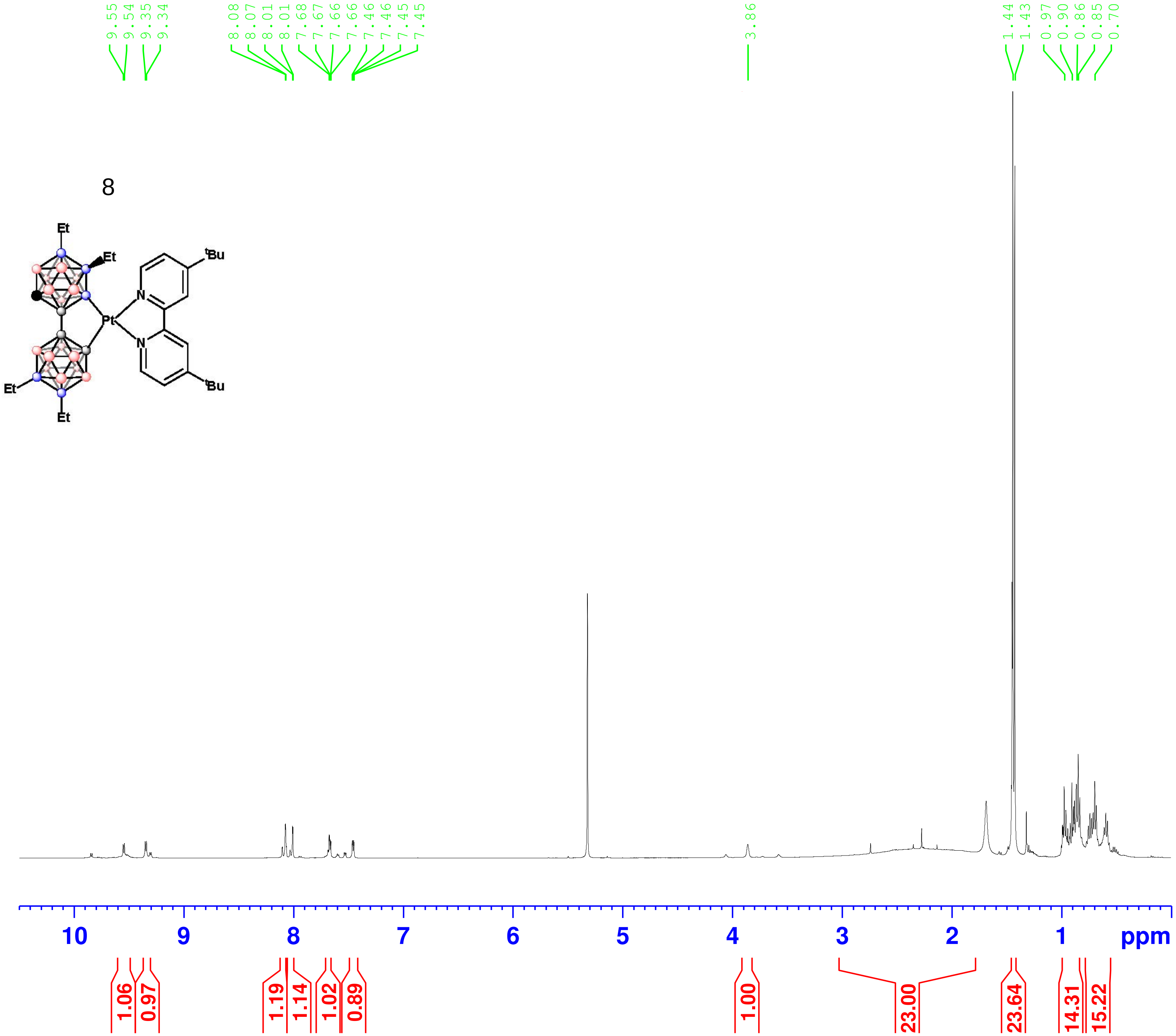


```
Current Data Parameters
NAME      kk-1-232 pt(tebc)(dtb-bpy)
EXPNO               10
PROCNO               1

F2 - Acquisition Parameters
Date_          20150922
Time              11.09
INSTRUM           av500
PROBHD    5 mm DCH 13C-1
PULPROG            zg30
TD                65536
SOLVENT          CD2Cl2
NS                   64
DS                    0
SWH           10000.000 Hz
FIDRES         0.152588 Hz
AQ            3.2767999 sec
RG                12.14
DW               50.000 usec
DE                10.00 usec
TE                298.0 K
D1           2.00000000 sec
TD0                   1

======== CHANNEL f1 ========
SFO1       500.1330008 MHz
NUC1                 1H
P1                10.00 usec
PLW1        13.50000000 W

F2 - Processing parameters
SI                65536
SF          500.1300194 MHz
WDW                  EM
SSB       0
LB                 0.30 Hz
GB        0
PC                 1.00
```

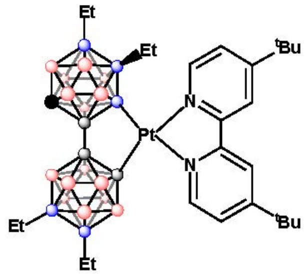

8

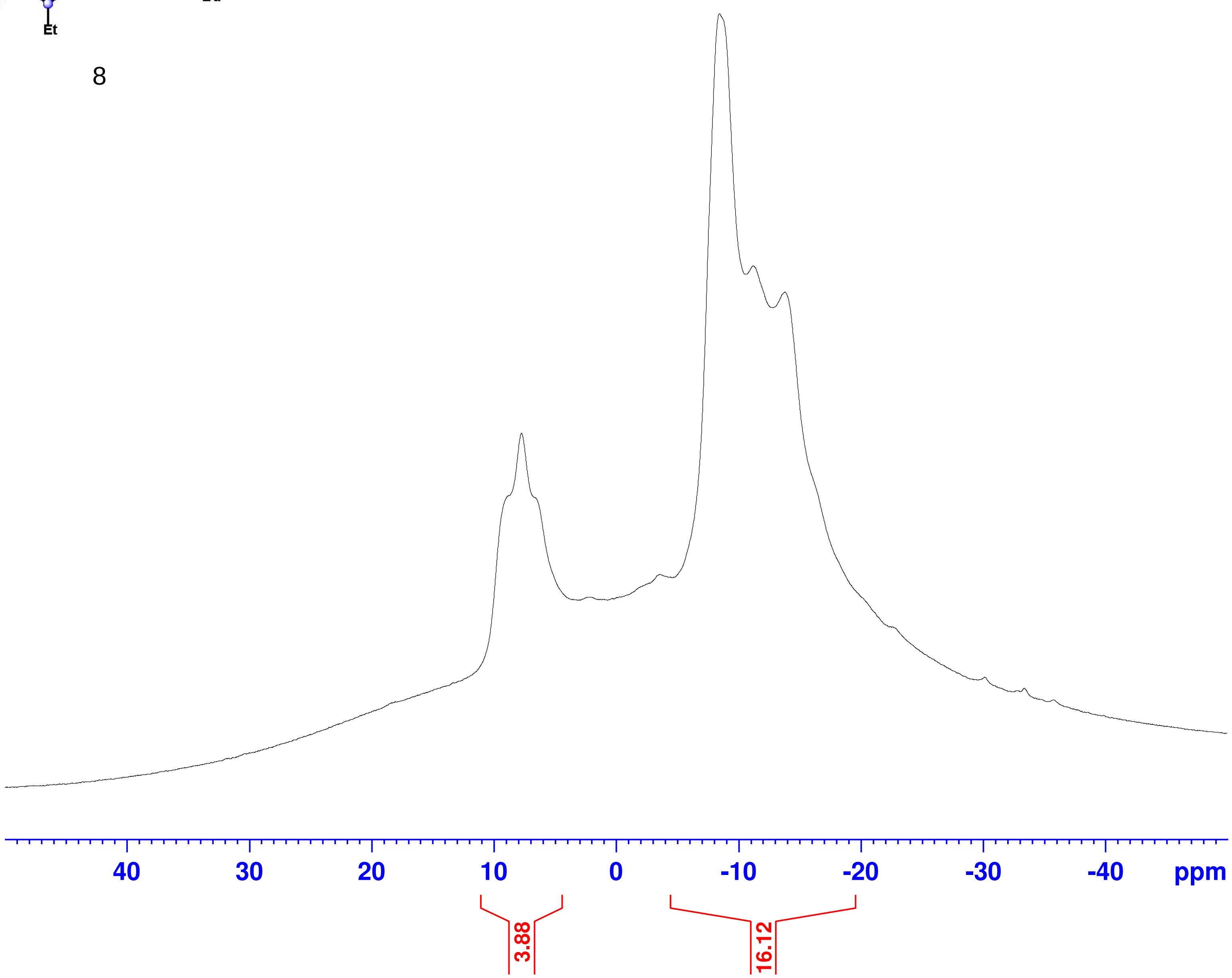


```
Current Data Parameters
NAME      KK-1-232 Pt(tebc)(dtb-bpy) pure 11B
EXPNO                    1
PROCNO                   1

F2 - Acquisition Parameters
Date_             20150922
Time                 14.05
INSTRUM             drx500
PROBHD     5 mm bb-Z Z800
PULPROG               zgdc
TD                    7168
SOLVENT             CD2Cl2
NS                    4096
DS                       4
SWH              64102.562 Hz
FIDRES            8.942880 Hz
AQ               0.0559104 sec
RG                   574.7
DW                   7.800 usec
DE                    6.00 usec
TE                   297.2 K
D1              0.00000400 sec
d11             0.03000000 sec
TD0                      1

======== CHANNEL f1 ========
NUC1                   11B
P1                    8.00 usec
PL1                  -2.00 dB
SFO1           160.5257136 MHz

======== CHANNEL f2 ========
CPDPRG[2           waltz16
NUC2                    1H
PCPD2               100.00 usec
PL2         0 dB
PL12                 17.52 dB
SFO2           500.3330020 MHz

F2 - Processing parameters
SI                   32768
SF             160.5257717 MHz
WDW                     EM
SSB         0
LB                   10.00 Hz
GB          0
PC                    1.40
```

13C{1H} NMR

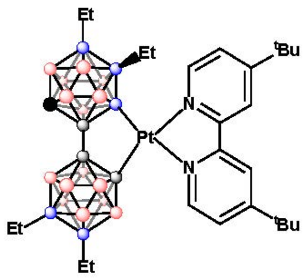

8

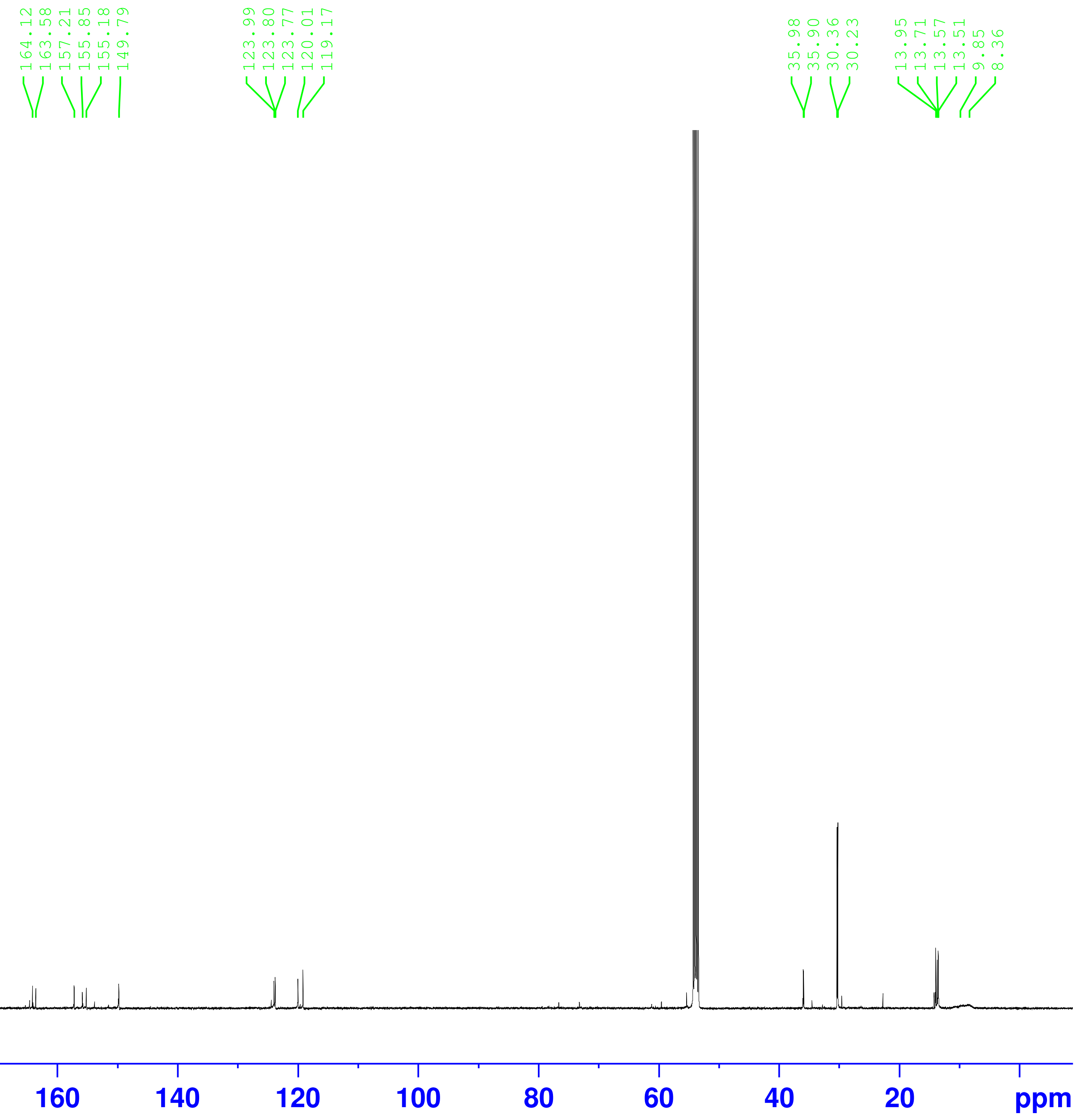


```
Current Data Parameters
NAME      kk-1-232 pt(tebc)(dtb-bpy)
EXPNO                 11
PROCNO                 1

F2 - Acquisition Parameters
Date_           20150922
Time               12.03
INSTRUM            av500
PROBHD    5 mm DCH 13C-1
PULPROG          zgpg30
TD                 65536
SOLVENT           CD2Cl2
NS                  1024
DS                     2
SWH            31250.000 Hz
FIDRES          0.476837 Hz
AQ             1.0485760 sec
RG                204.54
DW                16.000 usec
DE                 18.00 usec
TE                 298.0 K
D1            2.00000000 sec
D11           0.03000000 sec
TD0                    1

======== CHANNEL f1 ========
SFO1         125.7722511 MHz
NUC1                 13C
P1                  9.63 usec
PLW1         23.00000000 W

======== CHANNEL f2 ========
SFO2         500.1330008 MHz
NUC2                  1H
CPDPRG[2         waltz16
PCPD2              80.00 usec
PLW2         13.50000000 W
PLW12         0.21094000 W
PLW13         0.13500001 W

F2 - Processing parameters
SI                131072
SF           125.7577377 MHz
WDW                   EM
SSB       0
LB                  1.00 Hz
GB        0
PC                  1.40
```

1H NMR

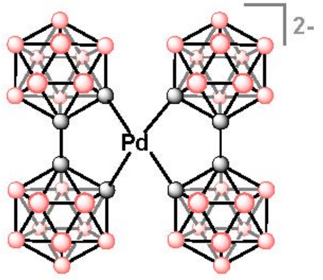

[Pd(bc)2]2-

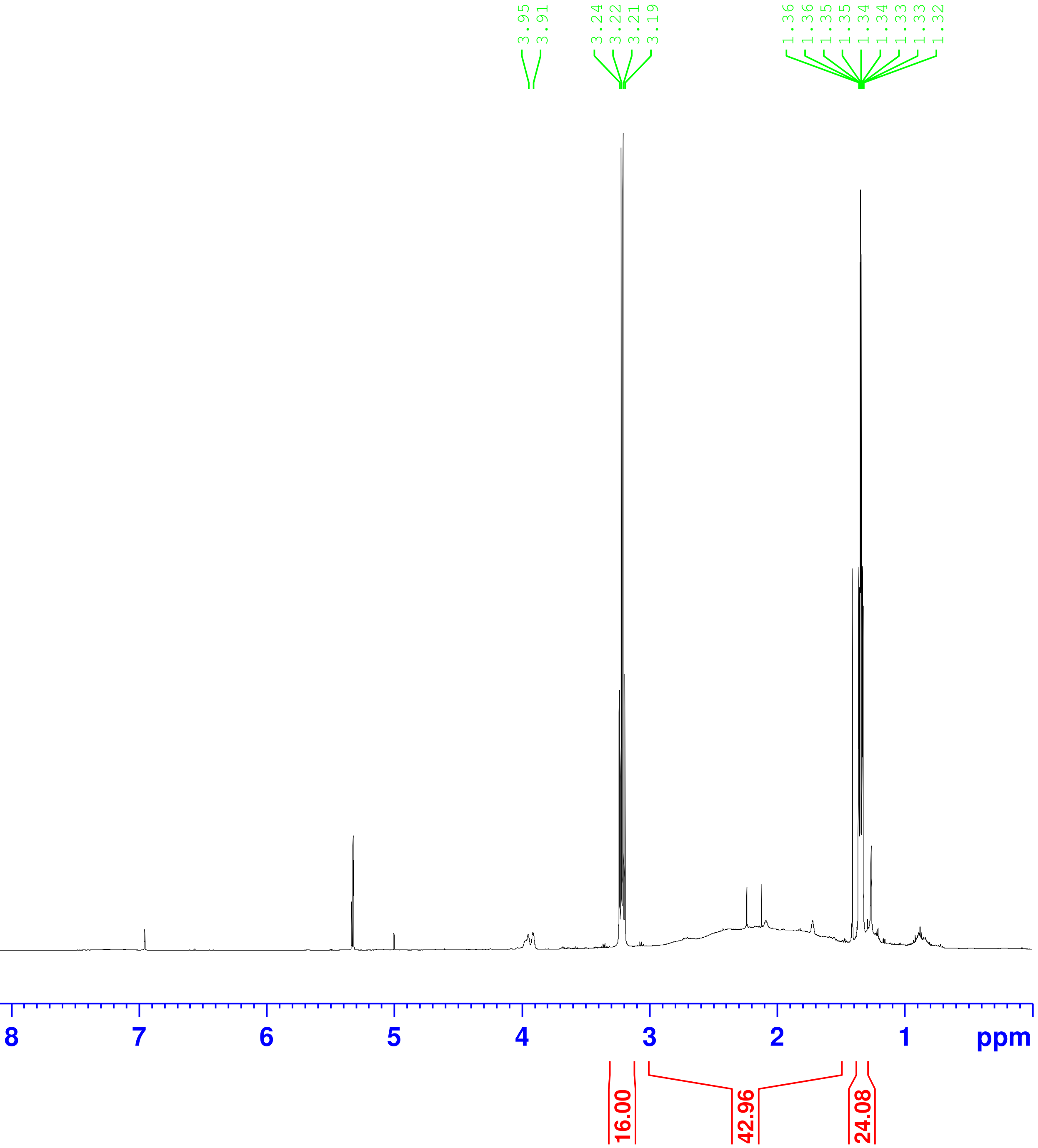


```
Current Data Parameters
NAME      (TEA)2[Pd(bc)2] 1H:13C
EXPNO                  10
PROCNO                  1

F2 - Acquisition Parameters
Date_            20160126
Time                22.53
INSTRUM             av500
PROBHD     5 mm DCH 13C-1
PULPROG              zg30
TD                  65536
SOLVENT            CD2Cl2
NS                     64
DS                      0
SWH             10000.000 Hz
FIDRES           0.152588 Hz
AQ              3.2767999 sec
RG                  12.14
DW                 50.000 usec
DE                  10.00 usec
TE                  298.0 K
D1             2.00000000 sec
TD0                     1

======== CHANNEL f1 ========
SFO1          500.1330008 MHz
NUC1                   1H
P1                  10.00 usec
PLW1          13.50000000 W

F2 - Processing parameters
SI                  65536
SF            500.1300194 MHz
WDW                    EM
SSB        0
LB                   0.30 Hz
GB         0
PC                   1.00
```

11B NMR

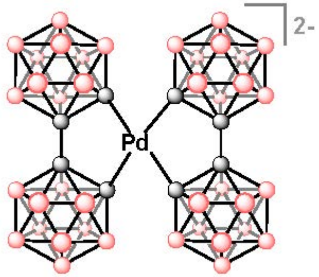

[Pd(bc)2]2-

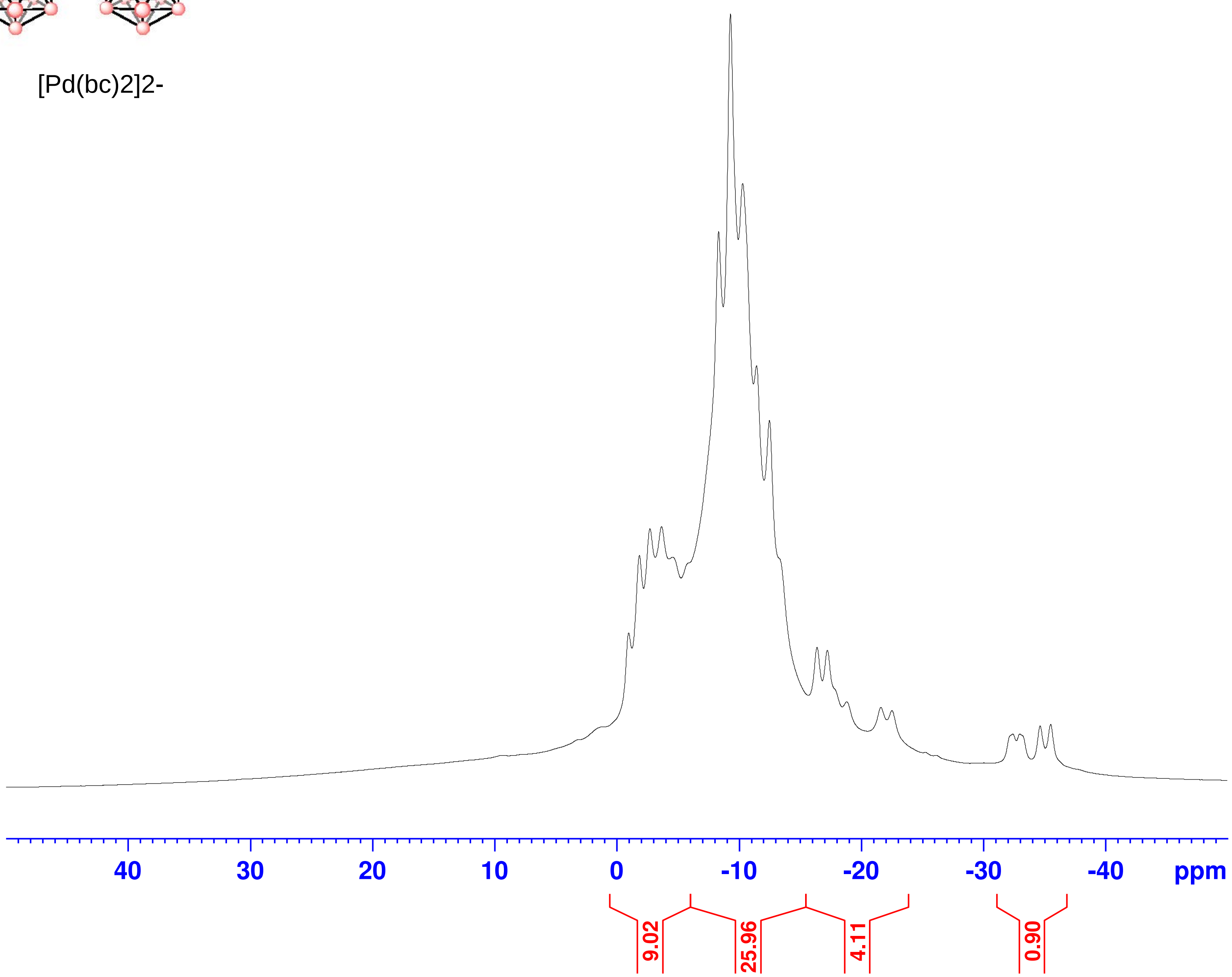


```
Current Data Parameters
NAME      KK-2-022 [Pd(bc)2]2- 11B
EXPNO                        3
PROCNO                       1

F2 - Acquisition Parameters
Date_              20160208
Time                  18.23
INSTRUM              drx500
PROBHD     5 mm bb-Z Z800
PULPROG                zgdc
TD                     7168
SOLVENT              CD2Cl2
NS                     8192
DS                        4
SWH           64102.562 Hz
FIDRES         8.942880 Hz
AQ            0.0559104 sec
RG                    287.4
DW                7.800 usec
DE                 6.00 usec
TE                  297.0 K
D1           0.00000400 sec
d11          0.03000000 sec
TD0                       1

======== CHANNEL f1 ========
NUC1                    11B
P1                 8.00 usec
PL1                -2.00 dB
SFO1        160.5257140 MHz

======== CHANNEL f2 ========
CPDPRG[2            waltz16
NUC2                    off
PCPD2            100.00 usec
PL2        0 dB
PL12               17.52 dB
SFO2        500.3300000 MHz

F2 - Processing parameters
SI                    32768
SF          160.5256716 MHz
WDW                      EM
SSB        0
LB                 10.00 Hz
GB         0
PC                     1.40
```

11B{1H} NMR

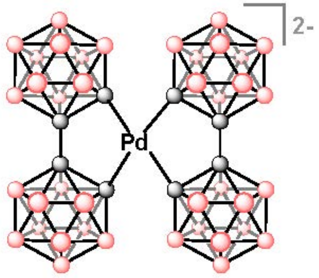

[Pd(bc)2]2-

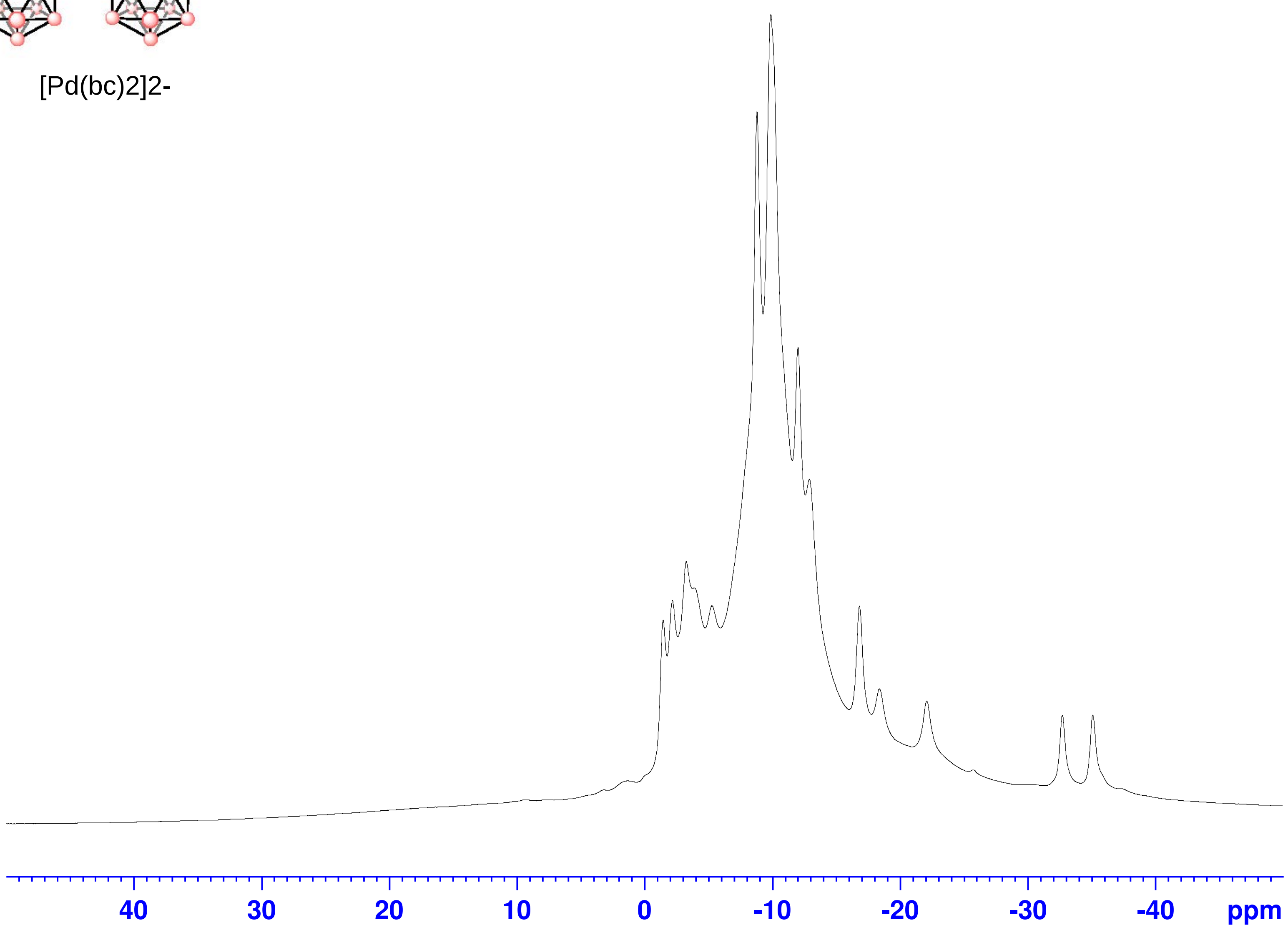

BRUKER

Current Data Parameters
NAME KK-2-022 [Pd(bc)2]2- 11B
EXPNO 2
PROCNO 1

F2 - Acquisition Parameters
Date_ 20160208
Time 18.05
INSTRUM drx500
PROBHD 5 mm bb-Z Z800
PULPROG zgdc
TD 7168
SOLVENT CD2Cl2
NS 4096
DS 4
SWH 64102.562 Hz
FIDRES 8.942880 Hz
AQ 0.0559104 sec
RG 574.7
DW 7.800 usec
DE 6.00 usec
TE 297.2 K
D1 0.00000400 sec
d11 0.03000000 sec
TD0 1

======== CHANNEL f1 ========
NUC1 11B
P1 8.00 usec
PL1 -2.00 dB
SFO1 160.5257136 MHz

======== CHANNEL f2 ========
CPDPRG[2 waltz16
NUC2 1H
PCPD2 100.00 usec
PL2 0 dB
PL12 17.52 dB
SFO2 500.3330020 MHz

F2 - Processing parameters
SI 32768
SF 160.5256716 MHz
WDW EM
SSB 0
LB 10.00 Hz
GB 0
PC 1.40

13C{1H} NMR

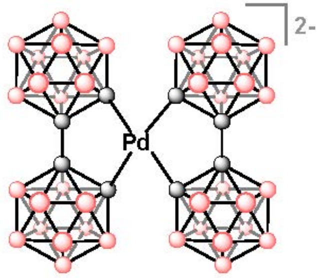

[Pd(bc)2]2-

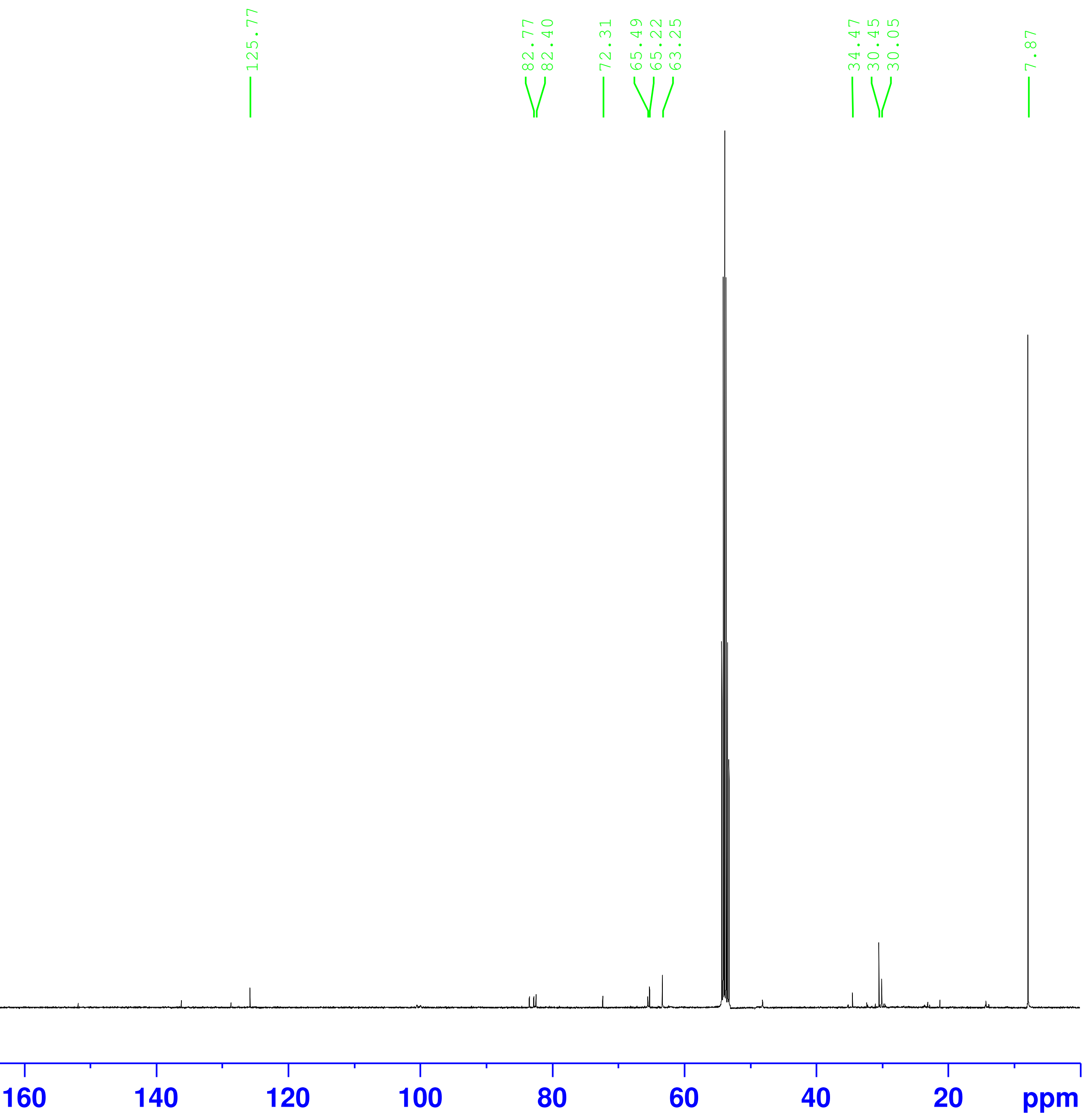

BRUKER

```
Current Data Parameters
NAME      (TEA)2[Pd(bc)2] 1H:13C
EXPNO                     11
PROCNO                     1

F2 - Acquisition Parameters
Date_               20160126
Time                   23.50
INSTRUM                av500
PROBHD     5 mm DCH 13C-1
PULPROG              zgpg30
TD                     65536
SOLVENT               CD2Cl2
NS                      1024
DS                         2
SWH             31250.000 Hz
FIDRES           0.476837 Hz
AQ              1.0485760 sec
RG                    204.54
DW                16.000 usec
DE                 18.00 usec
TE                 298.0 K
D1            2.00000000 sec
D11           0.03000000 sec
TD0                        1

======== CHANNEL f1 ========
SFO1          125.7722511 MHz
NUC1                     13C
P1                  9.63 usec
PLW1         23.00000000 W

======== CHANNEL f2 ========
SFO2          500.1330008 MHz
NUC2                      1H
CPDPRG[2             waltz16
PCPD2              80.00 usec
PLW2         13.50000000 W
PLW12         0.21094000 W
PLW13         0.13500001 W

F2 - Processing parameters
SI                    131072
SF            125.7577413 MHz
WDW                       EM
SSB       0
LB                   1.00 Hz
GB        0
PC                      1.40
```

1H NMR

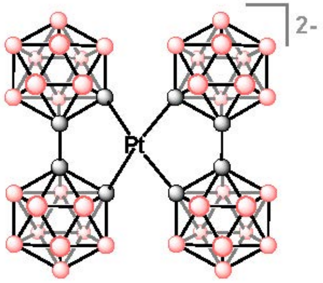

[Pt(bc)2]2-

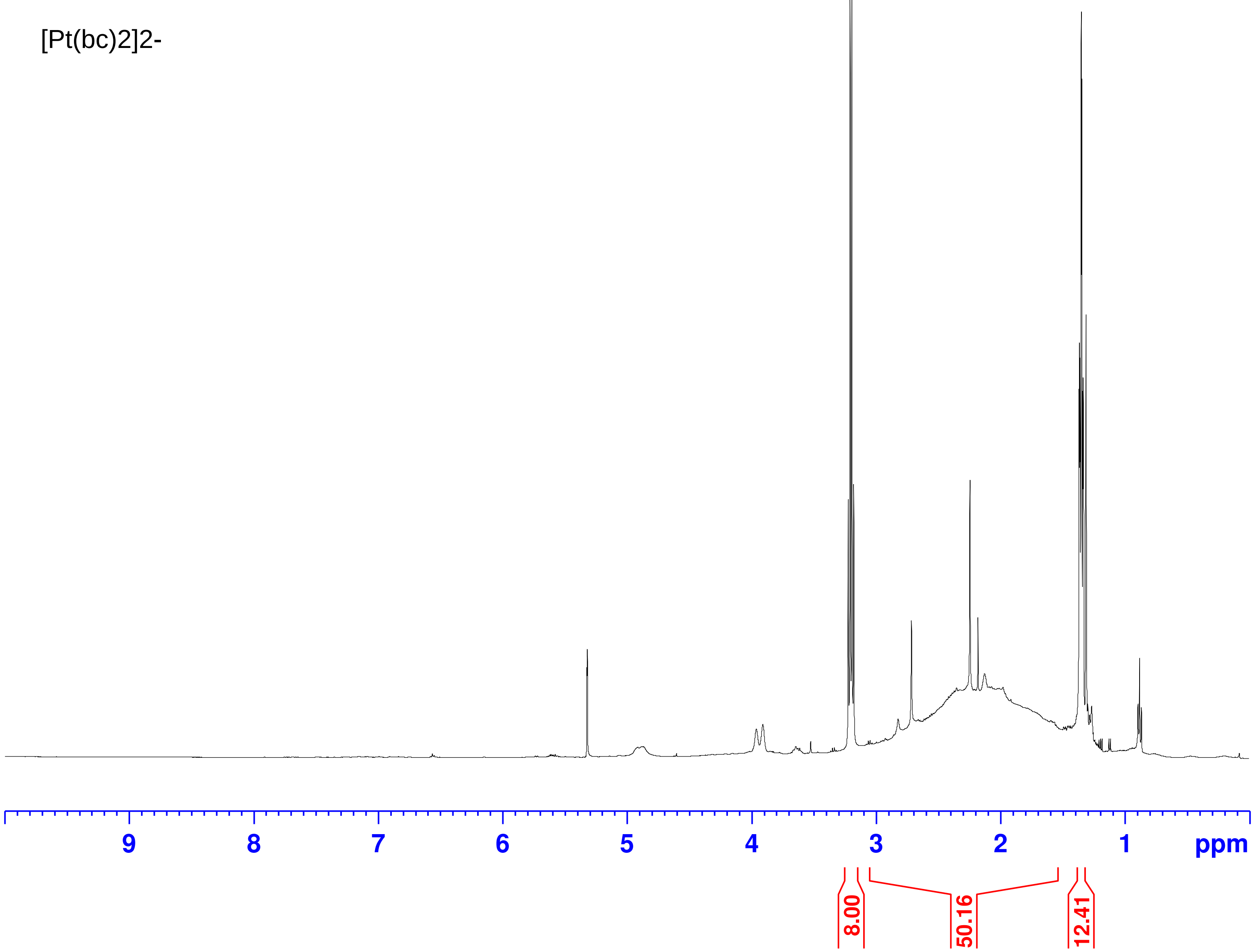

Current Data Parameters
NAME KK-2-017 Pt(bc)2 vac dried
EXPNO 10
PROCNO 1

F2 - Acquisition Parameters
Date_ 20160205
Time 17.10
INSTRUM av500
PROBHD 5 mm DCH 13C-1
PULPROG zg30
TD 65536
SOLVENT CD2Cl2
NS 64
DS 0
SWH 10000.000 Hz
FIDRES 0.152588 Hz
AQ 3.2767999 sec
RG 12.14
DW 50.000 usec
DE 10.00 usec
TE 298.0 K
D1 2.00000000 sec
TD0 1

======== CHANNEL f1 ========
SFO1 500.1330008 MHz
NUC1 1H
P1 10.00 usec
PLW1 13.50000000 W

F2 - Processing parameters
SI 65536
SF 500.1300193 MHz
WDW EM
SSB 0
LB 0.30 Hz
GB 0
PC 1.00

11B NMR

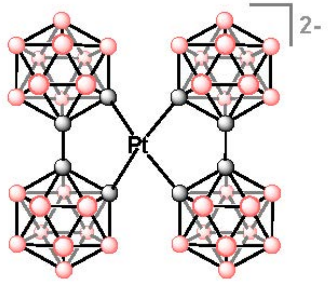

[Pt(bc)2]2-

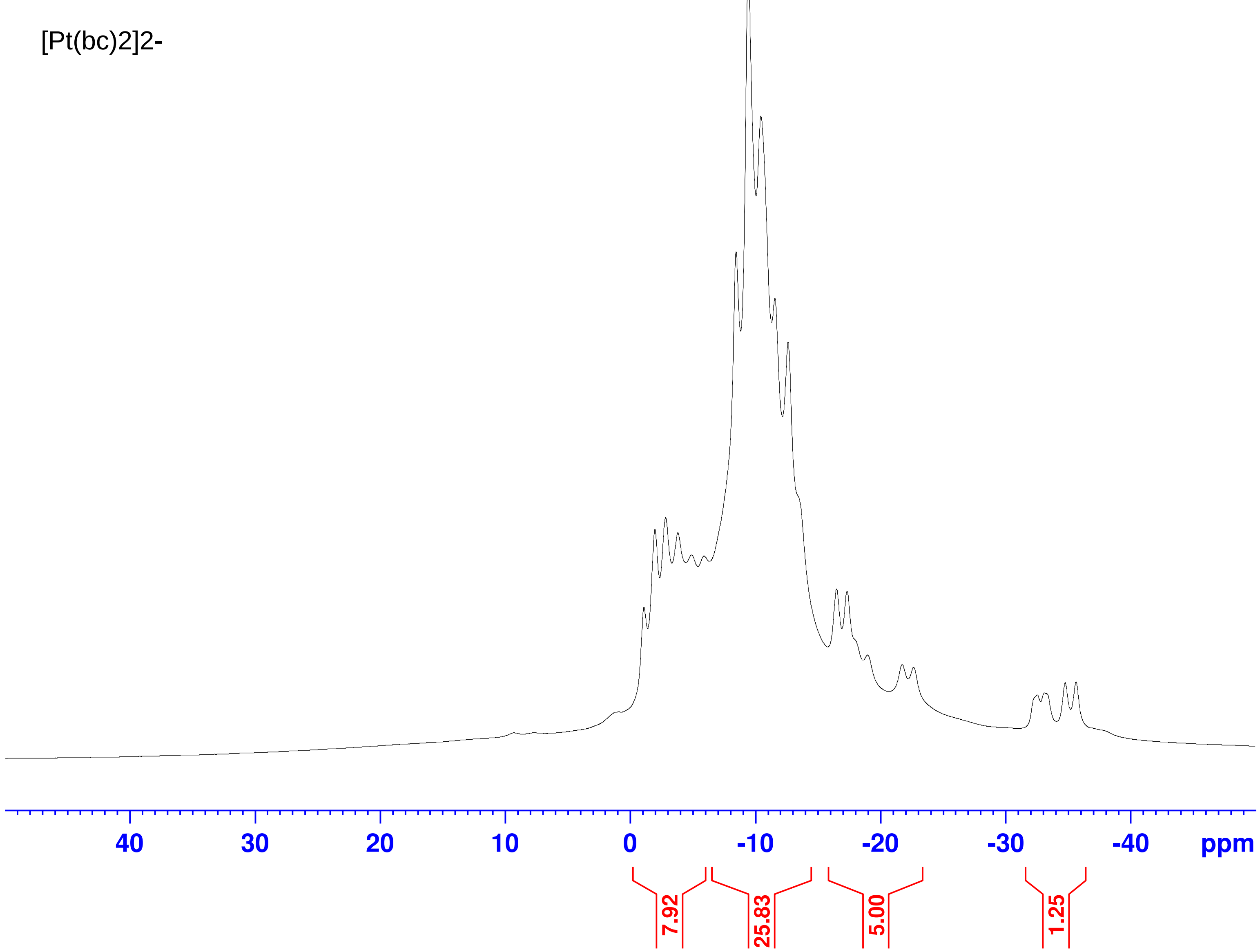

Current Data Parameters
NAME KK-2-017 [Pt(bc)2]2- 11B
EXPNO 3
PROCNO 1

F2 - Acquisition Parameters
Date_ 20160206
Time 17.39
INSTRUM drx500
PROBHD 5 mm bb-Z Z800
PULPROG zgdc
TD 7168
SOLVENT CD2Cl2
NS 8192
DS 4
SWH 64102.562 Hz
FIDRES 8.942880 Hz
AQ 0.0559104 sec
RG 228.1
DW 7.800 usec
DE 6.00 usec
TE 296.8 K
D1 0.00000400 sec
d11 0.03000000 sec
TD0 1

======== CHANNEL f1 ========
NUC1 11B
P1 8.00 usec
PL1 -2.00 dB
SFO1 160.5257140 MHz

======== CHANNEL f2 ========
CPDPRG[2 waltz16
NUC2 off
PCPD2 100.00 usec
PL2 0 dB
PL12 17.52 dB
SFO2 500.3300000 MHz

F2 - Processing parameters
SI 32768
SF 160.5256909 MHz
WDW EM
SSB 0
LB 10.00 Hz
GB 0
PC 1.40

11B{1H} NMR

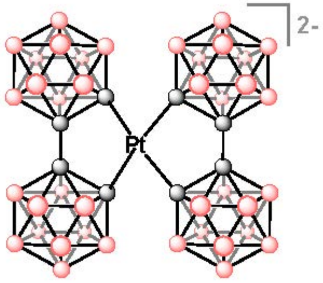

[Pt(bc)2]2-

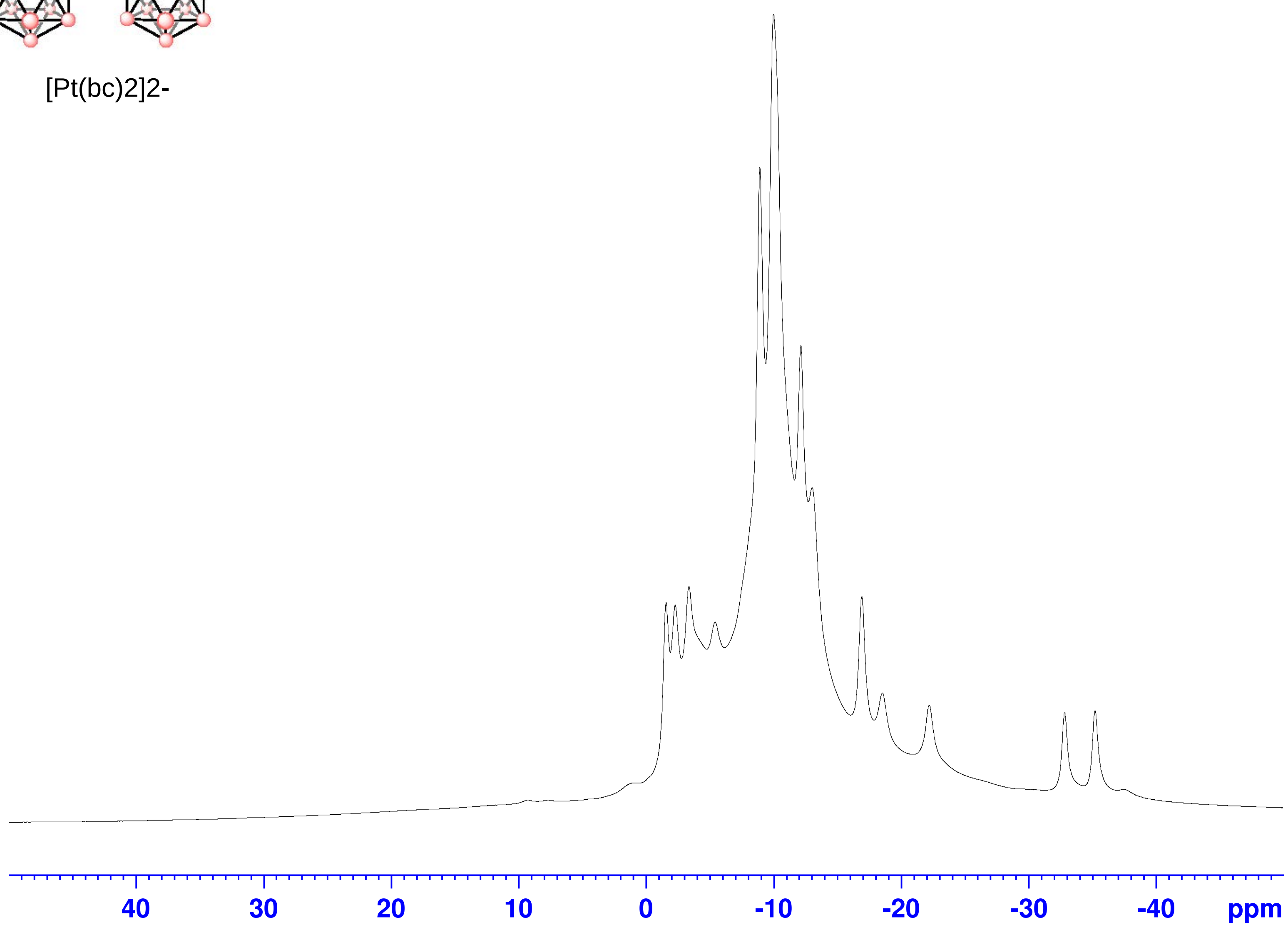


```
Current Data Parameters
NAME      KK-2-017 [Pt(bc)2]2- 11B
EXPNO                    2
PROCNO                   1

F2 - Acquisition Parameters
Date_             20160206
Time                 17.21
INSTRUM             drx500
PROBHD     5 mm bb-Z Z800
PULPROG               zgdc
TD                    7168
SOLVENT             CD2Cl2
NS                    8192
DS                       4
SWH              64102.562 Hz
FIDRES            8.942880 Hz
AQ               0.0559104 sec
RG                   228.1
DW                   7.800 usec
DE                    6.00 usec
TE                   297.1 K
D1              0.00000400 sec
d11             0.03000000 sec
TD0                      1

======== CHANNEL f1 ========
NUC1                   11B
P1                    8.00 usec
PL1                  -2.00 dB
SFO1           160.5257136 MHz

======== CHANNEL f2 ========
CPDPRG[2           waltz16
NUC2                    1H
PCPD2               100.00 usec
PL2        0 dB
PL12                 17.52 dB
SFO2           500.3330020 MHz

F2 - Processing parameters
SI                   32768
SF             160.5256909 MHz
WDW                     EM
SSB        0
LB                   10.00 Hz
GB         0
PC                    1.40
```

13C{1H} NMR

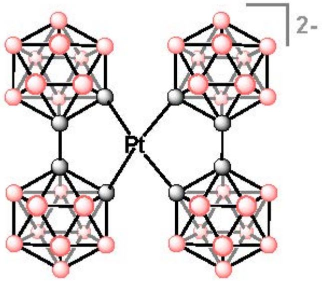

[Pt(bc)2]2-

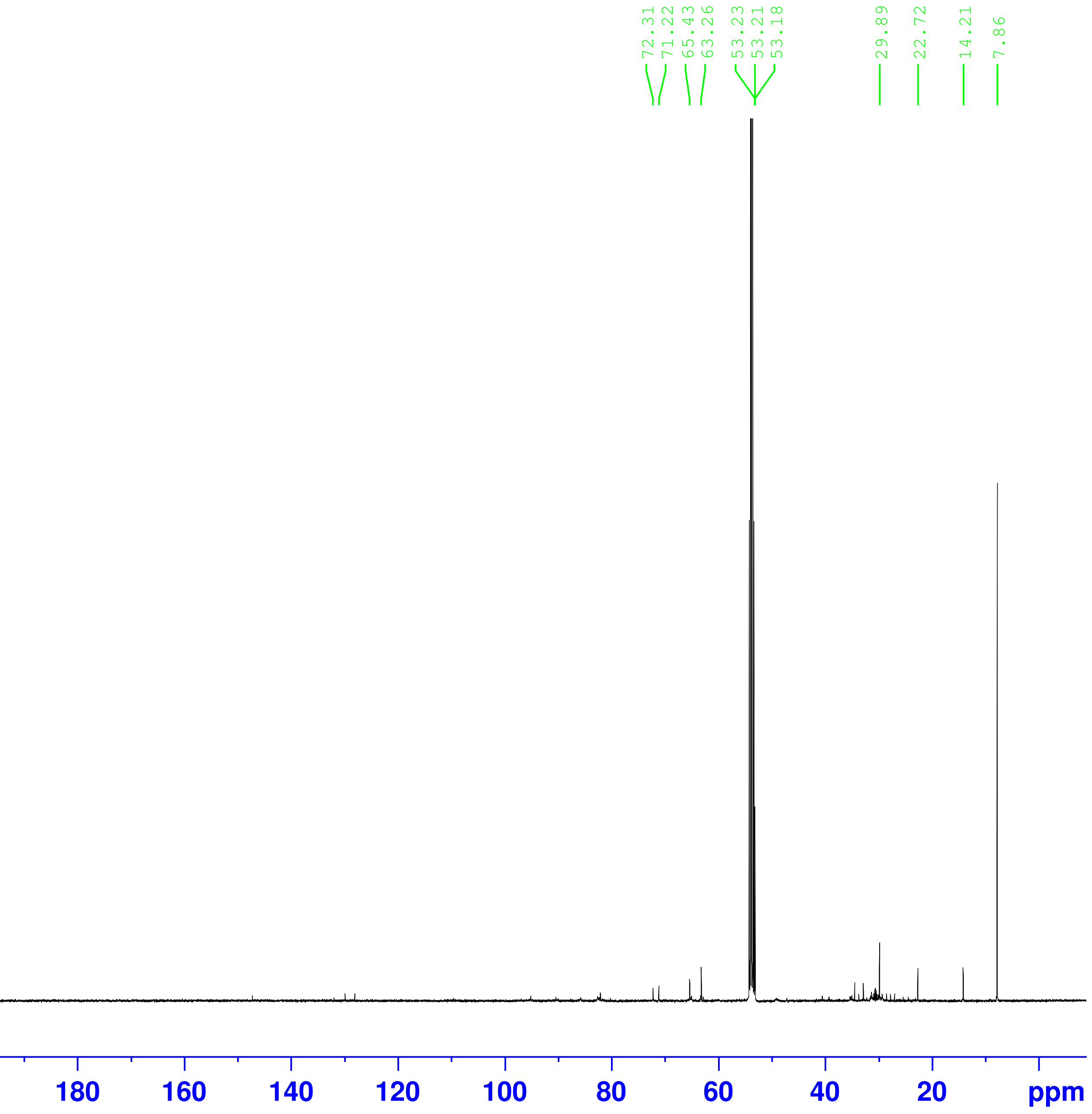


```
Current Data Parameters
NAME      KK-2-017 Pt(bc)2 vac dried
EXPNO                  11
PROCNO                  1

F2 - Acquisition Parameters
Date_            20160205
Time                18.06
INSTRUM             av500
PROBHD    5 mm DCH 13C-1
PULPROG           zgpg30
TD                 65536
SOLVENT           CD2Cl2
NS                  1024
DS                     2
SWH            31250.000 Hz
FIDRES          0.476837 Hz
AQ             1.0485760 sec
RG                204.54
DW                16.000 usec
DE                 18.00 usec
TE                 298.0 K
D1            2.00000000 sec
D11           0.03000000 sec
TD0                    1

======== CHANNEL f1 ========
SFO1         125.7722511 MHz
NUC1                 13C
P1                  9.63 usec
PLW1         23.00000000 W

======== CHANNEL f2 ========
SFO2         500.1330008 MHz
NUC2                  1H
CPDPRG[2           waltz16
PCPD2              80.00 usec
PLW2         13.50000000 W
PLW12         0.21094000 W
PLW13         0.13500001 W

F2 - Processing parameters
SI                131072
SF           125.7577397 MHz
WDW                   EM
SSB       0
LB                  1.00 Hz
GB        0
PC                  1.40
```

1H NMR

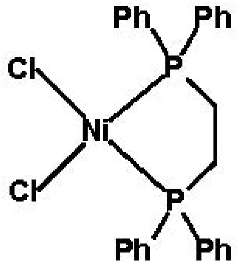

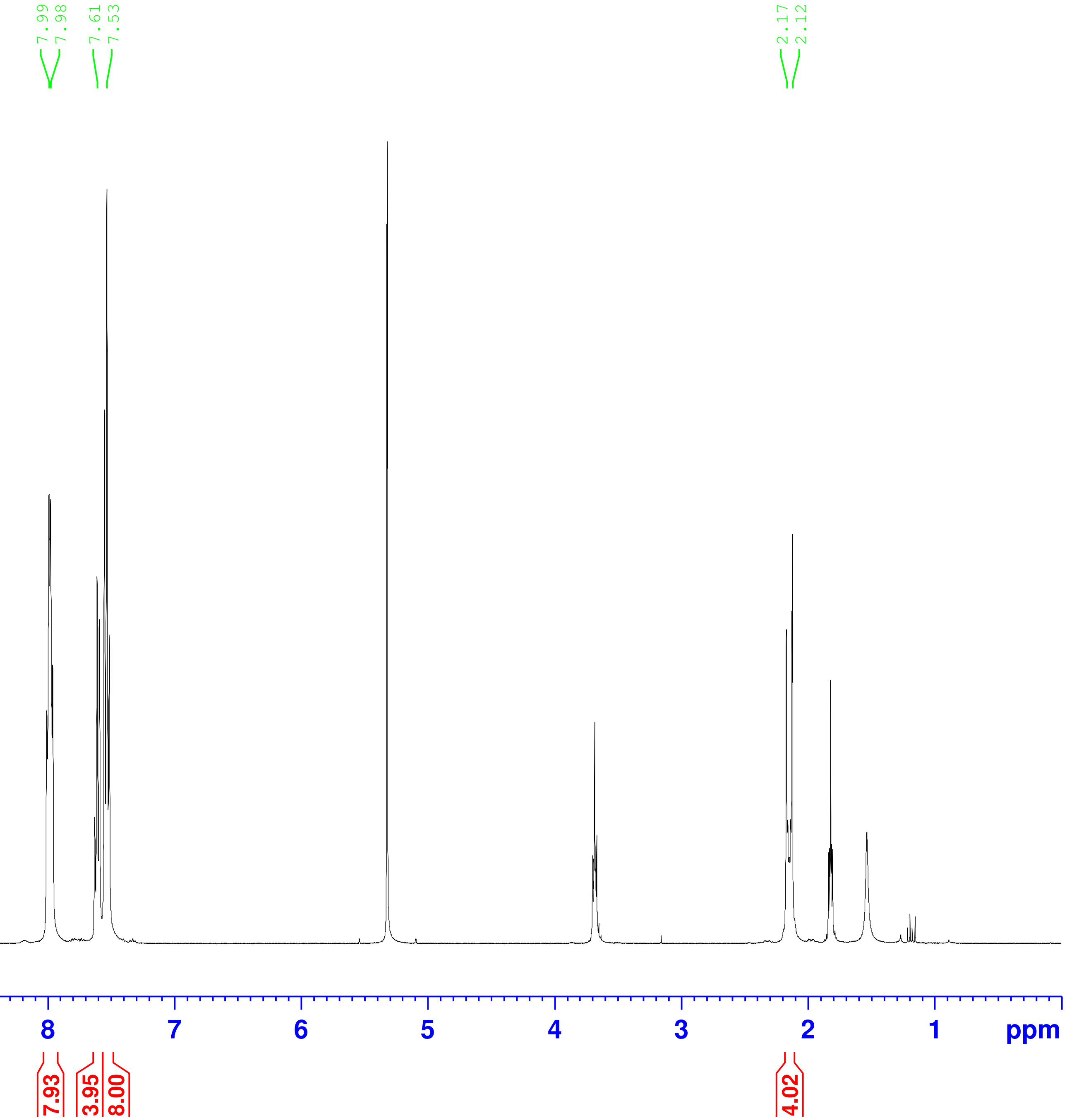


```
Current Data Parameters
NAME          Ni(dppe)Cl2
EXPNO                 170
PROCNO                  1

F2 - Acquisition Parameters
Date_            20150327
Time                12.57
INSTRUM             av400
PROBHD   5 mm PABBO BB/
PULPROG              zg30
TD                  52882
SOLVENT            CD2Cl2
NS                     64
DS                      0
SWH              8012.820 Hz
FIDRES           0.151523 Hz
AQ              3.2998369 sec
RG                 155.85
DW                 62.400 usec
DE                   6.50 usec
TE                  299.1 K
D1             2.00000000 sec
TD0                     1

======== CHANNEL f1 ========
SFO1          400.1324008 MHz
NUC1                   1H
P1                  15.00 usec
PLW1          13.00000000 W

F2 - Processing parameters
SI                  65536
SF            400.1300154 MHz
WDW                    EM
SSB      0
LB                   0.30 Hz
GB       0
PC                   1.00
```

31P{1H} NMR

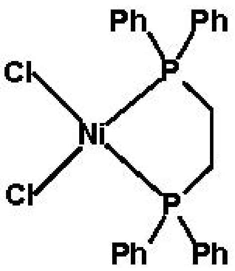

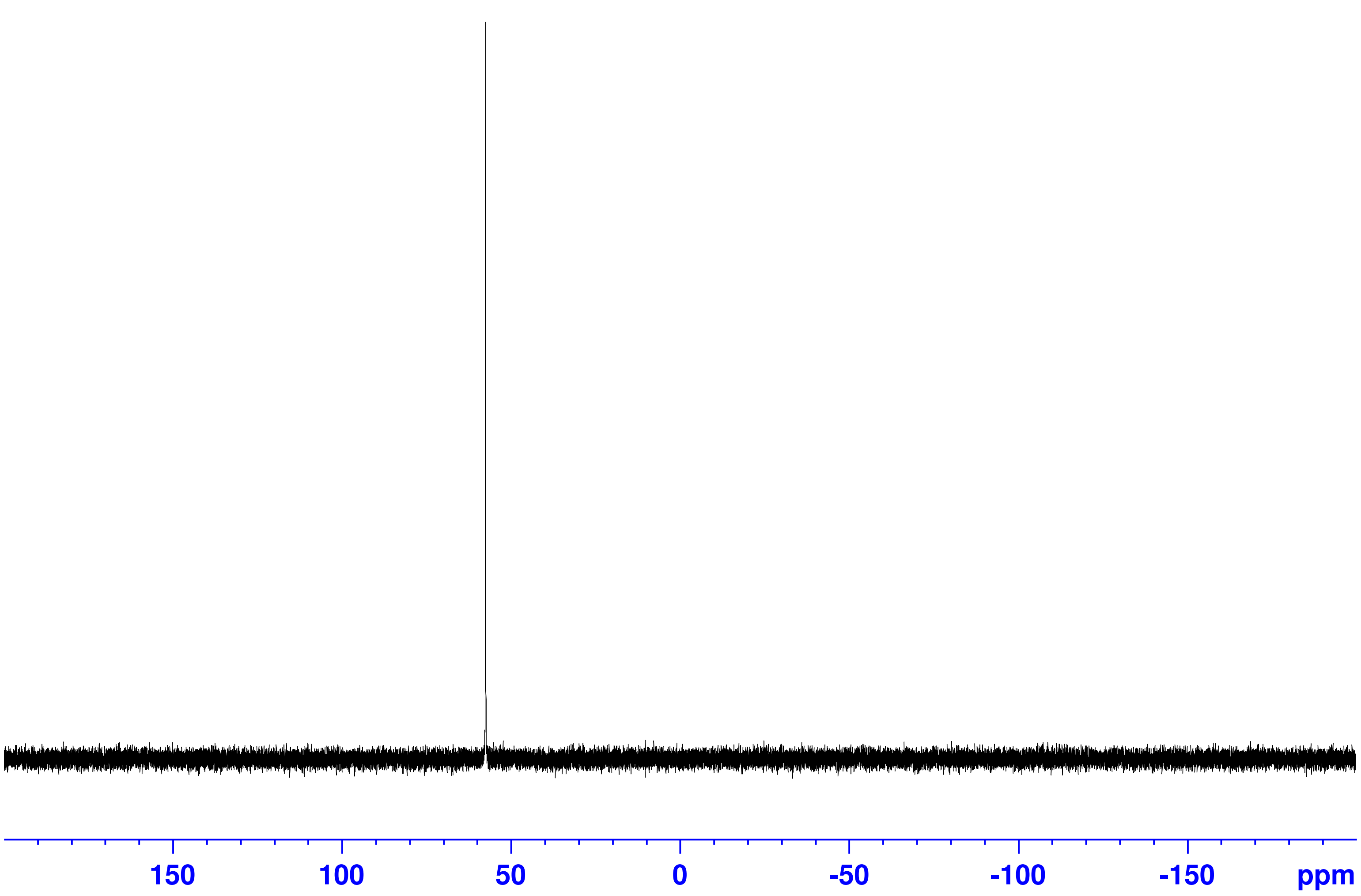


```
Current Data Parameters
NAME          Ni(dppe)Cl2
EXPNO                 171
PROCNO                  1

F2 - Acquisition Parameters
Date_            20150327
Time                13.02
INSTRUM             av400
PROBHD    5 mm PABBO BB/
PULPROG           zgpg30
TD                262144
SOLVENT           CD2Cl2
NS                    80
DS                     0
SWH           131578.953 Hz
FIDRES          0.501934 Hz
AQ             0.9961472 sec
RG                189.85
DW                 3.800 usec
DE                  6.50 usec
TE                 300.1 K
D1            2.00000000 sec
D11           0.03000000 sec
TD0                    1

======== CHANNEL f1 ========
SFO1         161.9755930 MHz
NUC1                 31P
P1                 14.00 usec
PLW1         12.00000000 W

======== CHANNEL f2 ========
SFO2         400.1324008 MHz
NUC2                  1H
CPDPRG[2         waltz16
PCPD2              90.00 usec
PLW2         13.00000000 W
PLW12         0.36111000 W
PLW13         0.29249999 W

F2 - Processing parameters
SI                262144
SF           161.9755930 MHz
WDW                   EM
SSB       0
LB                  1.00 Hz
GB        0
PC                  1.40
```

1H NMR

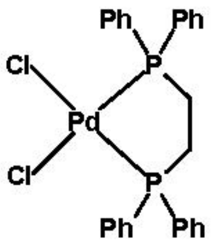

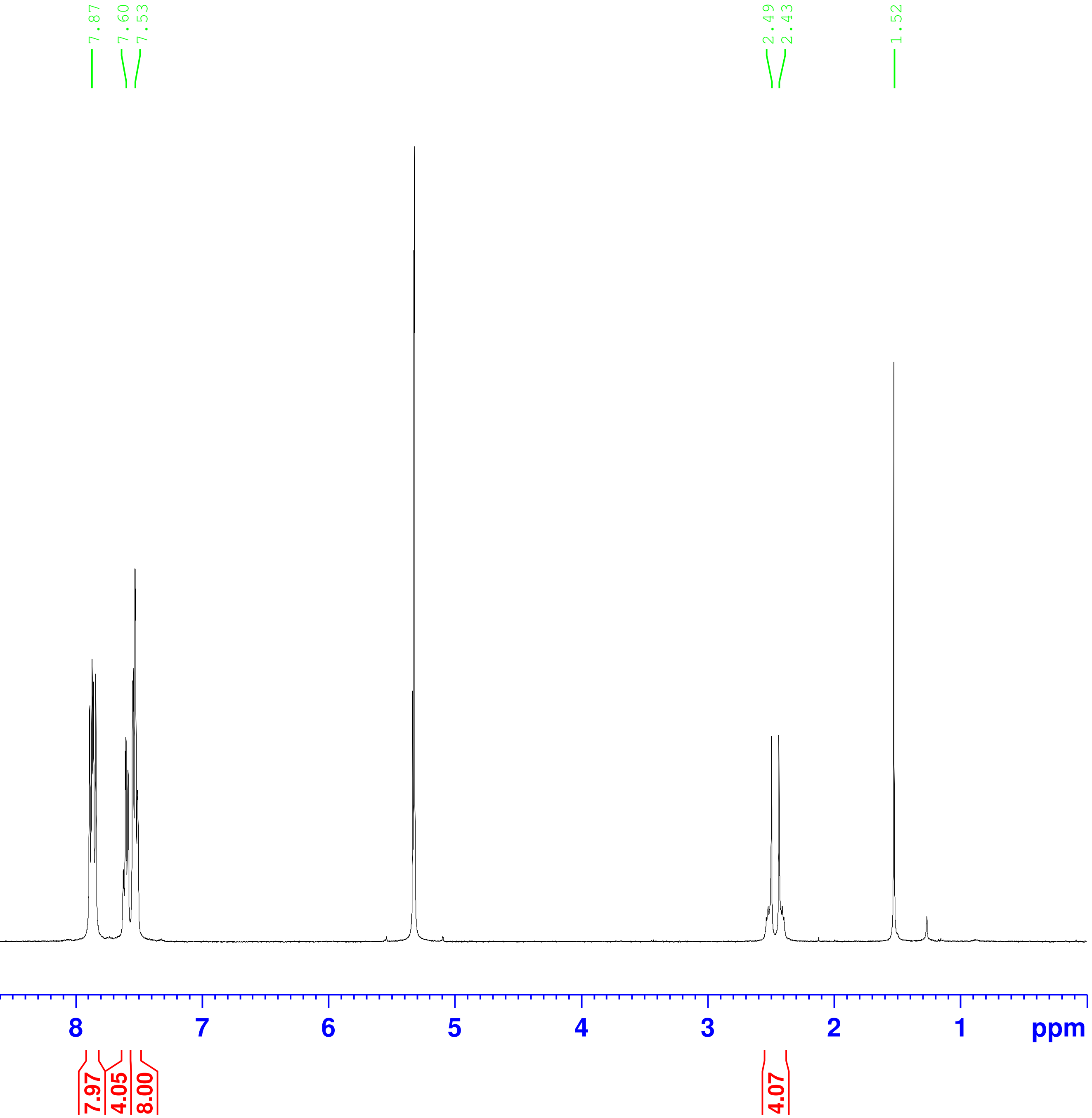


```
Current Data Parameters
NAME             Pd(dppe)Cl2
EXPNO                     60
PROCNO                     1

F2 - Acquisition Parameters
Date_               20150324
Time                   10.37
INSTRUM                av400
PROBHD    5 mm PABBO BB/
PULPROG                 zg30
TD                     52882
SOLVENT               CD2Cl2
NS                        40
DS                         0
SWH                 8012.820 Hz
FIDRES              0.151523 Hz
AQ                 3.2998369 sec
RG                    155.85
DW                    62.400 usec
DE                      6.50 usec
TE                     299.0 K
D1                2.00000000 sec
TD0                        1

======== CHANNEL f1 ========
SFO1          400.1324008 MHz
NUC1                      1H
P1                     15.00 usec
PLW1             13.00000000 W

F2 - Processing parameters
SI                     65536
SF            400.1300155 MHz
WDW                       EM
SSB        0
LB                      0.30 Hz
GB         0
PC                      1.00
```

$^{31}P\{^{1}H\}$ NMR

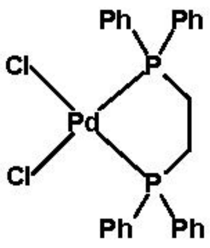

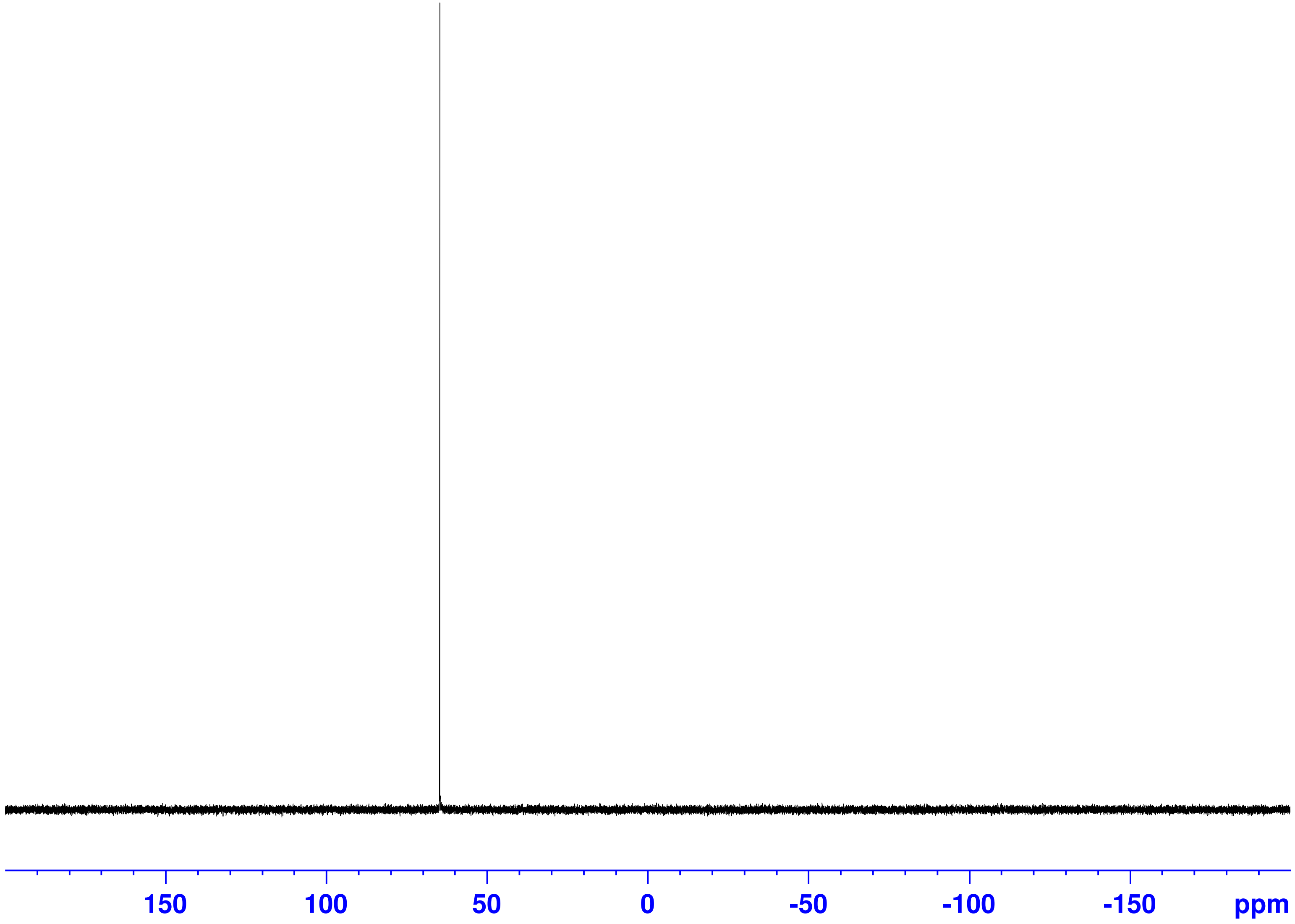

Current Data Parameters
NAME Pd(dppe)Cl2
EXPNO 61
PROCNO 1

F2 - Acquisition Parameters
Date_ 20150324
Time 10.42
INSTRUM av400
PROBHD 5 mm PABBO BB/
PULPROG zgpg30
TD 262144
SOLVENT CD2Cl2
NS 64
DS 0
SWH 131578.953 Hz
FIDRES 0.501934 Hz
AQ 0.9961472 sec
RG 189.85
DW 3.800 usec
DE 6.50 usec
TE 299.0 K
D1 2.00000000 sec
D11 0.03000000 sec
TD0 1

======== CHANNEL f1 ========
SFO1 161.9755930 MHz
NUC1 31P
P1 14.00 usec
PLW1 12.00000000 W

======== CHANNEL f2 ========
SFO2 400.1324008 MHz
NUC2 1H
CPDPRG[2 waltz16
PCPD2 90.00 usec
PLW2 13.00000000 W
PLW12 0.36111000 W
PLW13 0.29249999 W

F2 - Processing parameters
SI 262144
SF 161.9755930 MHz
WDW EM
SSB 0
LB 1.00 Hz
GB 0
PC 1.40

1H NMR

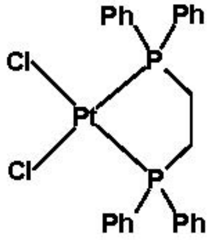

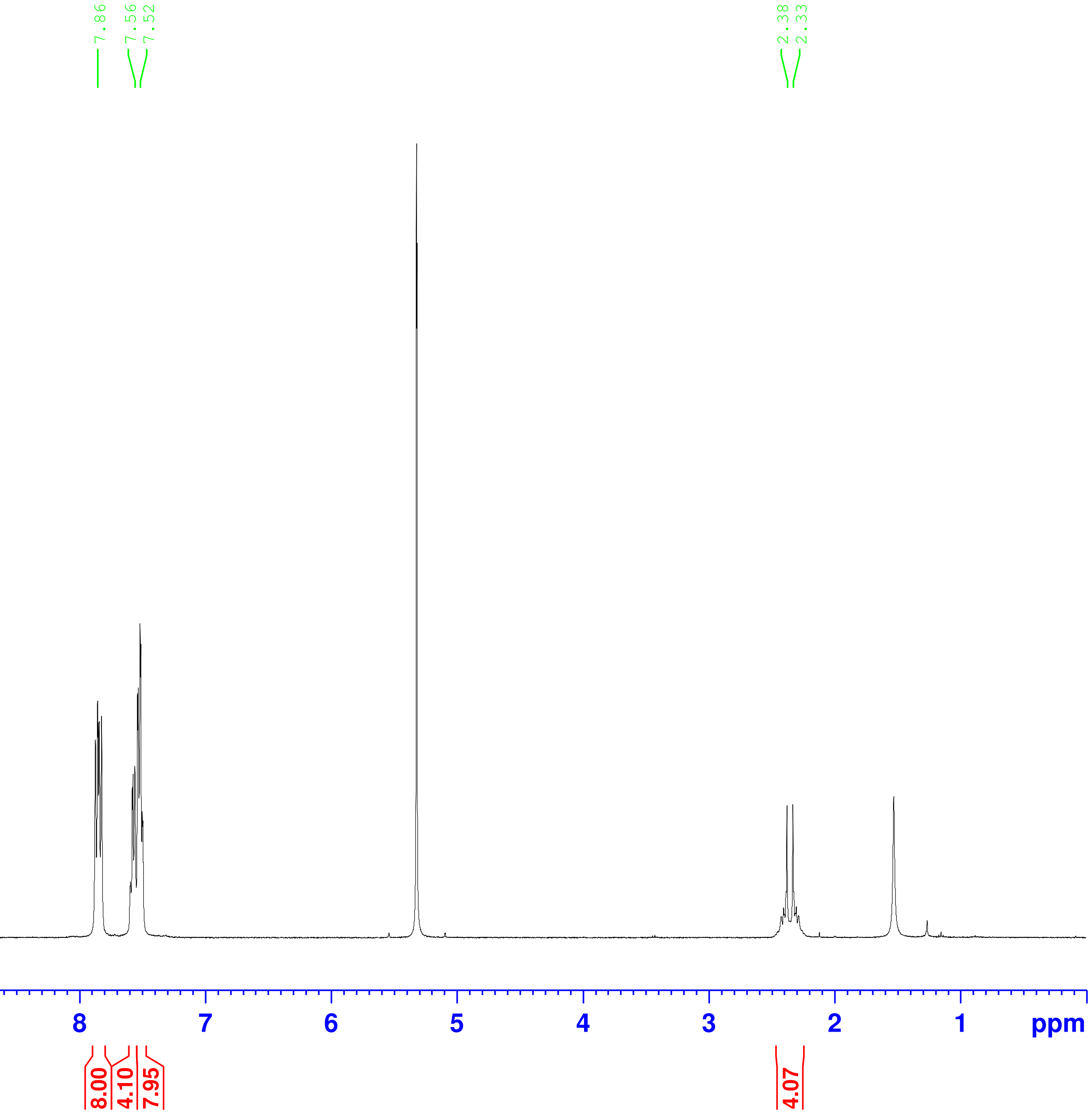

Current Data Parameters
NAME Pt(dppe)Cl2
EXPNO 50
PROCNO 1

F2 - Acquisition Parameters
Date_ 20150324
Time 10.25
INSTRUM av400
PROBHD 5 mm PABBO BB/
PULPROG zg30
TD 52882
SOLVENT CD2Cl2
NS 40
DS 0
SWH 8012.820 Hz
FIDRES 0.151523 Hz
AQ 3.2998369 sec
RG 189.85
DW 62.400 usec
DE 6.50 usec
TE 299.0 K
D1 2.00000000 sec
TD0 1

======== CHANNEL f1 ========
SFO1 400.1324008 MHz
NUC1 1H
P1 15.00 usec
PLW1 13.00000000 W

F2 - Processing parameters
SI 65536
SF 400.1300153 MHz
WDW EM
SSB 0
LB 0.30 Hz
GB 0
PC 1.00

31P{1H} NMR

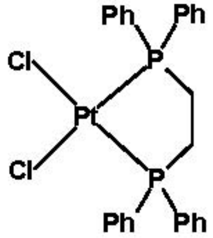

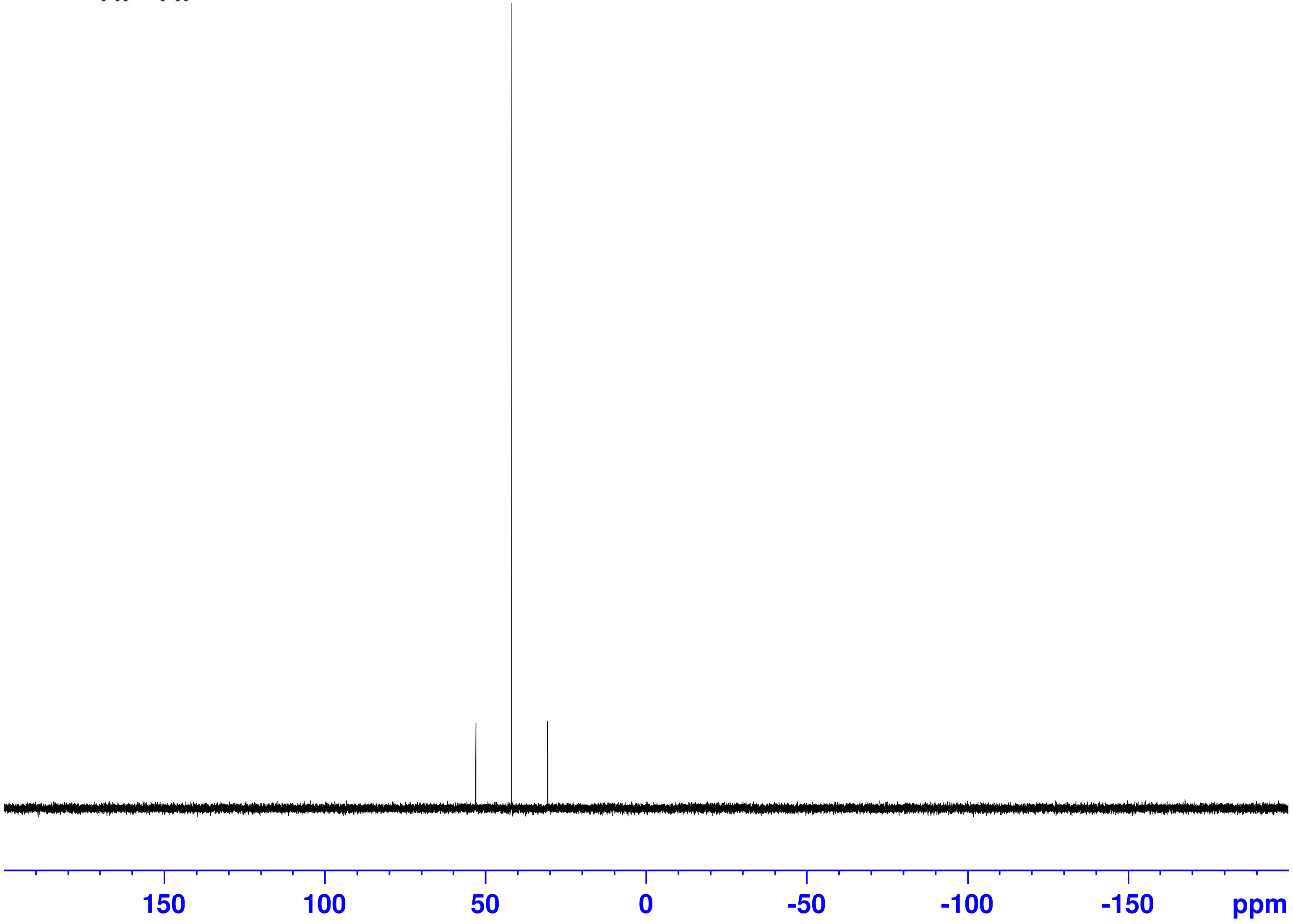

Current Data Parameters	
NAME	Pt(dppe)Cl2
EXPNO	51
PROCNO	1
F2 - Acquisition Parameters	
Date_	20150324
Time	10.29
INSTRUM	av400
PROBHD	5 mm PABBO BB/
PULPROG	zgpg30
TD	262144
SOLVENT	CD2Cl2
NS	64
DS	0
SWH	131578.953 Hz
FIDRES	0.501934 Hz
AQ	0.9961472 sec
RG	189.85
DW	3.800 usec
DE	6.50 usec
TE	299.0 K
D1	2.00000000 sec
D11	0.03000000 sec
TD0	1
======== CHANNEL f1 ========	
SFO1	161.9755930 MHz
NUC1	31P
P1	14.00 usec
PLW1	12.00000000 W
======== CHANNEL f2 ========	
SFO2	400.1324008 MHz
NUC2	1H
CPDPRG[2	waltz16
PCPD2	90.00 usec
PLW2	13.00000000 W
PLW12	0.36111000 W
PLW13	0.29249999 W
F2 - Processing parameters	
SI	262144
SF	161.9755930 MHz
WDW	EM
SSB	0
LB	1.00 Hz
GB	0
PC	1.40

1H NMR

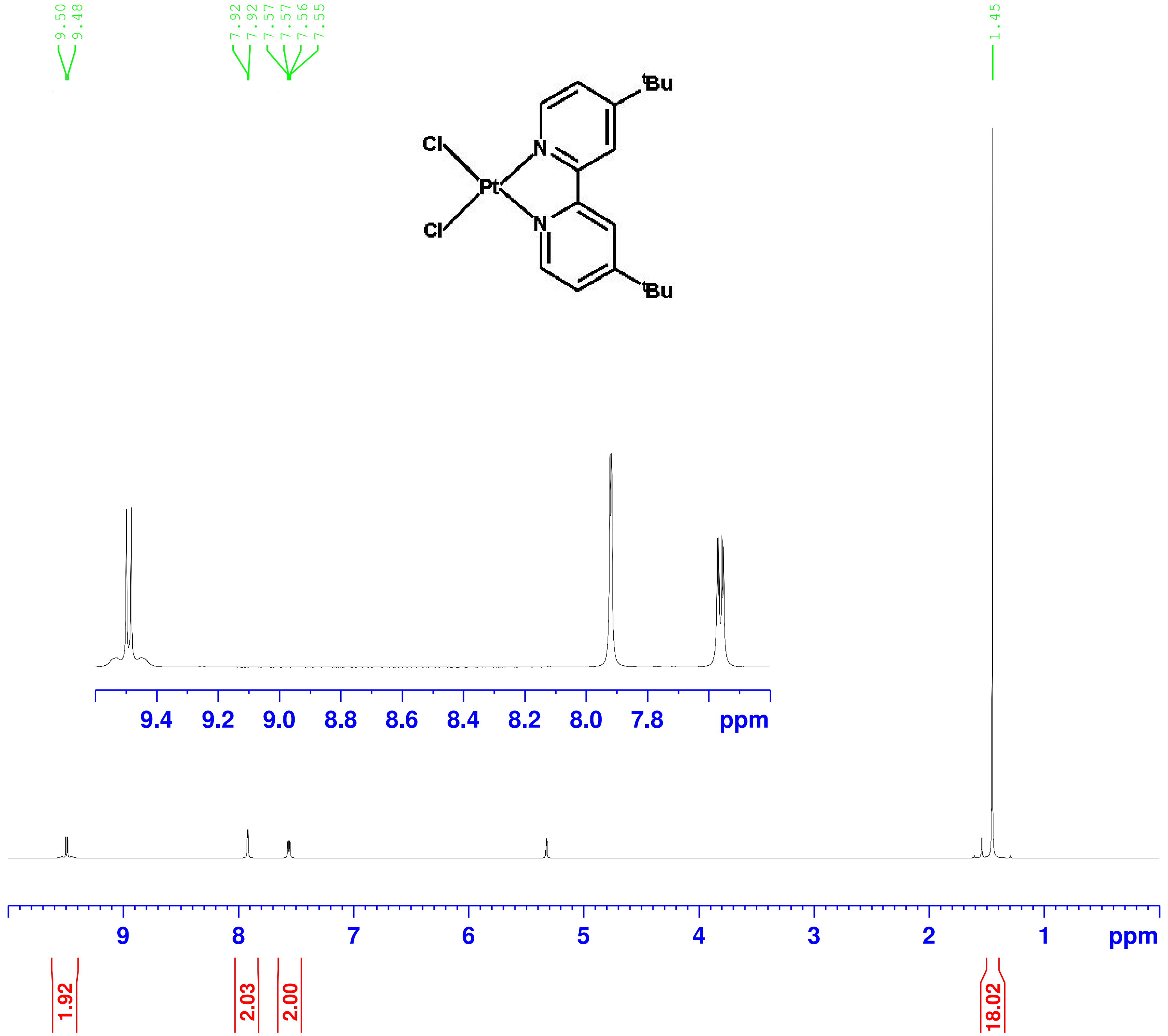

Current Data Parameters
NAME Pt(dtb-bpy)Cl2, DCM
EXPNO 10
PROCNO 1

F2 - Acquisition Parameters
Date_ 20150801
Time 15.01
INSTRUM av400
PROBHD 5 mm PABBO BB/
PULPROG zg30
TD 52882
SOLVENT CD2Cl2
NS 64
DS 0
SWH 8012.820 Hz
FIDRES 0.151523 Hz
AQ 3.2998369 sec
RG 155.85
DW 62.400 usec
DE 6.50 usec
TE 299.0 K
D1 2.00000000 sec
TD0 1

======== CHANNEL f1 ========
SFO1 400.1324008 MHz
NUC1 1H
P1 15.00 usec
PLW1 13.00000000 W

F2 - Processing parameters
SI 65536
SF 400.1300154 MHz
WDW EM
SSB 0
LB 0.30 Hz
GB 0
PC 1.00
